# Dietary zinc restriction affects the expression of genes related to immunity and stress response in the small intestine of pigs

**DOI:** 10.1017/jns.2022.105

**Published:** 2022-11-22

**Authors:** Ramya Lekha Medida, Ashok Kumar Sharma, Yue Guo, Lee Johnston, Pedro E. Urriola, Andres Gomez, Milena Saqui-Salces

**Affiliations:** 1 Department of Animal Science, University of Minnesota, 1988 Fitch Ave., 495K AS/VM, Saint Paul, MN 55108, USA; 2 West Central Research and Outreach Center (WCROC), University of Minnesota, Morris, MN, USA

**Keywords:** Gene expression, RNA-seq, Zinc deficiency, Zinc transporters, ZIP4

## Abstract

Zinc (Zn) is an essential mineral and its deficiency manifests in non-specific clinical signs that require long time to develop. The response of swine intestine to Zn restriction was evaluated to identify early changes that can be indicative of Zn deficiency. Twenty-seven pigs (body weight = 77⋅5 ± 2⋅5 kg) were assigned to one of three diets: diet without added Zn (Zn-restricted diet, ZnR), and ZnR-supplemented with either 50 (Zn50) or 100 mg of Zn/kg of diet (Zn100) of Zn supplied by ZnCl_2_. After 32 d consuming the diets, serum Zn concentration in ZnR pigs was below the range of 0⋅59–1⋅37 μg/ml considered sufficient, thereby confirming subclinical Zn deficiency. Pigs showed no obvious health or growth changes. RNA-seq analysis followed by qPCR showed decreased expression of *metallothionein-1* (*MT1*) (*P* < 0⋅05) and increased expression of Zn transporter *ZIP4* (*P* < 0⋅05) in jejunum and ileum of ZnR pigs compared with Zn-supplemented pigs. Ingenuity pathway analysis revealed that Zn50 and Zn100 induced changes in genes related to nucleotide excision repair and integrin signalling pathways. The top gene network in the ZnR group compared with Zn100 was related to lipid and drug metabolism; and compared with Zn50, was related to cellular proliferation, assembly and organisation. Dietary Zn concentrations resulted in differences in genes related to immune pathways. Our analysis showed that small intestine presents changes associated with Zn deficiency after 32 d of Zn restriction, suggesting that the intestine could be a sentinel organ for Zn deficiency.

## Introduction

Zinc (Zn) is a vitally important trace mineral for all life forms, and it is involved in numerous physiological functions including growth and development, cell differentiation, immune response, enzyme activation and maintenance of structural integrity of the genome^(1–4)^. Humans and other mammals must consistently obtain Zn through their diet^(5)^. The U.S. National Institutes of Health reported in 2020 that 35–40 % of adults in the United States had Zn intake below the required 6⋅8–9⋅4 mg/d^(6)^. According to the World Health Organization, the global prevalence of Zn deficiency is 31 %^(7)^. For domestic and production animals, Zn is supplemented in the diet at levels established for each species.

Clinical manifestations of dietary Zn deficiency include growth retardation, loss of appetite, impaired wound healing, immunodeficiency and dermatological lesions^(3,8)^. However, these signs are non-specific and develop over an extended period. Therefore, the assessment of Zn nutritional status using clinical signs is difficult, especially in conditions of low or marginal Zn intake^(2,9)^. Presently, plasma Zn concentration (PZC) is used as an indicator of Zn status despite high inter-individual differences and variability due to inflammation, administration of drugs, recent meal consumption and malnutrition^(2,10–12)^. Different organs may show functional changes related to Zn deficiency before clinical signs of deficiency are noticeable. Some of these functional changes induced by Zn restriction that affect overall health^(13)^ include increased DNA damage^(14)^, oxidative stress^(15)^, damage to epithelial barrier^(16)^ and impaired activity of immune cells^(4,17)^. Therefore, it is important to identify early Zn restriction to prevent health issues from aggravating.

Manipulating Zn levels in humans poses technical difficulties and ethical dilemmas. Genetically and physiologically, pigs closely resemble humans^(18)^ and their use has been proven valuable in addressing human nutritional and pathophysiological issues^(19,20)^. Furthermore, Zn absorption and storage in pigs is comparable to that reported in humans^(20–23)^. Because of the importance of Zn in growth and development, dietary requirements for Zn in pigs at different stages of life have been studied extensively^(24–27)^. The National Research Council (NRC) has summarised dietary requirements of pigs to be 100 mg of Zn/kg of diet for weanlings up to 10 kg of body weight (BW), and 50 mg of Zn/kg of diet for growing and finishing pigs over 50 kg BW^(28)^. These dietary concentrations were defined as the required Zn supplementation for optimal growth (body mass) of pigs fed grain-based diets in the absence of disease. There is limited information on specific dietary Zn requirements for optimal immune and stress responses and a generalised idea that supplementing at levels greater than the requirement for growth will be beneficial; however, this practice may have a detrimental impact on the environment^(28)^.

The small intestine is the major site of nutrient absorption and one of the first organs to be exposed to dietary changes, making it an ideal organ for nutrigenomic studies. The small intestine responds to beneficial as well as harmful luminal signals from the environment and thus plays an important role in gut health and the overall welfare of the animal. Understanding intestinal responses of swine to Zn supplementation can help understand early changes associated with Zn deficiency before clinical signs are observed. In the present study, we evaluated gene expression profiles of swine intestine in response to Zn restriction and Zn supplementation provided by zinc chloride (ZnCl_2_).

## Experimental methods

### Ethical approval

Animal procedures were approved by and performed under the supervision of the Institutional Animal Care and Use Committee of the University of Minnesota, protocol: #1709-35112A.

### Animal management

The experiment was conducted at the University of Minnesota West Central Research and Outreach Center (WCROC) in Morris, MN. Twenty-seven Landrace/Yorkshire crossbred finisher pigs (13 males and 14 females, BW = 77⋅5 ± 2⋅5 kg, age = 17–18 weeks) were randomly assigned to three pens with nine pigs per pen. Pigs were housed in pens (1⋅6 × 4⋅5 m) equipped with slatted concrete floors and nipple drinkers. All pigs had *ad libitum* access to feed and water throughout the experiment. Pens were assigned to experimental diets and pigs in each pen were fed with one of three diets for 32 d. BW and feed disappearance determined for the pen was recorded weekly to calculate average daily gain (ADG) and estimate average daily feed intake (ADFI) of individual pigs.

### Diets

Pigs were fed with Zn sufficient diets before start of the experiment. Experimental diets were formulated to meet or exceed NRC nutritional recommendations^(28)^ for pigs weighing 50–75 kg, except for Zn. The basal diet, formulated with maize, soyabean meal (46 % protein), soyabean oil (Table 1) and a Zn-free vitamin and trace mineral premix (Table 2), was supplemented with either 50 mg of Zn/kg of diet or 100 mg of Zn/kg of diet from ZnCl_2_ for the experimental diets. The Zn-free vitamin and trace mineral premix was obtained from Agri Nutrition Inc. (Shakopee, MN) and Zn products were obtained from Zinpro Corporation (Eden Prairie, MN). Other ingredients were sourced locally.

**Table 1.** Ingredient and nutrient composition of the basal diet (as fed basis)^a^

Item	Diet composition
Ingredients (%)	
Maize	68⋅15
Soyabean meal	27⋅50
Soyabean oil	1⋅00
Zn-free vitamin and trace mineral premix	3⋅35
TOTAL	100⋅00
Calculated (%)	
Dry matter	85⋅93
Crude protein	18⋅74
Metabolisable energy (MJ/kg)	13⋅84
SID AA (%)	
Lys	0⋅88
Met	0⋅27
Thr	0⋅58
Trp	0⋅20
Leu	1⋅45
Ile	0⋅68
Val	0⋅75
Calcium (%)	0⋅77
Phosphorus (%)	0⋅66

Note: To prepare treatment diets, the basal zinc-restricted diet was supplemented with either 50 mg of Zn/kg of diet or 100 mg of Zn/kg of diet from ZnCl_2_.

SID AA, Standardised ileal digestible amino acids.

a Formulated based on metabolisable energy values for maize and soyabean meal.

**Table 2.** Composition of the Zn-free vitamin and trace mineral^a^ premix

Vitamin/trace mineral	Concentration per kg of mix
Vitamin A	73 546 μg retinol
Vitamin D3	1149 μg cholecalciferol
Vitamin E	827 mg dl-alpha-tocopherol
Vitamin K	92 mg
Niacin	919 mg
Pantothenic acid	613 mg
Riboflavin	153 mg
Vitamin B12	919 μg
Copper	107 mg
Iodine	8⋅2 mg
Iron	917 mg
Manganese	153 mg
Selenium	8⋅2 mg
Calcium	21⋅4 % of mix
Phosphorus	8⋅53 % of mix
Salt	12⋅4 % of mix

a Trace mineral sources: Iodine (ethylenediamine dihydroiodide), polysaccharide Fe, Mn and Cu complexes, and selenium selenite.

The dietary treatments were as follows: (1) Zn-restricted diet (ZnR): Basal diet containing Zn-free vitamin–mineral mix; (2) Zn50: ZnR + 50 mg of Zn/kg of diet from ZnCl_2_ and (3) Zn100: ZnR + 100 mg of Zn/kg of diet from ZnCl_2_.

Feed samples were collected at the time of manufacture and analysed for total Zn concentration at the Soil Testing Laboratory, University of Minnesota by inductively coupled plasma optical emission spectrometry (ICP-OES)^(29)^.

### Sample collection

On day 32 of the experiment, blood samples were collected from the jugular vein and centrifuged at 2000×*g* for 10 min in a refrigerated centrifuge at 4°C. Sera were obtained and stored at −80°C until analysis. Serum Zn concentrations were measured using an inductively coupled plasma–mass spectrometry (ICP-MS) technique at Michigan State University Veterinary Diagnostic Laboratory.

All pigs were euthanised on day 32 by captive bolt followed by exsanguination. Intestinal jejunal (1 m distal to pyloric sphincter) and ileal (15 cm proximal to ileocecal valve) samples were collected immediately after euthanasia and snap frozen in liquid nitrogen and stored at −80°C for subsequent analysis.

### RNA extraction and sequencing

Total RNA was extracted from ileal and jejunal samples using the Qiagen RNeasy Mini Kit (Qiagen, Germantown, MD, USA). The quantity of RNA was determined using a Nanodrop 2000 (ThermoFisher Scientific, USA). Extracted RNA of the 27 ileal samples were submitted to the University of Minnesota Genomic Center for library construction and sequencing. Sequencing was run on the Illumina HiSeq 2500 platform after library prep using TrueSeq stranded RNA kit to generate paired end reads of the length of 125 base pairs. Four samples were removed from the study due to low RNA quality not suitable for sequencing (two samples from the ZnR group, and one sample each from the Zn50 and Zn100 groups). Extracted RNA from the 27 jejunal samples were used for gene expression analysis through qPCR.

### Quality control, trimming and alignment of reads

Quality of the reads was examined using FastQC tool kit (https://www.bioinformatics.babraham.ac.uk/projects/fastqc/). Adapter sequences and low-quality bases were trimmed using Trimmomatic^(30)^ such that average base quality was not less than 20 for every sliding window of four bases and to keep the minimum read length of 86. Trimmed paired end sequences were mapped to the pig reference genome Sscrofa 11.1 (Ensembl) using Kallisto^(31)^. Transcripts per million measures for each gene were used for downstream statistical analysis. Genes which were not detected in at least 5 % (*n* 2) of the samples were excluded resulting in a total of 38 751 identified genes. The raw and processed data have been deposited in the NCBI Gene Expression Omnibus (GEO) under accession number GSE181343.

### Differential gene expression analysis and quantitative PCR (qPCR)

Differential expression analysis was performed using the DESeq2 package in R^(32,33)^. Log fold change values and *P*-values were generated for comparisons between groups. Network and pathway analyses were conducted using PANTHER^(34)^ and ingenuity pathway analyses (IPA) software (QIAGEN Inc.)^(35)^.

To verify reliability of RNA-seq results, four genes related to zinc transport and immune functions were selected for qPCR. Briefly, after total RNA extraction from ileal and jejunal samples, 500 ng were used for reverse transcription to cDNA using the High Capacity cDNA Reverse Transcription kit (ThermoFisher Scientific, USA) with random hexamer primers. The qPCR reactions were formulated with 100 nM of forward and reverse primers and 10 ng of cDNA template using PowerUp SYBR Green PCR Master Mix (ThermoFisher Scientific, USA). Amplification was performed under the following conditions: initial activation at 95°C for 10 min, followed by 40 cycles of denaturation at 95°C for 15 s and annealing at 60°C for 60 s. Glycerahdehyde-3-phosphate dehydrogenase (*GAPDH*) was used as the reference gene^(36–38)^ after confirmation that samples showed no more than one cycle difference in the *GAPDH* amplification^(39)^. Sequences of primers for the selected genes are presented in Table 3. The 2^−ΔΔCt^ method was used to calculate gene expression changes^(40)^.

**Table 3.** Primer sequences used for gene expression analysis in swine intestine

Gene^a^	Forward primer	Reverse primer
*GAPDH*	ATCCTGGGCTACACTGAGGAC	AAGTGGTCGTTGAGGGCAATG
*MT1*	GCTGTGCCTGATGTGACGAA	AGGAAGACGCTGGGTTGGT
*ZIP4*	CTGCACACACATGATGGGGA	GGTTGAAAAGGCTCTCGAACA

a *GAPDH*, glyceraldehyde-3-phosphate dehydrogenase; *MT1*, metallothionein1; *ZIP4*, Zrt- and Irt-like protein 4.

### Statistical analyses and justification of sample size

*A priori* sample size calculation performed using G*Power 3.1.9.6.^(^^41)^ indicated a minimum of four pigs per group were required to achieve significance in one-way ANOVA with a power of 0⋅80. Considering serum Zn as the primary outcome of the present study, *a posteriori* power analysis showed a power of 0⋅95.

Weight gain data were analysed using the GLM procedure of SAS (SAS Inst. Inc., Cary, NC) with each individual pig as the experimental unit. Diets and sex were considered as fixed effects, initial BW was considered a covariate. Comparisons among treatments were performed using Student–Newman–Keuls (SNK) for multiple comparison of means. Results are reported as least squares means. Treatment effects were considered significantly different at *P* ≤ 0⋅05.

One-way ANOVA with Kruskal–Wallis multiple comparisons test was used to compare differences in serum Zn concentration and gene expression among treatments using GraphPad Prism 8.1 software for Windows (GraphPad Software, USA). Data were expressed as the means with standard error (±sem). A *P* ≤ 0⋅05 was considered statistically significant.

## Results

### Diets

Treatment diets differed in Zn concentration as expected (Table 4), with the ZnR diet below the Zn requirement of 50 mg of Zn/kg of diet for swine of this BW^(28)^.

**Table 4.** Measured Zn concentrations in experimental diets (as fed basis)

Diet^a^	Zn concentration (mg of Zn/kg of diet)
ZnR	34
Zn50	88
Zn100	147

a ZnR, (Zn-free vitamin–mineral mix + 0 mg of Zn/kg of diet); Zn50, (Zn-free vitamin–mineral mix + 50 mg of Zn/kg of diet); Zn100, (Zn-free vitamin–mineral mix + 100 mg of Zn/kg of diet).

### Animal growth

Average daily weight gain and pen feed intake were not different among pigs fed the three dietary treatments (Supplementary Table S1; https://doi.org/10.6084/m9.figshare.15079074). The animals were in good health with no clinical manifestations of Zn deficiency or any other ailments throughout the experiment.

### Serum Zn levels

**Fig. 1.**
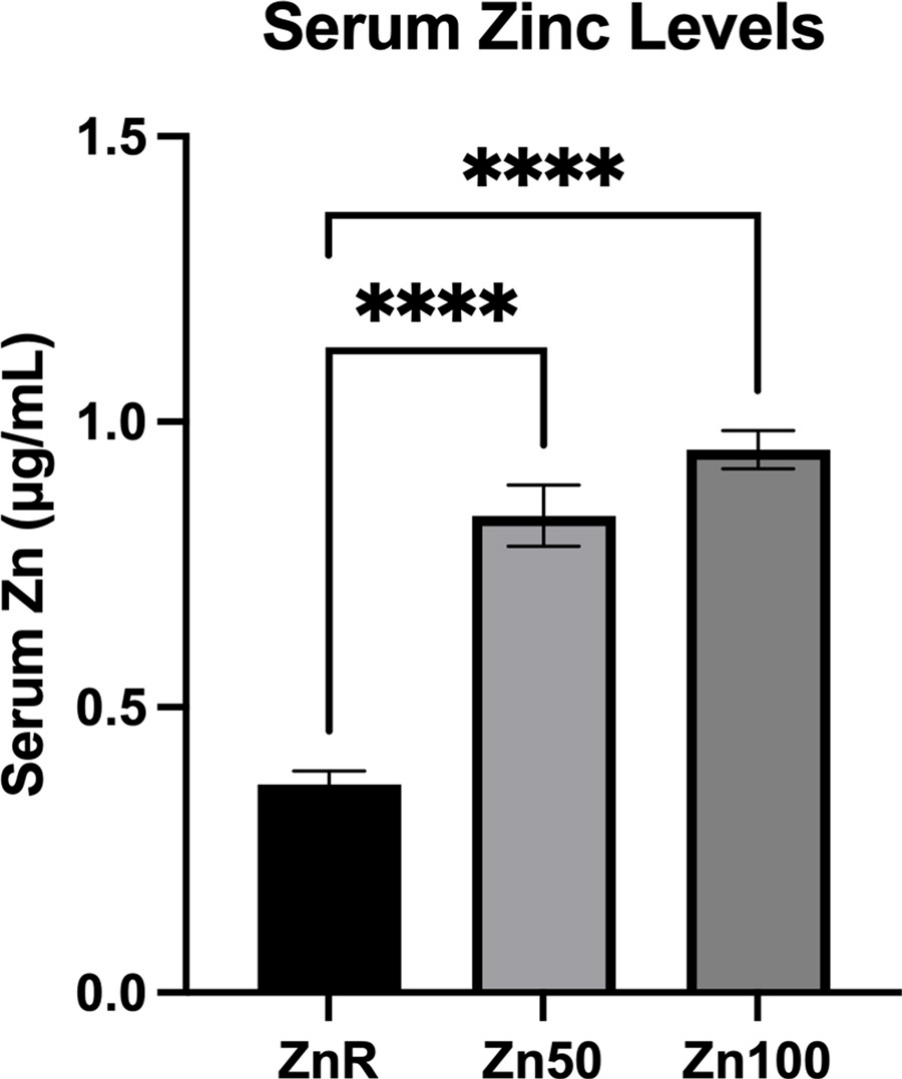
Serum Zn concentration of pigs fed Zn-restricted diets (ZnR), or diets supplemented with 50 and 100 mg of Zn/kg of diet from ZnCl_2_ (Zn50 and Zn100, respectively). Data are presented as means ± sem. ****P ≤ 0⋅0001. *n* 9 for all groups.

Mean serum Zn concentrations observed were 0⋅36, 0⋅83 and 0⋅95 μg/ml in pigs fed ZnR, Zn50 and Zn100 diets, respectively. Serum levels in the ZnR group of pigs were less than the serum levels of pigs fed Zn50 and Zn100 diets (*P* < 0⋅0001; Fig. 1).

### Differential gene expression and qPCR analysis

The total number of differentially expressed genes (DEGs) identified are presented in Table 5. Identities of DEGs and log2 fold changes are presented in Supplementary Tables S2 and S3 (https://doi.org/10.6084/m9.figshare.15079074).

**Table 5.** Number of differentially expressed genes

Comparison	Number of differentially expressed genes
ZnR *v.* Zn50	734
ZnR *v.* Zn100	439
Zn50 *v.* Zn100	340

Among the DEGs in ileum, we found that the expression of the Zn transporter, *ZIP4*, was greater (*P* < 0⋅0001) in the ZnR group when compared with the Zn100 group (Table 6). The expression of Zn transporter *ZIP10* was greater in the ZnR group compared with the Zn50 group (*P* = 0⋅012; Table 6). Furthermore, the pigs fed ZnR diet showed decreased expression of *MT-1A* (*P* = 0⋅008) and *MT-3* (*P* = 0⋅014) when compared with pigs fed Zn100 (Table 6).

**Table 6.** Expression changes of Zinc transporter genes

Gene name	Comparison	Log2 fold change	*P*-value
Zinc transporter *ZIP4*	ZnR *v*. Zn100	1⋅8504	
Zinc transporter *ZIP10*	ZnR *v.* Zn50	0⋅9282	0⋅0123
Metallothionein-1A	ZnR *v.* Zn100	−2⋅7387	0⋅0076
Metallothionein-3	ZnR *v.* Zn100	−2⋅4049	0⋅0135

^a^ZnR, (Zn-free vitamin–mineral mix + 0 mg of Zn/kg of diet); Zn50, (Zn-free vitamin–mineral mix + 50 mg of Zn/kg of diet); Zn100, (Zn-free vitamin–mineral mix + 100 mg of Zn/kg of diet).

**Fig. 2.**
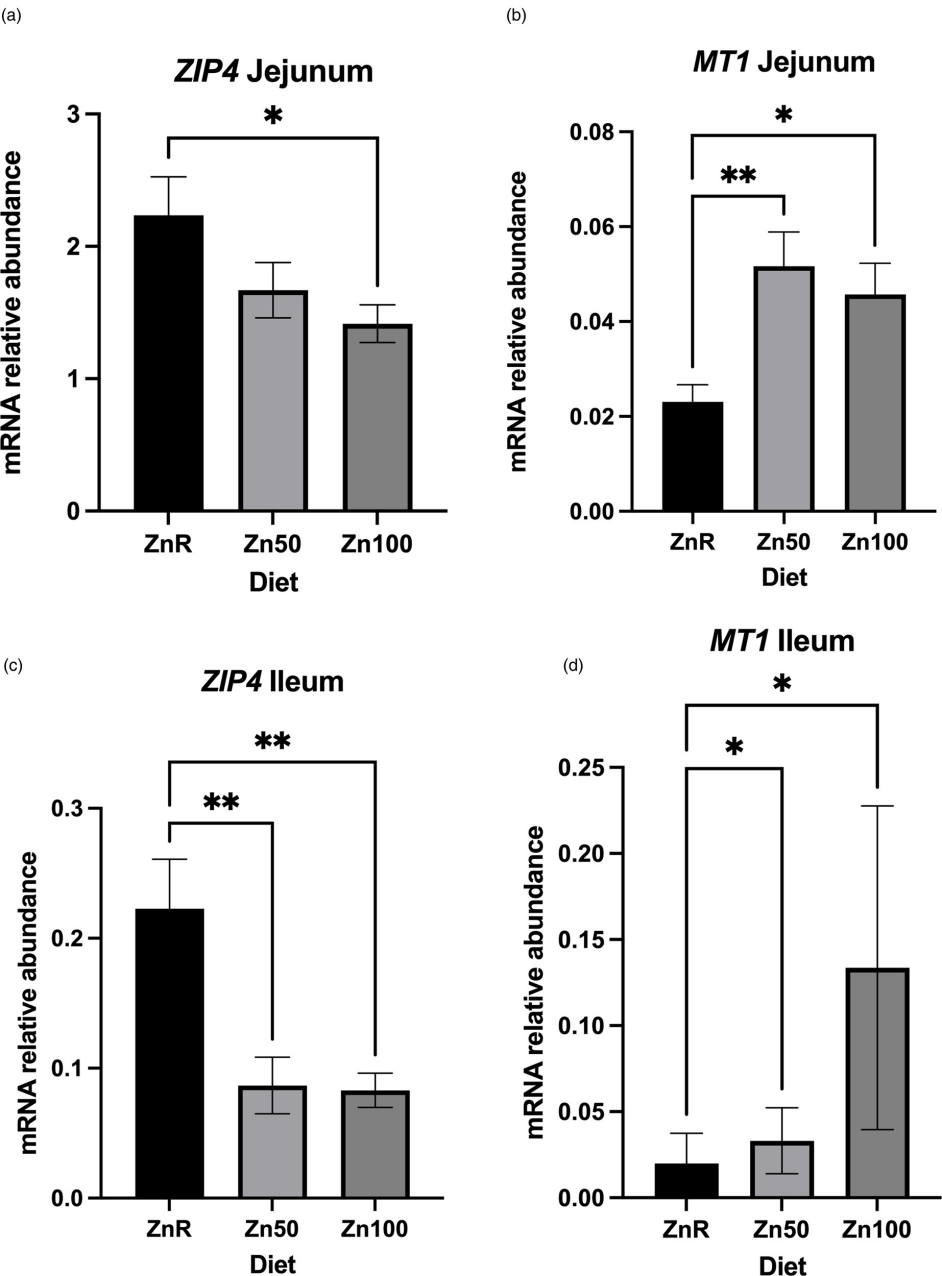
Gene expression in intestinal samples evaluated by qPCR. (a) ZIP4 and (b) MT1 expression in jejunum. (c) ZIP4 and (d) MT1 gene expression in ileum of pigs fed Zn-restricted diets (ZnR) or diets supplemented with 50 and 100 mg of Zn/kg of diet from ZnCl_2_ (Zn50 and Zn100, respectively) for 32 d. Data are presented as means ± sem. *P ≤ 0⋅05, **P ≤ 0⋅01. In jejunum samples, *n* 9 for all groups. In ileum samples, *n* 7 for ZnR, *n* 8 for Zn100 and Zn50.

In the jejunum, the expression of *ZIP4* was greater in the ZnR group compared with the Zn100 group (*P* = 0⋅019; Fig. 2(a)). The expression of *MT1* in jejunum was lower in the ZnR group compared with the Zn50 (*P* = 0⋅002) and Zn100 groups (*P* = 0⋅018; Fig. 2(b)).

qPCR analysis of ileal samples showed that the expression of *ZIP4* was greater in samples from the ZnR group compared with those from the Zn50 (*P* = 0⋅004) and Zn100 groups (*P* = 0⋅008; Fig. 2(c)). The expression of *MT1* detected by qPCR in the ileum of pigs in the ZnR group was lower than that of the Zn50 (*P* = 0⋅049) and Zn100 (*P* = 0⋅028) groups (Fig. 2(d)).

Network and pathway analysis of DEGs in ileum of pigs fed ZnR compared with Zn-supplemented diets revealed functional PANTHER categories related to transporter activities, binding proteins, catalytic activities, molecular function regulators, molecular transducer activities, structural molecular activities, transcription regulator activities and translation regulator activities (Supplementary Tables S4–S11; https://doi.org/10.6084/m9.figshare.15079074).

**Fig. 3.**
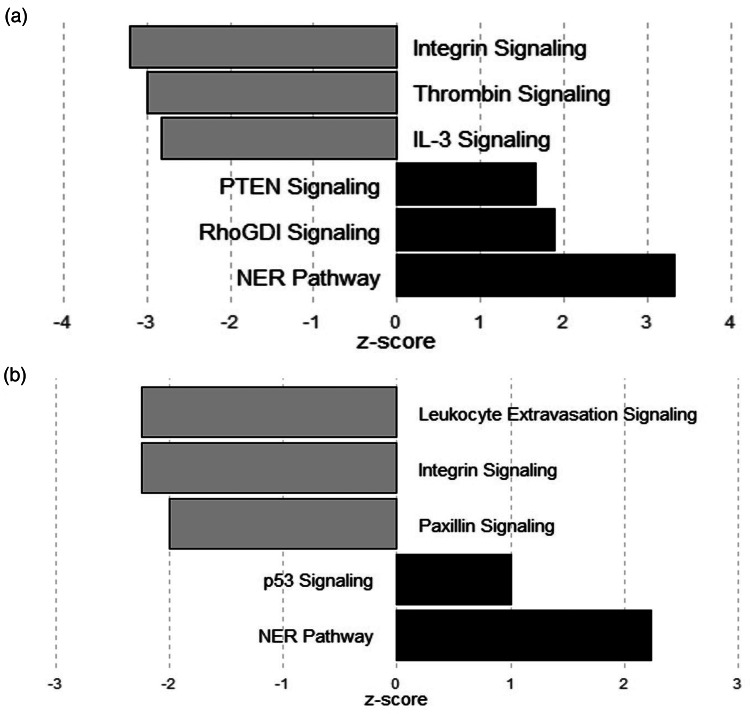
Top three up-regulated and down-regulated pathways identified through ingenuity pathway analysis of differentially expressed genes in ileum of pigs fed Zn-restricted diets (ZnR) compared with pigs fed diets supplemented with 50 and 100 mg of Zn/kg of diet from ZnCl_2_ (Zn50 (a) and Zn100 (b), respectively). *z*-score >2 or <−2 were considered significantly up-regulated (black bars) or down-regulated (grey bars), respectively. *n* 7 for ZnR, *n* 8 for Zn100 and Zn50.

The characterisation of the DEGs using IPA demonstrated that ZnR-fed pigs presented an up-regulation of genes related to nucleotide excision repair (NER) pathway when compared with pigs fed Zn-supplemented diets (Fig. 3). The top three down-regulated pathways (based on *z*-scores) in intestines of pigs fed ZnR diet compared with that of the Zn50 group included integrin, thrombin and interleukin-3 (IL-3) signalling (*P* ≤ 0⋅05; Fig. 3(a)). When intestines of ZnR diet-fed pigs were compared with those of Zn50 diet-fed pigs, the top three up-regulated pathways included phosphatase and tensin homolog (PTEN) signalling; Rho-specific guanine nucleotide dissociation inhibitor (RhoGDI) signalling and NER pathway (*P* ≤ 0⋅05; Fig. 3(a)). The top three down-regulated pathways in the ZnR group compared with the Zn100 group were related to leucocyte extravasation signalling, integrin and paxillin signalling (*P* ≤ 0⋅05; Fig. 3(b)). Genes belonging to NER pathway and p53 signalling were also up-regulated in pigs fed ZnR compared with those fed Zn100 (*P* ≤ 0⋅05).

**Fig. 4.**
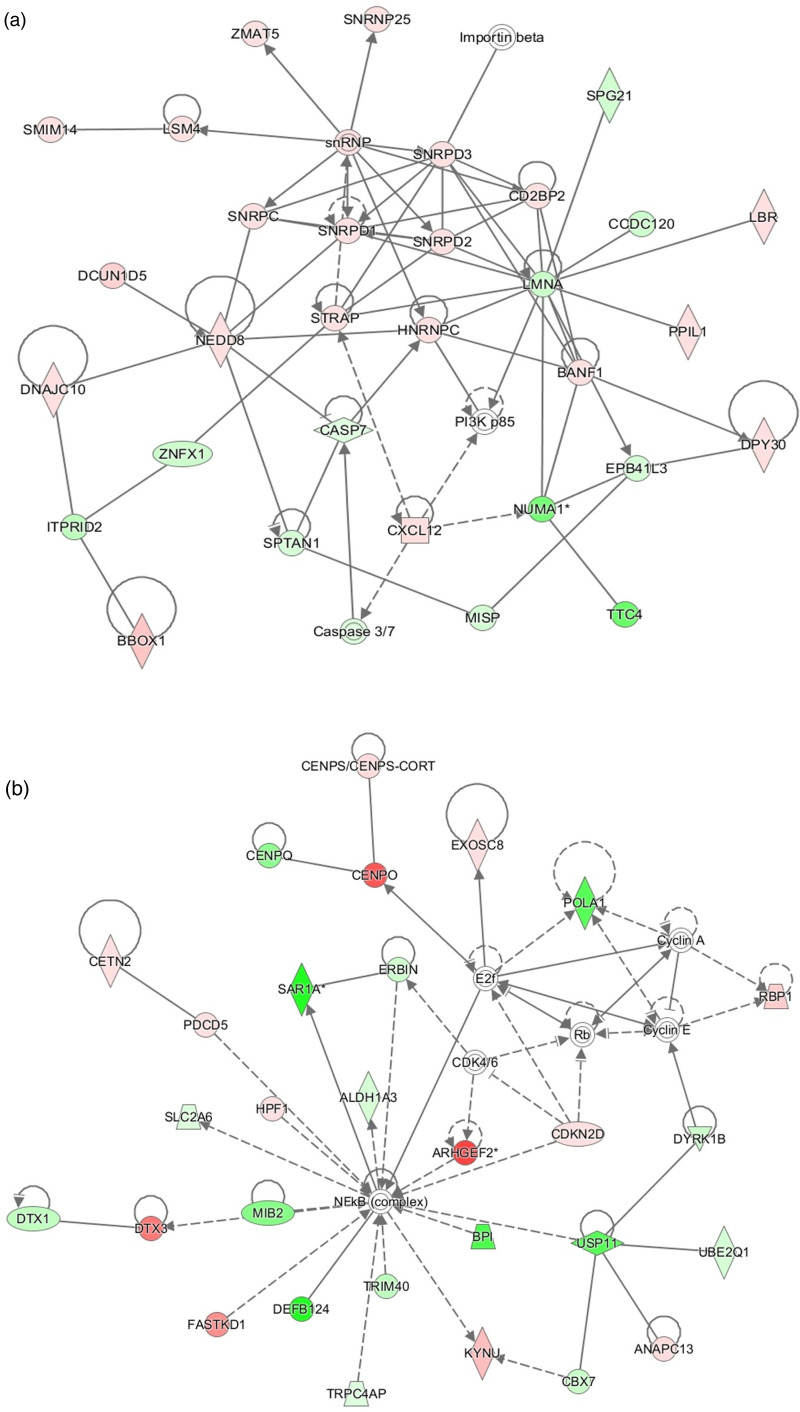
Top functional network identified in ileum of pigs fed zinc-restricted (ZnR) diet compared with those fed diets supplemented with (a) 50 mg of Zn/kg of diet from ZnCl_2_ (Zn50) includes functions related to cellular assembly and organisation, and cell morphology. When compared with those of pigs fed diet supplemented with (b) 100 mg of Zn/kg of diet from ZnCl_2_ (Zn100), top network includes functions related to lipid metabolism and drug metabolism. Genes are denoted as nodes. Node colour indicates up- (red) and down- (green) regulated genes. Edges (lines and arrows between nodes) represent direct (solid lines) and indirect (dashed lines) interactions between molecules as supported by information in the Ingenuity knowledge base. *n* 7 for ZnR, *n* 8 for Zn50 and Zn100.

The top network in intestines of pig fed ZnR diet compared with those on Zn50 diet was related to cellular assembly, organisation and cell morphology (*P* ≤ 0⋅05; Fig. 4(a)). Genes related to lipid metabolism, drug metabolism and small molecule biochemistry were observed as the top network (highest network score) when intestines of pigs fed ZnR diet were compared with pigs fed the Zn100 diet (*P* ≤ 0⋅05; Fig. 4(b)).

Compared with the Zn50 group, in the intestine of pigs in the Zn100 group, the top three down-regulated pathways were endothelin-1 signalling, relaxin signalling and the role of nuclear factor of activated T-cells (NFAT) in cardiac hypertrophy pathway, while the up-regulated pathways included PI3K signalling in B lymphocytes, leukocyte extravasation signalling and Th2 pathway (Supplementary Fig. S1; https://doi.org/10.6084/m9.figshare.15079074).

## Discussion

The main goal of the present study was to understand intestinal responses to nutritional Zn restriction and supplementation that could influence overall health of the animal and indicate early changes in response to Zn restriction.

In previous studies, signs of Zn deficiency such as reduced feed intake and parakeratosis were observed in pigs (3–4 weeks old) fed between 26 and 36 mg of Zn/kg of diet for 3 weeks^(24,42)^. A different study demonstrated that pigs fed diets deficient in Zn for 30 d showed signs of parakeratosis and serum Zn concentration of 0⋅297 ± 0⋅04 μg/ml^(43)^. In other studies, growing pigs (25 to 70 kg BW) supplemented with NRC-recommended levels of Zn between 50 and 150 mg of Zn/kg of diet for 16–120 d had serum Zn levels between 0⋅59 and 1⋅37 μg/ml^(43–45)^, which is close to the range of serum Zn levels of 0⋅66–1⋅2 μg/ml considered normal for humans. In the present study, we fed finishing pigs (about 77 kg BW) diets with and without Zn supplementation for 32 d. Although the Zn-restricted diet did not result in presentation of clinical signs of deficiency, serum Zn analysis showed that the pigs fed the ZnR diet had levels indicating Zn deficiency for pigs. These results support our aim of evaluating early responses of the small intestine to dietary Zn, before clinical signs of deficiency were observed.

Previous researchers have reported that low dietary intake of Zn increased transcription of *ZIP4* in intestinal enterocytes and hepatic cells of pigs^(46,47)^, in human blood cells^(48,49)^ and in mice^(50,51)^. Our observation of increased *ZIP4* expression in small intestines of ZnR-fed pigs is in accordance with those reports. Another Zn transporter *ZIP10*, involved in brain and liver Zn homeostasis and humoral immunity, was shown to be up-regulated in response to Zn restriction in mice^(^^52,53)^, which is comparable to the greater *ZIP10* expression observed in the ZnR-fed pigs in our study. These changes in the expression of Zn transporters in response to Zn restriction in the present study highlight the subclinical deficiency model in the absence of clinical signs that is susceptible to varying Zn levels and can be a useful tool to support human Zn nutrition. Furthermore, we observed *MT1* and *MT3* expression levels to be lower in the small intestine of ZnR-fed pigs compared with those in pigs fed Zn-supplemented diets. Studies in humans^(48,54)^ and other animals^(53,55,56)^ demonstrated *MT* expression is decreased under Zn deficiency. Taken together, gene expression changes detected by RNA-seq and confirmed by qPCR in the present study agreed with previous reports of Zn-deficient models.

Although jejunum is the site of most nutrient absorption, the pig ileum demonstrated greater differences in expression of genes associated with Zn deficiency. This observation is not unique, as other researchers reported^(36,57–60)^ that the pig ileum presents greater changes than the jejunum in response to different dietary interventions and infection.

In the present study, we identified signalling pathways and networks affected by dietary Zn restriction. Of note was the down-regulation of genes involved in integrin and ERK/MAPK signalling pathways and cellular organisation networks in pigs fed ZnR diet. These pathways are important for cell proliferation and differentiation and stress responses^(61)^. The findings in the present study agree with those of previous studies that demonstrated a role for Zn in activation of ERK and MAPK pathways in mast cells^(62)^, human neuroblastoma cells^(63)^, rat fibroblasts^(64)^ and pig intestine^(65)^. Furthermore, the up-regulation of genes related to NER and p53 signalling in ZnR-fed pigs may be associated with DNA damage and increased expression of DNA repair proteins that have previously been reported to be modulated by Zn^(66–69)^. The other up-regulated and down-regulated pathways observed in the intestine of pigs fed ZnR diet highlight the importance of Zn in immune functions and cellular processes including cellular organisation and repair.

Network analysis showed down-regulated genes linked to NFкB complex in ZnR compared with Zn100-fed pigs. NFкB is an important transcription factor that regulates immunity, inflammatory responses, cell proliferation and cell survival^(70)^. Zinc deficiency results in decreased NFкB activation and down-regulation of genes related to cell-mediated immunity, cellular development and cell death repression as reported in different studies^(54,71)^. Our findings of down-regulation of genes linked to NFκB suggest the small intestine of pigs fed ZnR diet became Zn-deficient. Taken together, our findings align with those of other Zn deficiency studies in humans and animals that have analysed a wide range of cells and organs. The results suggest that pigs can be a valuable model to evaluate and understand the intestinal immune status and health of pigs, and potentially humans, under Zn restriction.

The significance of varying Zn supplementation levels on physiological responses is not well understood. We studied dietary Zn levels known to satisfy growth requirements of pigs at a specific age and weight as indicated by the NRC (50 mg of Zn/kg of diet), and above their requirement (100 mg of Zn/kg of diet) to explore possible differential responses to Zn dose. The increase in expression of genes related to immune responses in the intestine of pigs fed 100 mg of Zn/kg of diet Zn compared with pigs fed 50 mg of Zn/kg of diet Zn provides an insight into the potential impact of Zn dose on maintaining health. The importance of Zn in maintaining immune function has been established previously, with Zn restriction resulting in impaired activity of immune cells including neutrophils, natural killer cells, macrophages and T lymphocytes^(4,17)^. Furthermore, although the mechanisms are not fully defined, Zn supplementation in pigs 1–2 weeks post-weaning improved innate and adaptive immune capacity by increasing phagocytosis, oxidative burst and T cell numbers^(72)^. Also, Zn supplementation is important for maintaining intestinal barrier integrity through regulation of various signalling pathways, albeit yet to be understood completely, as shown in weaned and growing pigs fed supplemental Zn^(65,73)^. Taken together, it is evident that Zn supplementation is important for maintaining health of pigs. Hence, Zn restriction may affect some of these immune related and stress response functions, as indicated by the results from the present study, even before clinical signs of Zn deficiency are observed. However, further research is needed to define those mechanisms and the optimal Zn dosage required to promote effective immune response, an issue relevant to pigs and humans. The findings from the present study may be pertinent to the early effects of Zn restriction on the health of humans as well.

In summary, analysis of pig's intestinal responses to dietary Zn restriction identified changes in gene expression, pathways and networks that mirrored changes observed in human Zn deficiency in different cells and organs, suggesting that the intestine could be a sentinel organ for Zn deficiency. Moreover, our results suggest that dose differences in Zn supplementation affect genes associated with immune responses. More research is necessary to define optimal dietary Zn supplementation that support health and stress responses, in addition to growth performance.

## Supplementary material

For supplementary material accompanying this paper visit https://doi.org/10.1017/jns.2022.105. click here to view supplementary material
