# Supplementary material for: Dietary zinc restriction affects the expression of genes related to immunity and stress response in the small intestine of pigs
Source: J Nutr Sci. 2022 Nov 22;11:e104. doi: 10.1017/jns.2022.105 (PMC9705703; doi:10.1017/jns.2022.105)
Supplement: Supplementary file 1 [file S2048679022001057sup001.docx]

**Supplementary Table 1. Growth performance of pigs in different treatment groups**

## **groups**

| Item | Diet | | | | |
| --- | --- | --- | --- | --- | --- |
|  | ZnR group | Zn50 | Zn100 | SEM | *p-value* |
| ADG, kg/d | 1.20 | 1.19 | 1.21 | 0.046 | 0.88 |
| ADFI, kg/d | 2.96 | 2.82 | 2.81 |  |  |

The results are least square means of nine pigs treated with the same diet. ZnR: (Zn-free vitamin-mineral mix + 0 ppm Zn), Zn50: (ZnR with 50 ppm Zn), Zn100: (ZnR with 100 ppm Zn). Within row, differing superscripts mean groups are different (*P* < 0.05). SEM means standard error. Feed intake was calculated on pen basis (n=1)

**Supplementary Table 2. Differentially expressed genes in ZnR vs Zn100 group**

| **Transcript ID** | **Gene description** | **Log2 fold change** | **p-value** |
| --- | --- | --- | --- |
| ENSSSCT00000050495.1 |  | -4.959847541 | 0.006281 |
| ENSSSCT00000033425.2 | defensin beta 124 | -4.34418554 | 0.01683 |
| ENSSSCT00000046895.1 |  | -4.00119839 | 0.027105 |
| ENSSSCT00000052136.1 |  | -3.839739336 | 0.006082 |
| ENSSSCT00000060529.1 | secretion associated Ras related GTPase 1A | -3.636068831 | 0.044898 |
| ENSSSCT00000003104.3 | casein kinase 2 alpha 2 | -3.274508404 | 0.040916 |
| ENSSSCT00000031664.2 | beta-galactosidase-1-like protein 2 | -3.165854647 | 0.01312 |
| ENSSSCT00000012833.3 | membrane metalloendopeptidase | -3.165087801 | 0.026973 |
| ENSSSCT00000045796.1 | methylsterol monooxygenase 1 | -3.110704204 | 0.025748 |
| ENSSSCT00000055308.1 | phosphatidylinositol glycan anchor biosynthesis class X | -3.056777516 | 0.015712 |
| ENSSSCT00000038131.1 | nuclear transcription factor Y subunit gamma | -3.021726663 | 0.005598 |
| ENSSSCT00000061268.1 |  | -3.001131536 | 0.00376 |
| ENSSSCT00000025172.2 | bactericidal permeability increasing protein | -2.897165528 | 0.006893 |
| ENSSSCT00000025430.2 | solute carrier family 40 member 1 | -2.87004 | 0.027607 |
| ENSSSCT00000003940.3 | non-compact myelin associated protein | -2.829076439 | 0.009955 |
| ENSSSCT00000044729.1 | somatostatin receptor 5 | -2.820079344 | 0.028132 |
| ENSSSCT00000027275.3 | derlin 1 | -2.810927803 | 0.036135 |
| ENSSSCT00000032296.2 | espin | -2.779493717 | 0.012982 |
| ENSSSCT00000059280.1 | spectrin alpha, non-erythrocytic 1 | -2.772848602 | 0.013341 |
| ENSSSCT00000034750.2 | ubiquitin specific peptidase 11 | -2.766177063 | 0.01166 |
| ENSSSCT00000058397.1 | metallothionein-1E | -2.738721164 | 0.00761 |
| ENSSSCT00000027952.2 | DNA polymerase alpha 1, catalytic subunit | -2.732384103 | 0.010957 |
| ENSSSCT00000041353.1 | C-C motif chemokine ligand 25 | -2.718854884 | 0.013288 |
| ENSSSCT00000049831.1 | biogenesis of lysosomal organelles complex 1 subunit 5 | -2.696871608 | 0.038297 |
| ENSSSCT00000065632.1 | pellino E3 ubiquitin protein ligase 1 | -2.690893832 | 0.041038 |
| ENSSSCT00000065513.1 | stromal cell derived factor 4 | -2.676677365 | 0.048595 |
| ENSSSCT00000047040.1 | solute carrier family 6 member 6 | -2.670172847 | 0.022058 |
| ENSSSCT00000026803.2 | multiple C2 and transmembrane domain containing 2 | -2.655087724 | 0.008045 |
| ENSSSCT00000049015.1 | actin beta | -2.65385096 | 0.000355 |
| ENSSSCT00000053029.1 | cyclin G associated kinase | -2.627434384 | 0.021667 |
| ENSSSCT00000053715.1 | short chain dehydrogenase/reductase family 42E, member 1 | -2.625788603 | 0.012844 |
| ENSSSCT00000029059.2 | metallothionein-2A | -2.60979197 | 0.004502 |
| ENSSSCT00000046086.1 | dystroglycan 1 | -2.607632658 | 0.023578 |
| ENSSSCT00000042921.1 | R3H domain containing 1 | -2.581450526 | 0.006693 |
| ENSSSCT00000050447.1 | NBR1, autophagy cargo receptor | -2.549613977 | 0.045288 |
| ENSSSCT00000033207.2 | cleavage and polyadenylation specific factor 1 | -2.503788558 | 0.047048 |
| ENSSSCT00000008875.3 | transmembrane protein 87B | -2.474409436 | 0.035388 |
| ENSSSCT00000013844.4 | ecto-NOX disulfide-thiol exchanger 2 | -2.466984785 | 0.035436 |
| ENSSSCT00000051414.1 | MIA SH3 domain ER export factor 2 | -2.462747164 | 0.033671 |
| ENSSSCT00000012586.3 | succinate-CoA ligase GDP-forming beta subunit | -2.446738915 | 0.035572 |
| ENSSSCT00000047481.1 | eukaryotic translation initiation factor 5 | -2.407949925 | 0.030621 |
| ENSSSCT00000027420.2 | metallothionein 3 | -2.404940256 | 0.013539 |
| ENSSSCT00000042792.1 | phosphatidylinositol transfer protein membrane associated 2 | -2.388990396 | 0.024475 |
| ENSSSCT00000061711.1 | nodal modulator 1 | -2.380185531 | 0.012249 |
| ENSSSCT00000045923.1 | LRR binding FLII interacting protein 1 | -2.311909843 | 0.023204 |
| ENSSSCT00000038661.1 | glycosyltransferase like domain containing 1 | -2.305638369 | 0.004944 |
| ENSSSCT00000052213.1 | arginyltransferase 1 | -2.26041364 | 0.020782 |
| ENSSSCT00000061972.1 | RB binding protein 4, chromatin remodeling factor | -2.251894955 | 0.021074 |
| ENSSSCT00000000273.3 | tensin 2 | -2.209104655 | 0.024811 |
| ENSSSCT00000065387.1 | erythrocyte membrane protein band 4.1 like 2 | -2.201902238 | 0.040849 |
| ENSSSCT00000041046.1 | YTH N6-methyladenosine RNA binding protein 3 | -2.198078878 | 0.018033 |
| ENSSSCT00000027940.2 | phosphodiesterase 3A | -2.183382641 | 0.031479 |
| ENSSSCT00000065743.1 | class II major histocompatibility complex transactivator | -2.135962022 | 0.012756 |
| ENSSSCT00000024470.2 | CD2 associated protein | -2.124849229 | 0.02017 |
| ENSSSCT00000048644.1 | zinc finger protein 709-like | -2.097953846 | 0.041639 |
| ENSSSCT00000052748.1 | TATA-box binding protein associated factor 1 | -2.09250734 | 0.0288 |
| ENSSSCT00000052849.1 | 5'-nucleotidase, cytosolic IIIA | -2.091490596 | 0.036506 |
| ENSSSCT00000049855.1 | solute carrier family 38 member 6 | -2.086625553 | 0.024279 |
| ENSSSCT00000008777.3 | lipase maturation factor 1 | -2.081905959 | 0.015583 |
| ENSSSCT00000044418.1 | lunapark, ER junction formation factor | -2.061795072 | 0.048055 |
| ENSSSCT00000022501.2 | claudin 15 | -2.01402558 | 0.047143 |
| ENSSSCT00000037570.1 | protein tyrosine phosphatase, receptor type F | -2.013513123 | 0.028394 |
| ENSSSCT00000053247.1 | CUGBP Elav-like family member 1 | -2.013369629 | 0.018986 |
| ENSSSCT00000045787.1 | Rho/Rac guanine nucleotide exchange factor 18 | -1.992268521 | 0.032995 |
| ENSSSCT00000018038.3 | adenosylhomocysteinase like 2 | -1.985704107 | 0.022895 |
| ENSSSCT00000034395.2 | activity dependent neuroprotector homeobox | -1.958978743 | 0.042089 |
| ENSSSCT00000047511.1 | mindbomb E3 ubiquitin protein ligase 2 | -1.948165924 | 0.01417 |
| ENSSSCT00000054510.1 | mannose phosphate isomerase | -1.908365016 | 0.03277 |
| ENSSSCT00000049484.1 | S100 calcium binding protein G | -1.902002763 | 0.045631 |
| ENSSSCT00000056966.1 |  | -1.8859117 | 0.035972 |
| ENSSSCT00000049430.1 |  | -1.877170938 | 0.007988 |
| ENSSSCT00000049180.1 | diacylglycerol O-acyltransferase 2 | -1.862106507 | 0.006609 |
| ENSSSCT00000037006.1 | NHS like 1 | -1.855386835 | 0.028826 |
| ENSSSCT00000055239.1 | spectrin repeat containing nuclear envelope family member 3 | -1.854423815 | 0.014432 |
| ENSSSCT00000062017.1 | mitogen-activated protein kinase kinase kinase 20 | -1.827180822 | 0.03964 |
| ENSSSCT00000053287.1 | MPV17, mitochondrial inner membrane protein | -1.821574231 | 0.036917 |
| ENSSSCT00000055525.1 | CCR4-NOT transcription complex subunit 2 | -1.811380101 | 0.003079 |
| ENSSSCT00000059967.1 | centromere protein Q | -1.811148978 | 0.010364 |
| ENSSSCT00000023051.2 | centrosomal protein 192 | -1.786200038 | 0.026431 |
| ENSSSCT00000063134.1 | stromal antigen 2 | -1.733735645 | 0.003195 |
| ENSSSCT00000018711.4 | ring finger protein 157 | -1.718648381 | 0.008557 |
| ENSSSCT00000055115.1 | megakaryocyte-associated tyrosine kinase | -1.688091718 | 0.033412 |
| ENSSSCT00000063685.1 | FAST kinase domains 3 | -1.66769599 | 0.048404 |
| ENSSSCT00000043702.1 |  | -1.655540683 | 0.042036 |
| ENSSSCT00000049323.1 | transgelin 2 | -1.585925284 | 0.00569 |
| ENSSSCT00000044550.1 | ribosomal protein L15 | -1.578272233 | 0.006544 |
| ENSSSCT00000000055.3 | alpha-N-acetylgalactosaminidase | -1.570927637 | 0.002291 |
| ENSSSCT00000052629.1 | slingshot protein phosphatase 1 | -1.568271926 | 0.038251 |
| ENSSSCT00000056069.1 | sphingosine-1-phosphate lyase 1 | -1.54932064 | 0.046176 |
| ENSSSCT00000018784.3 | ATP binding cassette subfamily A member 5 | -1.540679175 | 0.047314 |
| ENSSSCT00000018904.3 | glucose-6-phosphatase catalytic subunit 3 | -1.507942998 | 0.023439 |
| ENSSSCT00000017941.2 | T cell receptor beta variable 24-1 | -1.466967579 | 0.010056 |
| ENSSSCT00000060915.1 | neurogranin | -1.46140586 | 0.000181 |
| ENSSSCT00000042562.1 | fizzy and cell division cycle 20 related 1 | -1.442809573 | 0.030956 |
| ENSSSCT00000062609.1 | VPS39, HOPS complex subunit | -1.430649599 | 0.014503 |
| ENSSSCT00000044655.1 | DNA topoisomerase II binding protein 1 | -1.428008408 | 0.027284 |
| ENSSSCT00000048045.1 | phosphatidylinositol 3-kinase catalytic subunit type 3 | -1.410961716 | 0.048011 |
| ENSSSCT00000050802.1 | protein phosphatase 4 regulatory subunit 3B | -1.393202793 | 0.023583 |
| ENSSSCT00000029449.2 | nucleolar protein interacting with the FHA domain of MKI67 | -1.375101964 | 0.015669 |
| ENSSSCT00000059923.1 | F-box protein 25 | -1.373662914 | 0.023017 |
| ENSSSCT00000056107.1 | BTB domain and CNC homolog 1 | -1.351919866 | 0.048362 |
| ENSSSCT00000050670.1 | coenzyme Q8A | -1.344207653 | 0.010632 |
| ENSSSCT00000028943.2 | SAM and HD domain containing deoxynucleoside triphosphate triphosphohydrolase 1 | -1.319250768 | 0.040624 |
| ENSSSCT00000044709.1 | filamin B | -1.296541062 | 0.018409 |
| ENSSSCT00000034359.2 | CD27 molecule | -1.290576977 | 0.033284 |
| ENSSSCT00000061741.1 | CCR4-NOT transcription complex subunit 2 | -1.28316422 | 0.010584 |
| ENSSSCT00000037044.1 | SPT20 homolog, SAGA complex component | -1.282992888 | 0.034769 |
| ENSSSCT00000014394.3 | catenin delta 1 | -1.2828696 | 0.046185 |
| ENSSSCT00000040906.1 | crystallin lambda 1 | -1.248126297 | 0.033031 |
| ENSSSCT00000064959.1 | autophagy related 9A | -1.245194573 | 0.009526 |
| ENSSSCT00000064264.1 | mitochondrial transcription termination factor 1 | -1.226025339 | 0.02639 |
| ENSSSCT00000049618.1 | thyroid hormone receptor alpha | -1.223472333 | 0.045045 |
| ENSSSCT00000006135.3 | spermatid perinuclear RNA binding protein | -1.198374639 | 0.035193 |
| ENSSSCT00000041823.1 |  | -1.198030134 | 0.029429 |
| ENSSSCT00000053707.1 | jumonji domain containing 1C | -1.188381774 | 0.043804 |
| ENSSSCT00000058366.1 | histone H4 | -1.179909335 | 0.035792 |
| ENSSSCT00000058181.1 | PHD finger protein 6 | -1.178698537 | 0.040432 |
| ENSSSCT00000042723.1 |  | -1.156805729 | 0.042723 |
| ENSSSCT00000039888.1 | CD47 molecule | -1.152265688 | 0.034699 |
| ENSSSCT00000016405.3 | beta-carotene oxygenase 2 | -1.134426143 | 0.038252 |
| ENSSSCT00000040835.1 | NLR family pyrin domain containing 6 | -1.12820748 | 0.035143 |
| ENSSSCT00000048923.1 | SEC24 homolog A, COPII coat complex component | -1.115860791 | 0.046778 |
| ENSSSCT00000065484.1 | midkine | -1.104373474 | 0.038469 |
| ENSSSCT00000044106.1 |  | -1.101355982 | 0.002576 |
| ENSSSCT00000041061.1 | dual specificity tyrosine phosphorylation regulated kinase 1A | -1.100908355 | 0.010738 |
| ENSSSCT00000022913.2 | desmocollin 3 | -1.100869123 | 0.03478 |
| ENSSSCT00000062766.1 | ATPase phospholipid transporting 11B (putative) | -1.098524993 | 0.00962 |
| ENSSSCT00000055801.1 | HMG-box transcription factor 1 | -1.095762268 | 0.004856 |
| ENSSSCT00000035898.2 | interferon induced protein with tetratricopeptide repeats 2 | -1.09007487 | 0.043895 |
| ENSSSCT00000065498.1 | jumonji domain containing 1C | -1.083317255 | 0.04139 |
| ENSSSCT00000059743.1 | serine and arginine repetitive matrix 1 | -1.082724605 | 0.019058 |
| ENSSSCT00000001680.3 | TAP binding protein | -1.079311261 | 0.046639 |
| ENSSSCT00000050527.1 | nuclear factor, erythroid 2 like 1 | -1.076006792 | 0.009229 |
| ENSSSCT00000062485.1 | ADP ribosylation factor GTPase activating protein 2 | -1.073069871 | 0.017005 |
| ENSSSCT00000033688.2 | tripartite motif containing 40 | -1.071578592 | 0.031719 |
| ENSSSCT00000048678.1 | Mov10 RISC complex RNA helicase | -1.064998318 | 0.035351 |
| ENSSSCT00000038564.1 | transducin beta like 1 X-linked receptor 1 | -1.056351236 | 0.018321 |
| ENSSSCT00000050614.1 | TATA-box binding protein associated factor 9b | -1.049826426 | 0.027673 |
| ENSSSCT00000018608.3 | TNFAIP3 interacting protein 1 | -1.018549273 | 0.005738 |
| ENSSSCT00000063969.1 | chloride intracellular channel 5 | -1.015808734 | 0.033744 |
| ENSSSCT00000051274.1 | aminoadipate-semialdehyde dehydrogenase | -1.009699176 | 0.045428 |
| ENSSSCT00000064575.1 | myocardin related transcription factor A | -1.004472466 | 0.048227 |
| ENSSSCT00000058898.1 | structural maintenance of chromosomes 5 | -1.00266759 | 0.018533 |
| ENSSSCT00000045618.1 | deltex E3 ubiquitin ligase 1 | -0.999410408 | 0.028432 |
| ENSSSCT00000051842.1 |  | -0.984673497 | 0.025653 |
| ENSSSCT00000028505.2 | ALS2 C-terminal like | -0.983766222 | 0.022081 |
| ENSSSCT00000064542.1 | mitogen-activated protein kinase-activated protein kinase 3 | -0.957484259 | 0.015309 |
| ENSSSCT00000008841.3 | isoleucyl-tRNA synthetase | -0.932737304 | 0.023813 |
| ENSSSCT00000066361.1 | ribosomal protein L15 | -0.929361544 | 0.006013 |
| ENSSSCT00000008231.4 | VAMP associated protein B and C | -0.928260632 | 0.04673 |
| ENSSSCT00000045456.1 | apoptosis resistant E3 ubiquitin protein ligase 1 | -0.910840062 | 0.028985 |
| ENSSSCT00000043646.1 | TIA1 cytotoxic granule associated RNA binding protein | -0.904138189 | 0.033699 |
| ENSSSCT00000049858.1 | exportin 1 | -0.901053117 | 0.044963 |
| ENSSSCT00000000765.3 | vesicle associated membrane protein 1 | -0.900094818 | 0.025775 |
| ENSSSCT00000000096.3 | chromobox 7 | -0.897133718 | 0.047629 |
| ENSSSCT00000027578.2 | kinesin family member C2 | -0.876411376 | 0.042518 |
| ENSSSCT00000007121.2 | Rho/Rac guanine nucleotide exchange factor 2 | -0.872394127 | 0.042915 |
| ENSSSCT00000006423.2 | NOTCH regulated ankyrin repeat protein | -0.859657359 | 0.012769 |
| ENSSSCT00000024419.2 | dual specificity tyrosine phosphorylation regulated kinase 1B | -0.853913551 | 0.041478 |
| ENSSSCT00000039166.1 | putative MORF4 family-associated protein 1-like protein UPP | -0.837883812 | 0.033663 |
| ENSSSCT00000026311.2 |  | -0.824855449 | 0.023255 |
| ENSSSCT00000018462.3 | erbb2 interacting protein | -0.812067634 | 0.043856 |
| ENSSSCT00000044362.1 | ring finger protein 145 | -0.798189078 | 0.034922 |
| ENSSSCT00000018042.3 | transportin 3 | -0.793554249 | 0.022167 |
| ENSSSCT00000018503.3 | neuralized E3 ubiquitin protein ligase 1B | -0.790573861 | 0.028951 |
| ENSSSCT00000040688.1 | transducin like enhancer of split 4 | -0.775291699 | 0.039359 |
| ENSSSCT00000048866.1 | biogenesis of lysosomal organelles complex 1 subunit 3 | -0.76730163 | 0.024549 |
| ENSSSCT00000032022.2 | calcium/calmodulin dependent protein kinase II inhibitor 1 | -0.726065579 | 0.035377 |
| ENSSSCT00000016654.3 | phosphatidylinositol-4-phosphate 3-kinase catalytic subunit type 2 beta | -0.72425483 | 0.012029 |
| ENSSSCT00000005019.3 | SPG21, maspardin | -0.719184617 | 0.001356 |
| ENSSSCT00000013417.3 | ubiquitin like modifier activating enzyme 1 | -0.71291717 | 0.020728 |
| ENSSSCT00000008434.3 | heat shock protein family B (small) member 1 | -0.708477731 | 0.012669 |
| ENSSSCT00000003099.3 | CCR4-NOT transcription complex subunit 1 | -0.699306901 | 0.019303 |
| ENSSSCT00000025921.3 | La ribonucleoprotein domain family member 4 | -0.690812551 | 0.022057 |
| ENSSSCT00000054805.1 | ubiquitin conjugating enzyme E2 Q1 | -0.684371156 | 0.026486 |
| ENSSSCT00000009844.3 | cyclin I | -0.668591298 | 0.014265 |
| ENSSSCT00000063231.1 | 3'-phosphoadenosine 5'-phosphosulfate synthase 1 | -0.662306527 | 0.032571 |
| ENSSSCT00000028916.2 | aldehyde dehydrogenase 1 family member A3 | -0.65371195 | 0.043457 |
| ENSSSCT00000041445.1 | leukocyte receptor cluster member 8 | -0.630780943 | 0.02306 |
| ENSSSCT00000042890.1 | MAF bZIP transcription factor B | -0.63050233 | 0.043949 |
| ENSSSCT00000027501.2 | glucose-6-phosphate dehydrogenase | -0.609874869 | 0.025942 |
| ENSSSCT00000056660.1 | cell migration inducing hyaluronidase 2 | -0.606052148 | 0.020632 |
| ENSSSCT00000025107.2 | solute carrier family 2 member 6 | -0.582771794 | 0.009918 |
| ENSSSCT00000048286.1 | splicing factor 3b subunit 1 | -0.564411009 | 0.022422 |
| ENSSSCT00000058772.1 | interferon induced transmembrane protein 3 | -0.557555221 | 0.026134 |
| ENSSSCT00000008972.3 | zeta chain of T cell receptor associated protein kinase 70 | -0.552680922 | 0.043343 |
| ENSSSCT00000050223.1 | transmembrane protein 250 | -0.543039907 | 0.040971 |
| ENSSSCT00000001195.3 | cytidine monophosphate-N-acetylneuraminic acid hydroxylase | -0.53782996 | 0.046817 |
| ENSSSCT00000054928.1 | eukaryotic translation elongation factor 1 alpha 1 | -0.533389477 | 0.024299 |
| ENSSSCT00000052686.1 | RAB8B, member RAS oncogene family | -0.506583369 | 0.045799 |
| ENSSSCT00000007975.3 | transient receptor potential cation channel subfamily C member 4 associated protein | -0.481052109 | 0.029235 |
| ENSSSCT00000051483.1 | eukaryotic translation initiation factor 4A2 | -0.479625685 | 0.004749 |
| ENSSSCT00000039180.1 | cyclin L1 | -0.474265125 | 0.026064 |
| ENSSSCT00000045589.1 | methionine adenosyltransferase 2A | -0.470121326 | 0.007238 |
| ENSSSCT00000061170.1 | serine and arginine rich splicing factor 2 | -0.470020466 | 0.012797 |
| ENSSSCT00000003711.3 | dishevelled segment polarity protein 1 | -0.462308206 | 0.034469 |
| ENSSSCT00000036524.2 | tripartite motif containing 26 | -0.446875613 | 0.029094 |
| ENSSSCT00000053609.1 | nicotinate phosphoribosyltransferase | -0.429614594 | 0.017742 |
| ENSSSCT00000006644.4 | Kruppel like factor 10 | -0.414142956 | 0.023872 |
| ENSSSCT00000045547.1 | actin gamma 1 | -0.389199304 | 0.012686 |
| ENSSSCT00000014210.3 | calpain 1 | -0.381603332 | 0.038145 |
| ENSSSCT00000015317.3 | heterogeneous nuclear ribonucleoprotein H1 | -0.377943363 | 0.013967 |
| ENSSSCT00000045167.1 | toll interacting protein | -0.288153004 | 0.041529 |
| ENSSSCT00000017571.3 | activating transcription factor 1 | -0.279101127 | 0.046992 |
| ENSSSCT00000017493.3 | serine/threonine kinase 17b | -0.221740053 | 0.027007 |
| ENSSSCT00000018727.2 | histone H3.3 | 0.173381145 | 0.030872 |
| ENSSSCT00000045474.1 | ribosomal protein L10a | 0.19867978 | 0.036556 |
| ENSSSCT00000056853.1 | ATP synthase F1 subunit alpha | 0.199436812 | 0.01118 |
| ENSSSCT00000063010.1 | ribosomal protein L3 | 0.205333311 | 0.040185 |
| ENSSSCT00000008489.3 | coiled-coil-helix-coiled-coil-helix domain containing 2 | 0.211604455 | 0.034111 |
| ENSSSCT00000004081.3 | NADH:ubiquinone oxidoreductase core subunit V2 | 0.216740695 | 0.009177 |
| ENSSSCT00000062758.1 | adaptor related protein complex 3 subunit sigma 1 | 0.21704541 | 0.034464 |
| ENSSSCT00000005522.2 | ribosomal protein L36a like | 0.217449029 | 0.047606 |
| ENSSSCT00000016202.3 | signal peptidase complex subunit 2 | 0.218133438 | 0.008325 |
| ENSSSCT00000055147.1 | chloride intracellular channel 1 | 0.219545255 | 0.01512 |
| ENSSSCT00000043535.1 | splicing factor 3b subunit 5 | 0.22117522 | 0.024953 |
| ENSSSCT00000002569.3 | ERH, mRNA splicing and mitosis factor | 0.222739695 | 0.042866 |
| ENSSSCT00000011019.3 | small nuclear ribonucleoprotein D3 polypeptide | 0.228922044 | 0.028461 |
| ENSSSCT00000010797.3 | phosphatidylethanolamine binding protein 1 | 0.23451687 | 0.021202 |
| ENSSSCT00000031361.2 | chaperonin containing TCP1 subunit 6A | 0.237766152 | 0.035152 |
| ENSSSCT00000000992.3 |  | 0.237848486 | 0.021963 |
| ENSSSCT00000012657.3 | BRICK1, SCAR/WAVE actin nucleating complex subunit | 0.238793756 | 0.037091 |
| ENSSSCT00000002293.3 | defender against cell death 1 | 0.241591243 | 0.038201 |
| ENSSSCT00000010838.3 | endoplasmic reticulum protein 29 | 0.242732977 | 0.046689 |
| ENSSSCT00000053256.1 | ribosomal protein L6 | 0.244089601 | 0.046498 |
| ENSSSCT00000009878.3 | ribosomal protein S3A | 0.246005426 | 0.03767 |
| ENSSSCT00000005457.3 | ribosomal protein L4 | 0.246707226 | 0.025787 |
| ENSSSCT00000005698.3 | ribosomal protein S6 | 0.248510406 | 0.040151 |
| ENSSSCT00000000103.3 | translocase of outer mitochondrial membrane 22 | 0.249003738 | 0.02964 |
| ENSSSCT00000029514.2 | superoxide dismutase 1 | 0.249197011 | 0.042111 |
| ENSSSCT00000028465.2 | NDUFA4, mitochondrial complex associated | 0.255880655 | 0.006924 |
| ENSSSCT00000046881.1 | methyltransferase like 9 | 0.257664929 | 0.044694 |
| ENSSSCT00000004261.3 | mago homolog, exon junction complex subunit | 0.258312074 | 0.027693 |
| ENSSSCT00000027104.2 | hydroxysteroid 17-beta dehydrogenase 12 | 0.261464823 | 0.02027 |
| ENSSSCT00000027450.2 | RPL17-C18orf32 readthrough | 0.261904364 | 0.032592 |
| ENSSSCT00000059081.1 | mitochondrial ribosomal protein L9 | 0.261958565 | 0.023389 |
| ENSSSCT00000010191.3 | ribosomal protein L21 | 0.267017065 | 0.007866 |
| ENSSSCT00000014662.4 | complement factor D | 0.267768818 | 0.04247 |
| ENSSSCT00000057837.1 | DNA polymerase epsilon 4, accessory subunit | 0.271135272 | 0.036669 |
| ENSSSCT00000016835.3 | protein disulfide isomerase family A member 4 | 0.271367579 | 0.003512 |
| ENSSSCT00000022688.2 | COP9 signalosome subunit 9 | 0.27280752 | 0.042999 |
| ENSSSCT00000004049.3 | NADH:ubiquinone oxidoreductase subunit S5 | 0.274200688 | 0.007614 |
| ENSSSCT00000032340.2 | transmembrane protein 258 | 0.27447049 | 0.031281 |
| ENSSSCT00000006972.3 | ubiquitin-fold modifier conjugating enzyme 1 | 0.275495231 | 0.013822 |
| ENSSSCT00000010853.3 | coenzyme Q5, methyltransferase | 0.279427515 | 0.03362 |
| ENSSSCT00000032480.2 | NOP16 nucleolar protein | 0.280445211 | 0.035943 |
| ENSSSCT00000006146.3 | proteasome subunit beta 7 | 0.283098322 | 0.006509 |
| ENSSSCT00000010377.3 | ubiquitin C-terminal hydrolase L3 | 0.285938969 | 0.049511 |
| ENSSSCT00000044665.1 | complement C1q binding protein | 0.286182565 | 0.027148 |
| ENSSSCT00000023500.2 | proteasome subunit beta 1 | 0.286581949 | 0.021677 |
| ENSSSCT00000016710.3 |  | 0.28701156 | 0.007541 |
| ENSSSCT00000004329.4 | peroxiredoxin 1 | 0.289919638 | 0.007491 |
| ENSSSCT00000022560.2 | RNA polymerase II subunit G | 0.290626883 | 0.03328 |
| ENSSSCT00000001720.3 | ribosomal protein L10a | 0.294108106 | 0.027339 |
| ENSSSCT00000019150.3 | NME/NM23 nucleoside diphosphate kinase 2 | 0.295836307 | 0.024311 |
| ENSSSCT00000010647.3 | histone PARylation factor 1 | 0.296817717 | 0.02165 |
| ENSSSCT00000017506.3 | heat shock protein family E (Hsp10) member 1 | 0.297333862 | 0.023419 |
| ENSSSCT00000023057.2 | small nuclear ribonucleoprotein polypeptide E | 0.298119419 | 0.038805 |
| ENSSSCT00000010085.3 | PIGY upstream reading frame | 0.29868608 | 0.049873 |
| ENSSSCT00000050680.1 | thioredoxin domain containing 5 | 0.299790381 | 0.045529 |
| ENSSSCT00000010685.3 | RAN, member RAS oncogene family | 0.300075419 | 0.041262 |
| ENSSSCT00000060340.1 | diazepam binding inhibitor, acyl-CoA binding protein | 0.30391358 | 0.032429 |
| ENSSSCT00000016800.3 | peptidase, mitochondrial processing beta subunit | 0.305443926 | 0.021028 |
| ENSSSCT00000032799.2 | hydroxysteroid 17-beta dehydrogenase 10 | 0.307222775 | 0.029671 |
| ENSSSCT00000024316.2 | mitochondrial ribosomal protein L27 | 0.307831737 | 0.033246 |
| ENSSSCT00000022802.2 | ribosomal protein L11 | 0.309353768 | 0.032215 |
| ENSSSCT00000065434.1 | mitochondrial ribosomal protein L51 | 0.311562685 | 0.019545 |
| ENSSSCT00000014187.3 | ribonuclease H2 subunit C | 0.313870253 | 0.038174 |
| ENSSSCT00000000387.3 | SAP domain containing ribonucleoprotein | 0.314439271 | 0.032538 |
| ENSSSCT00000043326.1 | calnexin | 0.315530436 | 0.011648 |
| ENSSSCT00000005151.3 | sulfide quinone oxidoreductase | 0.319319703 | 0.031389 |
| ENSSSCT00000013014.3 | cytochrome c oxidase copper chaperone COX17 | 0.320361652 | 0.02367 |
| ENSSSCT00000007554.3 | ribosomal protein L5 | 0.322111032 | 0.030178 |
| ENSSSCT00000052062.1 | inosine triphosphatase | 0.327014594 | 0.01928 |
| ENSSSCT00000012129.3 | COMM domain containing 3 | 0.327817716 | 0.003654 |
| ENSSSCT00000046956.1 | peptidylprolyl isomerase B | 0.327995188 | 0.010633 |
| ENSSSCT00000024679.2 | transmembrane protein 70 | 0.328132662 | 0.043008 |
| ENSSSCT00000004100.3 | small nuclear ribonucleoprotein D1 polypeptide | 0.3294429 | 0.027299 |
| ENSSSCT00000000682.4 | mago homolog B, exon junction complex subunit | 0.329649969 | 0.031569 |
| ENSSSCT00000030693.2 | coiled-coil domain containing 58 | 0.332956538 | 0.02553 |
| ENSSSCT00000057144.1 | serine carboxypeptidase 1 | 0.334968074 | 0.028501 |
| ENSSSCT00000010255.3 | exosome component 8 | 0.335012764 | 0.02014 |
| ENSSSCT00000010764.3 | actin related protein 2/3 complex subunit 3 | 0.339457254 | 0.010263 |
| ENSSSCT00000065973.1 | RNA guanine-7 methyltransferase activating subunit | 0.340639035 | 0.009045 |
| ENSSSCT00000053097.1 | ubiquitin conjugating enzyme E2 E3 | 0.341174588 | 0.005343 |
| ENSSSCT00000000089.3 | ribosomal protein S19 binding protein 1 | 0.34628024 | 0.004864 |
| ENSSSCT00000032308.2 | general transcription factor IIH subunit 5 | 0.350660421 | 0.002173 |
| ENSSSCT00000003161.3 | programmed cell death 5 | 0.35165435 | 0.009166 |
| ENSSSCT00000004385.3 | peptidylprolyl isomerase H | 0.353007016 | 0.002455 |
| ENSSSCT00000047582.1 | translocase of outer mitochondrial membrane 5 | 0.353754923 | 0.029709 |
| ENSSSCT00000053892.1 | ADP ribosylation factor 1 | 0.359478332 | 0.041518 |
| ENSSSCT00000011734.3 | acyl-CoA dehydrogenase short/branched chain | 0.363629207 | 0.02584 |
| ENSSSCT00000061488.1 | ribosomal protein L22 like 1 | 0.36523233 | 0.013603 |
| ENSSSCT00000036496.2 | signal sequence receptor subunit 4 | 0.367066179 | 0.020022 |
| ENSSSCT00000007127.3 | death associated protein 3 | 0.367726349 | 0.02475 |
| ENSSSCT00000044885.1 | ribosomal protein L7a | 0.368356428 | 0.000605 |
| ENSSSCT00000057180.1 | ubiquitin C-terminal hydrolase L5 | 0.368596155 | 0.045443 |
| ENSSSCT00000008962.3 | mitochondrial ribosomal protein L30 | 0.373012698 | 0.009363 |
| ENSSSCT00000031595.2 | small nuclear ribonucleoprotein polypeptide G | 0.373214493 | 0.027029 |
| ENSSSCT00000026250.2 | RuvB like AAA ATPase 1 | 0.373786867 | 0.032105 |
| ENSSSCT00000019062.3 | ribosomal protein L19 | 0.378601268 | 0.001231 |
| ENSSSCT00000001988.3 | transmembrane p24 trafficking protein 3 | 0.381407531 | 0.048642 |
| ENSSSCT00000019502.2 | mediator complex subunit 11 | 0.384467939 | 0.046011 |
| ENSSSCT00000034332.2 | mitochondrial ribosomal protein L13 | 0.387269492 | 0.036567 |
| ENSSSCT00000043395.1 | ARP3 actin related protein 3 homolog | 0.387393344 | 0.007522 |
| ENSSSCT00000044779.1 | cyclin dependent kinase inhibitor 2D | 0.395881796 | 0.044184 |
| ENSSSCT00000001703.3 | ribosomal protein S10 | 0.395924265 | 0.024365 |
| ENSSSCT00000032639.2 | centrin 2 | 0.399654255 | 0.024788 |
| ENSSSCT00000016303.3 | TATA-box binding protein associated factor, RNA polymerase I subunit D | 0.407382841 | 0.023117 |
| ENSSSCT00000000979.3 | small nuclear ribonucleoprotein polypeptide F | 0.407643082 | 0.028674 |
| ENSSSCT00000031620.2 | SRA stem-loop interacting RNA binding protein | 0.409971216 | 0.000897 |
| ENSSSCT00000025596.2 | CDC28 protein kinase regulatory subunit 2 | 0.41489379 | 0.031626 |
| ENSSSCT00000024163.2 | transmembrane protein 219 | 0.417955268 | 0.038532 |
| ENSSSCT00000018160.3 | gamma-glutamylcyclotransferase | 0.420054802 | 0.048166 |
| ENSSSCT00000025222.2 | methyltransferase like 23 | 0.421253737 | 0.040443 |
| ENSSSCT00000039736.1 | nudix hydrolase 15 | 0.425841928 | 0.024805 |
| ENSSSCT00000039716.1 | C-X9-C motif containing 2 | 0.436516167 | 0.049382 |
| ENSSSCT00000013313.4 | peroxiredoxin 4 | 0.441920239 | 0.002211 |
| ENSSSCT00000055436.1 | interferon regulatory factor 1 | 0.445140956 | 0.035202 |
| ENSSSCT00000061063.1 | phospholipase A2 group VII | 0.446071099 | 0.009503 |
| ENSSSCT00000039336.1 | septin 7 | 0.451068003 | 0.028924 |
| ENSSSCT00000065535.1 | small integral membrane protein 4 | 0.452527047 | 0.010064 |
| ENSSSCT00000004969.3 | HAUS augmin like complex subunit 1 | 0.470246915 | 0.021998 |
| ENSSSCT00000057661.1 | anaphase promoting complex subunit 13 | 0.475829452 | 0.040307 |
| ENSSSCT00000050634.1 | ribosomal protein L15 | 0.476708591 | 0.003457 |
| ENSSSCT00000003776.3 | apoptosis-inducing, TAF9-like domain 1 | 0.488049582 | 0.041335 |
| ENSSSCT00000012916.3 | replication factor C subunit 4 | 0.490858453 | 0.040273 |
| ENSSSCT00000017118.3 | chromosome 2 open reading frame 76 | 0.492583192 | 0.009919 |
| ENSSSCT00000018479.3 | survival of motor neuron 1, telomeric | 0.497832996 | 0.012562 |
| ENSSSCT00000050490.1 | Rho GTPase activating protein 15 | 0.502342849 | 0.04661 |
| ENSSSCT00000045632.1 | citrate synthase | 0.512820408 | 0.005222 |
| ENSSSCT00000014614.3 | nucleobindin 2 | 0.526237768 | 0.031392 |
| ENSSSCT00000010449.3 | testis expressed 30 | 0.542625417 | 0.022171 |
| ENSSSCT00000057487.1 | ubiquitin like modifier activating enzyme 3 | 0.54935355 | 0.031473 |
| ENSSSCT00000041432.1 |  | 0.55409788 | 0.037237 |
| ENSSSCT00000035276.2 |  | 0.582099674 | 0.048943 |
| ENSSSCT00000014548.3 | inner mitochondrial membrane peptidase subunit 1 | 0.588183289 | 0.043731 |
| ENSSSCT00000029761.2 |  | 0.588821987 | 0.044998 |
| ENSSSCT00000060582.1 | linker for activation of T cells family member 2 | 0.604275661 | 0.000759 |
| ENSSSCT00000054278.1 | CD46 molecule, complement regulatory protein | 0.611940115 | 0.014212 |
| ENSSSCT00000062912.1 | polymeric immunoglobulin receptor | 0.618510944 | 0.032202 |
| ENSSSCT00000023171.2 | small nuclear ribonucleoprotein 13 | 0.643836339 | 0.046148 |
| ENSSSCT00000012769.3 | retinol binding protein 1 | 0.679180183 | 0.049154 |
| ENSSSCT00000058123.1 | pyrophosphatase (inorganic) 2 | 0.690840427 | 0.007289 |
| ENSSSCT00000065800.1 | serine peptidase inhibitor, Kazal type 1 | 0.691971939 | 0.009069 |
| ENSSSCT00000001841.3 | male-enhanced antigen 1 | 0.69573549 | 0.048002 |
| ENSSSCT00000027887.2 | ficolin (collagen/fibrinogen domain containing lectin) 2 (hucolin) | 0.720734643 | 0.037078 |
| ENSSSCT00000038540.1 |  | 0.723053182 | 0.034842 |
| ENSSSCT00000019521.3 | PHD finger protein 23 | 0.732001923 | 0.036861 |
| ENSSSCT00000006347.3 | haloacid dehalogenase like hydrolase domain containing 2 | 0.735266875 | 0.010278 |
| ENSSSCT00000060871.1 | joining chain of multimeric IgA and IgM | 0.778316406 | 0.008645 |
| ENSSSCT00000044867.1 | small nuclear ribonucleoprotein polypeptide E | 0.782231944 | 0.008183 |
| ENSSSCT00000043965.1 | defective in cullin neddylation 1 domain containing 5 | 0.795105705 | 0.014991 |
| ENSSSCT00000060061.1 | regenerating family member 4 | 0.802076206 | 0.045259 |
| ENSSSCT00000047803.1 | complement component 4 binding protein beta | 0.821263697 | 0.039087 |
| ENSSSCT00000058543.1 | ubiquinol-cytochrome c reductase complex assembly factor 2 | 0.821712855 | 0.005013 |
| ENSSSCT00000052266.1 | eukaryotic translation initiation factor 3 subunit J | 0.835091581 | 0.005904 |
| ENSSSCT00000057491.1 | joining chain of multimeric IgA and IgM | 0.867070446 | 0.014285 |
| ENSSSCT00000038394.1 | lactate dehydrogenase A | 0.869252302 | 9.40E-05 |
| ENSSSCT00000013703.2 | mortality factor 4 like 2 | 0.88149025 | 0.010777 |
| ENSSSCT00000037290.1 | kynureninase | 0.890615525 | 0.049768 |
| ENSSSCT00000025662.2 | NADH:ubiquinone oxidoreductase complex assembly factor 2 | 0.89369359 | 0.025578 |
| ENSSSCT00000025583.2 | SPG21, maspardin | 0.908714637 | 0.000539 |
| ENSSSCT00000054071.1 | 3'-phosphoadenosine 5'-phosphosulfate synthase 1 | 0.922063165 | 0.011105 |
| ENSSSCT00000043455.1 | anterior gradient 2, protein disulphide isomerase family member | 0.942333724 | 0.021638 |
| ENSSSCT00000010221.3 | high mobility group box 1 | 0.954766279 | 0.019115 |
| ENSSSCT00000017235.3 | ER lipid raft associated 2 | 0.964734721 | 0.031759 |
| ENSSSCT00000008435.3 | heat shock protein family B (small) member 1 | 1.01211983 | 0.010174 |
| ENSSSCT00000026717.2 | anterior gradient 2, protein disulphide isomerase family member | 1.015670694 | 0.020023 |
| ENSSSCT00000054064.1 |  | 1.034074494 | 0.041967 |
| ENSSSCT00000033582.2 | joining chain of multimeric IgA and IgM | 1.034242492 | 0.008713 |
| ENSSSCT00000046103.1 | MPV17, mitochondrial inner membrane protein | 1.037899842 | 0.040713 |
| ENSSSCT00000048246.1 | FA complementation group D2 | 1.03846384 | 0.034106 |
| ENSSSCT00000045975.1 | Yip1 domain family member 1 | 1.056597714 | 0.035982 |
| ENSSSCT00000048751.1 | secretion associated Ras related GTPase 1A | 1.057429994 | 0.016004 |
| ENSSSCT00000000461.3 | signal transducer and activator of transcription 6 | 1.082710032 | 0.020184 |
| ENSSSCT00000029571.2 | S100P binding protein | 1.088169238 | 0.035415 |
| ENSSSCT00000035298.2 | neurocalcin delta | 1.115550615 | 0.033728 |
| ENSSSCT00000007022.4 | mucosal pentraxin | 1.139076358 | 0.039897 |
| ENSSSCT00000054543.1 | Ras association domain family member 4 | 1.141947863 | 0.034512 |
| ENSSSCT00000050456.1 | FK506 binding protein 5 | 1.143163451 | 0.039705 |
| ENSSSCT00000013161.3 | BTB domain and CNC homolog 1 | 1.14494564 | 0.021799 |
| ENSSSCT00000030321.2 | transmembrane protein 17 | 1.15067477 | 0.02333 |
| ENSSSCT00000058777.1 | F-box protein 25 | 1.150716986 | 0.006763 |
| ENSSSCT00000007258.3 | selenium binding protein 1 | 1.156486293 | 0.039847 |
| ENSSSCT00000056790.1 | high mobility group nucleosomal binding domain 2 | 1.292765253 | 0.031326 |
| ENSSSCT00000033942.2 | pyruvate dehydrogenase kinase 3 | 1.322913398 | 0.018664 |
| ENSSSCT00000053543.1 | C-C motif chemokine receptor like 2 | 1.374484007 | 0.007695 |
| ENSSSCT00000062488.1 | PMS1 homolog 1, mismatch repair system component | 1.417263434 | 0.014721 |
| ENSSSCT00000043331.1 | TATA-box binding protein associated factor 9 | 1.420153287 | 0.001749 |
| ENSSSCT00000016571.3 | hepatic and glial cell adhesion molecule | 1.46303621 | 0.013041 |
| ENSSSCT00000042803.1 | CCR4-NOT transcription complex subunit 7 | 1.504271921 | 0.011428 |
| ENSSSCT00000038522.1 | FAST kinase domains 1 | 1.54622454 | 0.049988 |
| ENSSSCT00000042456.1 | NIMA related kinase 4 | 1.605599713 | 0.03639 |
| ENSSSCT00000029033.2 | synaptotagmin 12 | 1.609146873 | 0.020244 |
| ENSSSCT00000040544.1 |  | 1.612415409 | 0.048237 |
| ENSSSCT00000064664.1 | Sus scrofa histone cluster 2, H3-like (LOC100156741), mRNA. | 1.660775139 | 0.025202 |
| ENSSSCT00000052707.1 |  | 1.679979564 | 0.023987 |
| ENSSSCT00000051608.1 |  | 1.761136345 | 4.18E-05 |
| ENSSSCT00000051715.1 | zinc finger and SCAN domain containing 26 | 1.788238348 | 0.030626 |
| ENSSSCT00000061608.1 | 5'-nucleotidase, cytosolic II | 1.806814307 | 0.014073 |
| ENSSSCT00000017304.3 | proteasome 26S subunit, non-ATPase 14 | 1.839223564 | 0.006506 |
| ENSSSCT00000006485.3 | solute carrier family 39 member 4 | 1.850384811 | 7.23E-11 |
| ENSSSCT00000029673.2 | deltex E3 ubiquitin ligase 3 | 1.854547428 | 0.035272 |
| ENSSSCT00000037156.1 | ArfGAP with GTPase domain, ankyrin repeat and PH domain 3 | 1.87467453 | 0.040468 |
| ENSSSCT00000051655.1 |  | 1.946438394 | 0.042945 |
| ENSSSCT00000012196.3 | tubulin alpha-1C chain | 1.95922724 | 0.001706 |
| ENSSSCT00000045699.1 | myocyte enhancer factor 2C | 1.973800743 | 0.049942 |
| ENSSSCT00000044466.1 | thiosulfate sulfurtransferase | 2.028433382 | 0.044823 |
| ENSSSCT00000022990.2 | histone cluster 1 H2A family member b | 2.030857201 | 0.01944 |
| ENSSSCT00000011268.3 | ecdysoneless cell cycle regulator | 2.047624347 | 0.018739 |
| ENSSSCT00000005790.3 | structural maintenance of chromosomes 5 | 2.062323704 | 0.017623 |
| ENSSSCT00000040669.1 | isoleucyl-tRNA synthetase | 2.067280971 | 0.004132 |
| ENSSSCT00000027869.2 | solute carrier family 2 member 3 | 2.067870038 | 0.036092 |
| ENSSSCT00000013089.3 | adhesion G protein-coupled receptor G7 | 2.116645219 | 0.039493 |
| ENSSSCT00000008482.3 | tyrosylprotein sulfotransferase 1 | 2.123213283 | 0.000277 |
| ENSSSCT00000010388.3 | SLAIN motif family member 1 | 2.129199763 | 0.047729 |
| ENSSSCT00000035625.2 | zeta chain of T cell receptor associated protein kinase 70 | 2.250826767 | 0.025172 |
| ENSSSCT00000052291.1 | centromere protein O | 2.312171767 | 0.009337 |
| ENSSSCT00000018609.3 | TNFAIP3 interacting protein 1 | 2.363813142 | 0.040576 |
| ENSSSCT00000010533.3 | nucleophosmin/nucleoplasmin 2 | 2.376955697 | 0.046201 |
| ENSSSCT00000047161.1 | transducin beta like 1 X-linked receptor 1 | 2.404918728 | 0.046924 |
| ENSSSCT00000049710.1 | stromal antigen 2 | 2.434957328 | 0.007216 |
| ENSSSCT00000051803.1 | nucleophosmin/nucleoplasmin 2 | 2.453677435 | 0.005928 |
| ENSSSCT00000011133.2 | microtubule associated protein 10 | 2.512323405 | 0.011556 |
| ENSSSCT00000002306.2 |  | 2.558005317 | 0.034414 |
| ENSSSCT00000047493.1 | nodal modulator 1 | 2.562758719 | 0.013032 |
| ENSSSCT00000045907.1 | Rho/Rac guanine nucleotide exchange factor 2 | 2.569667166 | 0.002398 |
| ENSSSCT00000033983.2 | integrator complex subunit 6 like | 2.660260734 | 0.031047 |
| ENSSSCT00000050656.1 | kizuna centrosomal protein | 2.713268324 | 0.006817 |
| ENSSSCT00000004585.3 | NHS like 1 | 2.71432107 | 0.004883 |
| ENSSSCT00000040997.1 | myotubularin related protein 8 | 2.798682571 | 0.040743 |
| ENSSSCT00000053777.1 | ATPase phospholipid transporting 11B (putative) | 3.126751537 | 0.000695 |
| ENSSSCT00000057769.1 | erythrocyte membrane protein band 4.1 | 3.31805943 | 0.033792 |
| ENSSSCT00000042090.1 | caspase 3 | 3.429768767 | 0.023051 |
| ENSSSCT00000063674.1 |  | 3.582010794 | 0.000652 |
| ENSSSCT00000047282.1 | dystrobrevin binding protein 1 | 4.040666712 | 0.002075 |
| ENSSSCT00000030537.2 | histone H4 | 4.382342749 | 0.006134 |

**Supplementary Table 3. Differentially expressed genes in ZnR vs Zn50 group**

| **Transcript ID** | **Gene description** | **Log2 fold change** | **p-value** |
| --- | --- | --- | --- |
| ENSSSCT00000043442.1 |  | -4.918175607 | 0.008407 |
| ENSSSCT00000050495.1 |  | -4.546406138 | 0.04725 |
| ENSSSCT00000052136.1 |  | -4.29777293 | 0.018966 |
| ENSSSCT00000047688.1 | ring finger and FYVE like domain containing E3 ubiquitin protein ligase | -4.271044671 | 0.000528 |
| ENSSSCT00000010412.3 | multidrug resistance-associated protein 4-like | -4.099744509 | 0.022694 |
| ENSSSCT00000057667.1 | apelin | -3.738256665 | 0.012467 |
| ENSSSCT00000046592.1 | ring finger protein 144A | -3.629022614 | 0.04807 |
| ENSSSCT00000014068.3 | fibroblast growth factor 19 | -3.333978257 | 0.045846 |
| ENSSSCT00000048328.1 | cytoplasmic linker associated protein 2 | -3.213370089 | 0.008653 |
| ENSSSCT00000044646.1 | TATA-box binding protein associated factor, RNA polymerase I subunit A | -3.086108327 | 0.003658 |
| ENSSSCT00000065381.1 | tetraspanin 15 | -2.709571642 | 0.009534 |
| ENSSSCT00000043400.1 | spermatogenesis associated 2 | -2.616794064 | 0.011347 |
| ENSSSCT00000026077.2 | nuclear receptor subfamily 5 group A member 2 | -2.597392193 | 0.033351 |
| ENSSSCT00000024467.2 | lithostathine-like | -2.568243266 | 0.00616 |
| ENSSSCT00000062411.1 |  | -2.467834786 | 0.026013 |
| ENSSSCT00000023398.2 | 5'-nucleotidase domain containing 3 | -2.376464724 | 0.02181 |
| ENSSSCT00000050440.1 | microsomal glutathione S-transferase 3 | -2.367373167 | 0.04103 |
| ENSSSCT00000024858.2 | ring finger protein 183 | -2.317166302 | 0.019668 |
| ENSSSCT00000041110.1 | tetratricopeptide repeat domain 4 | -2.235860609 | 0.02409 |
| ENSSSCT00000056963.1 | SH2 domain-containing protein 4A | -2.187375335 | 0.040908 |
| ENSSSCT00000032897.2 | GRB2 associated binding protein 1 | -2.171666798 | 0.01516 |
| ENSSSCT00000045257.1 | ferric chelate reductase 1 | -2.17084954 | 0.022864 |
| ENSSSCT00000040117.1 | N(alpha)-acetyltransferase 16, NatA auxiliary subunit | -2.148664827 | 0.044048 |
| ENSSSCT00000040401.1 | BCL2 interacting protein 3 like | -2.132583546 | 0.034007 |
| ENSSSCT00000058188.1 | SLC9A3 regulator 1 | -2.129673334 | 0.007243 |
| ENSSSCT00000049539.1 | calcium-activated chloride channel regulator 4 | -2.11200537 | 0.000345 |
| ENSSSCT00000059414.1 | retinoic acid induced 1 | -2.083285704 | 0.018009 |
| ENSSSCT00000052082.1 | nuclear mitotic apparatus protein 1 | -2.070125276 | 0.029195 |
| ENSSSCT00000000278.3 | cysteine sulfinic acid decarboxylase | -2.068727098 | 0.044438 |
| ENSSSCT00000055239.1 | spectrin repeat containing nuclear envelope family member 3 | -1.977241986 | 0.039233 |
| ENSSSCT00000054703.1 | family with sequence similarity 49 member B | -1.893251911 | 0.01414 |
| ENSSSCT00000066043.1 | serine/threonine kinase 40 | -1.876798512 | 0.047707 |
| ENSSSCT00000025381.2 | trinucleotide repeat containing 18 | -1.764603113 | 0.01538 |
| ENSSSCT00000035898.2 | interferon induced protein with tetratricopeptide repeats 2 | -1.732722857 | 0.012366 |
| ENSSSCT00000014039.3 | solute carrier family 25 member 22 | -1.726628037 | 0.032333 |
| ENSSSCT00000050410.1 |  | -1.711040076 | 0.039031 |
| ENSSSCT00000005664.2 | interferon epsilon | -1.64564184 | 0.022883 |
| ENSSSCT00000054807.1 |  | -1.596338544 | 0.028895 |
| ENSSSCT00000002914.3 | glutathione S-transferase A1-like | -1.579176642 | 0.015914 |
| ENSSSCT00000045665.1 | glutathione S-transferase Mu 1-like | -1.567449795 | 0.020704 |
| ENSSSCT00000018711.4 | ring finger protein 157 | -1.560303115 | 0.0209 |
| ENSSSCT00000003924.3 | leucine zipper protein 1 | -1.502432179 | 0.008732 |
| ENSSSCT00000060915.1 | neurogranin | -1.479715989 | 0.025992 |
| ENSSSCT00000036127.2 | zinc finger DHHC-type containing 9 | -1.470741632 | 0.02155 |
| ENSSSCT00000059095.1 | nuclear mitotic apparatus protein 1 | -1.456694194 | 0.008749 |
| ENSSSCT00000026311.2 |  | -1.453464716 | 8.89E-05 |
| ENSSSCT00000022686.2 | cytochrome P450 C42 | -1.433946664 | 0.011878 |
| ENSSSCT00000051531.1 | multidrug resistance-associated protein 4 | -1.425918064 | 0.015571 |
| ENSSSCT00000049995.1 |  | -1.420559334 | 0.048885 |
| ENSSSCT00000045395.1 | PDZ domain containing 3 | -1.419118179 | 0.037456 |
| ENSSSCT00000045468.1 | globoside alpha-1,3-N-acetylgalactosaminyltransferase 1 (FORS blood group) | -1.393500723 | 0.004543 |
| ENSSSCT00000017941.2 | T cell receptor beta variable 24-1 | -1.390483238 | 0.029619 |
| ENSSSCT00000049323.1 | transgelin 2 | -1.387194054 | 0.039512 |
| ENSSSCT00000016117.2 |  | -1.35052816 | 0.018349 |
| ENSSSCT00000040835.1 | NLR family pyrin domain containing 6 | -1.308831922 | 0.021979 |
| ENSSSCT00000032274.2 | vitamin D receptor | -1.261165791 | 0.00282 |
| ENSSSCT00000059410.1 | calcium-activated chloride channel regulator 4 | -1.249413839 | 0.01102 |
| ENSSSCT00000057148.1 | CXADR, Ig-like cell adhesion molecule | -1.222053283 | 0.045368 |
| ENSSSCT00000025573.2 | otopetrin 2 | -1.211675718 | 0.034793 |
| ENSSSCT00000058772.1 | interferon induced transmembrane protein 3 | -1.177198149 | 5.50E-06 |
| ENSSSCT00000048370.1 | DExD/H-box helicase 58 | -1.175037537 | 0.02822 |
| ENSSSCT00000063969.1 | chloride intracellular channel 5 | -1.166453694 | 0.029951 |
| ENSSSCT00000007130.3 | gon-4 like | -1.160545812 | 0.034371 |
| ENSSSCT00000049672.1 | fructose-bisphosphatase 2 | -1.122310761 | 0.033471 |
| ENSSSCT00000052191.1 | aldo-keto reductase family 1 member E2 | -1.105062795 | 0.042983 |
| ENSSSCT00000016214.3 | monoacylglycerol O-acyltransferase 2 | -1.10372877 | 0.017537 |
| ENSSSCT00000008948.3 | neuronal PAS domain protein 2 | -1.097203291 | 0.041855 |
| ENSSSCT00000007594.3 | calcium-activated chloride channel regulator 4 | -1.076925196 | 0.010371 |
| ENSSSCT00000059442.1 | transmembrane protein 56 | -1.076572852 | 0.027061 |
| ENSSSCT00000014049.2 | interferon regulatory factor 7 | -1.065021038 | 0.006292 |
| ENSSSCT00000002803.3 | amnion associated transmembrane protein | -1.052927911 | 0.007684 |
| ENSSSCT00000036933.1 | FRAT2, WNT signaling pathway regulator | -1.036322147 | 0.010909 |
| ENSSSCT00000053274.1 | tropomyosin 2 | -1.028343852 | 0.020883 |
| ENSSSCT00000048297.1 | leucine rich repeat containing 45 | -1.021512382 | 0.013375 |
| ENSSSCT00000022953.2 | transient receptor potential cation channel subfamily M member 4 | -1.010630841 | 0.016555 |
| ENSSSCT00000053135.1 | ATPase phospholipid transporting 10B (putative) | -1.009019898 | 0.017275 |
| ENSSSCT00000039842.1 | DExD/H-box helicase 60 | -1.008364726 | 0.034798 |
| ENSSSCT00000000113.3 | casein kinase I | -0.994473295 | 0.032338 |
| ENSSSCT00000056735.1 | ITPR interacting domain containing 2 | -0.991529893 | 0.029132 |
| ENSSSCT00000038811.1 | multidrug resistance-associated protein 4-like | -0.990538914 | 0.029443 |
| ENSSSCT00000006423.2 | NOTCH regulated ankyrin repeat protein | -0.989861817 | 0.042938 |
| ENSSSCT00000065647.1 | plexin B2 | -0.982283993 | 0.006337 |
| ENSSSCT00000024050.2 | creatine kinase B | -0.980943823 | 0.019224 |
| ENSSSCT00000038172.1 | centrosomal protein 170B | -0.978875295 | 0.033572 |
| ENSSSCT00000018503.3 | neuralized E3 ubiquitin protein ligase 1B | -0.978437971 | 0.022814 |
| ENSSSCT00000056748.1 | galectin 9 | -0.971133618 | 0.005837 |
| ENSSSCT00000055212.1 | MX dynamin like GTPase 2 | -0.951192225 | 0.029013 |
| ENSSSCT00000019019.3 | tensin 4 | -0.949448912 | 0.006941 |
| ENSSSCT00000022755.2 | zinc finger protein 276 | -0.940982525 | 0.043309 |
| ENSSSCT00000040690.1 | BCL2 like 1 | -0.935541396 | 0.033058 |
| ENSSSCT00000031440.2 | RAS like proto-oncogene B | -0.912153427 | 0.047481 |
| ENSSSCT00000065180.1 | nitric oxide synthase 2 | -0.898817244 | 0.026766 |
| ENSSSCT00000014281.3 | asparaginase like 1 | -0.89676788 | 0.001576 |
| ENSSSCT00000024363.2 | ovo like transcriptional repressor 1 | -0.895877294 | 0.013146 |
| ENSSSCT00000013832.3 | X-prolyl aminopeptidase 2 | -0.895812244 | 0.000433 |
| ENSSSCT00000014566.3 | solute carrier family 5 member 12 | -0.890375855 | 0.030912 |
| ENSSSCT00000063809.1 | glycerol-3-phosphate dehydrogenase 2 | -0.889416369 | 0.049165 |
| ENSSSCT00000003961.3 | keratinocyte differentiation factor 1 | -0.88911673 | 0.014211 |
| ENSSSCT00000055801.1 | HMG-box transcription factor 1 | -0.885867094 | 0.036312 |
| ENSSSCT00000062425.1 | apolipoprotein B | -0.882777072 | 0.031226 |
| ENSSSCT00000011953.3 | E74 like ETS transcription factor 3 | -0.881208134 | 0.000357 |
| ENSSSCT00000042890.1 | MAF bZIP transcription factor B | -0.878498045 | 0.00225 |
| ENSSSCT00000002141.3 | promyelocytic leukemia | -0.876577668 | 0.022528 |
| ENSSSCT00000054884.1 | hepatocyte nuclear factor 4 gamma | -0.874026864 | 0.02513 |
| ENSSSCT00000053084.1 | calpastatin | -0.870541997 | 0.035201 |
| ENSSSCT00000060129.1 | interferon alpha inducible protein 6 | -0.866987302 | 0.040002 |
| ENSSSCT00000007585.3 | guanylate binding protein 1, interferon-inducible | -0.859225488 | 0.044972 |
| ENSSSCT00000007531.3 | sorting nexin 7 | -0.85743586 | 0.025935 |
| ENSSSCT00000052285.1 | retinoid X receptor alpha | -0.852749897 | 0.018997 |
| ENSSSCT00000050048.1 | MOB kinase activator 3B | -0.850762468 | 0.034864 |
| ENSSSCT00000033088.2 | membrane palmitoylated protein 1 | -0.843114521 | 0.01358 |
| ENSSSCT00000019522.3 | Y-box binding protein 2 | -0.839455276 | 0.048411 |
| ENSSSCT00000040647.1 | sphingomyelin phosphodiesterase acid like 3B | -0.831026762 | 0.009572 |
| ENSSSCT00000040790.1 | BTB domain containing 6 | -0.830478521 | 0.009208 |
| ENSSSCT00000019093.3 | homeobox B6 | -0.824646943 | 0.025669 |
| ENSSSCT00000058447.1 | cytochrome P450, family 4, subfamily F, polypeptide 2 | -0.821346085 | 0.038418 |
| ENSSSCT00000006664.3 | odd-skipped related transciption factor 2 | -0.819956046 | 0.01079 |
| ENSSSCT00000027320.2 | ATPase Na+/K+ transporting subunit alpha 1 | -0.812861098 | 0.036752 |
| ENSSSCT00000045379.1 | eukaryotic translation initiation factor 4 gamma 3 | -0.8085502 | 0.02519 |
| ENSSSCT00000066126.1 | scinderin | -0.798240311 | 0.012794 |
| ENSSSCT00000014835.3 | calmodulin regulated spectrin associated protein family member 3 | -0.797498866 | 0.040494 |
| ENSSSCT00000007115.3 | lamin A/C | -0.795646865 | 0.002161 |
| ENSSSCT00000002650.3 | Fos proto-oncogene, AP-1 transcription factor subunit | -0.793087668 | 0.025899 |
| ENSSSCT00000012072.3 | cubilin | -0.791554745 | 0.03909 |
| ENSSSCT00000017645.3 | indian hedgehog | -0.790302825 | 0.009266 |
| ENSSSCT00000014648.3 | adrenomedullin | -0.788426872 | 0.045029 |
| ENSSSCT00000008342.3 | BAI1 associated protein 2 like 1 | -0.786897194 | 0.003007 |
| ENSSSCT00000055405.1 | hydroxysteroid 17-beta dehydrogenase 2 | -0.785888049 | 0.029075 |
| ENSSSCT00000064614.1 | splA/ryanodine receptor domain and SOCS box containing 4 | -0.775149454 | 0.032982 |
| ENSSSCT00000001722.3 | TEA domain transcription factor 3 | -0.774718376 | 0.040532 |
| ENSSSCT00000060403.1 | replication initiator 1 | -0.770893988 | 0.016876 |
| ENSSSCT00000040794.1 | interleukin 22 receptor subunit alpha 1 | -0.770255763 | 0.027672 |
| ENSSSCT00000008434.3 | heat shock protein family B (small) member 1 | -0.76973349 | 0.009377 |
| ENSSSCT00000033295.2 | coiled-coil domain containing 120 | -0.768971566 | 0.009035 |
| ENSSSCT00000007472.3 | glutathione S-transferase mu 3 | -0.764183372 | 0.018802 |
| ENSSSCT00000061681.1 | argininosuccinate synthase 1 | -0.763139738 | 0.006556 |
| ENSSSCT00000019029.3 | Rap guanine nucleotide exchange factor like 1 | -0.762804588 | 0.034904 |
| ENSSSCT00000034483.2 | cytochrome P450 2J2 | -0.762346374 | 0.016705 |
| ENSSSCT00000047959.1 | BCAR1, Cas family scaffold protein | -0.755255534 | 0.035907 |
| ENSSSCT00000010052.3 | microsomal triglyceride transfer protein | -0.752575233 | 0.037371 |
| ENSSSCT00000022577.2 | zinc finger NFX1-type containing 1 | -0.752563481 | 0.013097 |
| ENSSSCT00000010966.3 | galactose-3-O-sulfotransferase 1 | -0.752004498 | 0.04897 |
| ENSSSCT00000063535.1 | citrate synthase | -0.748304501 | 0.007624 |
| ENSSSCT00000062592.1 | signal transducer and activator of transcription 5B | -0.738246517 | 0.048146 |
| ENSSSCT00000058648.1 | solute carrier family 6 member 8 | -0.736144982 | 0.023192 |
| ENSSSCT00000031995.2 | myosin heavy chain 14 | -0.7337543 | 0.016812 |
| ENSSSCT00000054120.1 | LLGL2, scribble cell polarity complex component | -0.726723039 | 0.004491 |
| ENSSSCT00000006270.3 | argininosuccinate synthase 1 | -0.722949961 | 0.02623 |
| ENSSSCT00000001357.3 | tripartite motif containing 31 | -0.722875293 | 0.022116 |
| ENSSSCT00000039357.1 |  | -0.721848158 | 0.000725 |
| ENSSSCT00000006210.3 | solute carrier family 27 member 4 | -0.721180678 | 0.011569 |
| ENSSSCT00000052253.1 | serine peptidase inhibitor, Kunitz type 1 | -0.720656849 | 0.007674 |
| ENSSSCT00000014048.3 | cadherin related family member 5 | -0.720446478 | 0.031827 |
| ENSSSCT00000008270.3 | GATA binding protein 5 | -0.713823202 | 0.005427 |
| ENSSSCT00000033745.2 | monoamine oxidase B | -0.711858912 | 0.041992 |
| ENSSSCT00000047581.1 | Fc fragment of IgG receptor and transporter | -0.710039523 | 0.039531 |
| ENSSSCT00000052143.1 | 5'-nucleotidase, cytosolic II | -0.708509183 | 0.049908 |
| ENSSSCT00000019007.3 | keratin 20 | -0.706942341 | 0.009842 |
| ENSSSCT00000003272.3 | signal induced proliferation associated 1 like 3 | -0.705868866 | 0.027852 |
| ENSSSCT00000012494.4 | cytokine inducible SH2 containing protein | -0.702211232 | 0.002367 |
| ENSSSCT00000007255.3 | cingulin | -0.701742319 | 0.008538 |
| ENSSSCT00000047176.1 | desmocollin 3 | -0.700524077 | 0.027793 |
| ENSSSCT00000035772.2 | interferon-induced transmembrane protein 1 | -0.700131904 | 0.000384 |
| ENSSSCT00000013009.3 | nuclear receptor subfamily 1 group I member 2 | -0.69977307 | 0.007504 |
| ENSSSCT00000009460.3 | cytidine/uridine monophosphate kinase 2 | -0.69784836 | 0.02987 |
| ENSSSCT00000018974.2 | junction plakoglobin | -0.697751689 | 0.002953 |
| ENSSSCT00000006698.3 | cadherin 17 | -0.695241704 | 0.009315 |
| ENSSSCT00000043113.1 | protein kinase C zeta | -0.695136244 | 0.04863 |
| ENSSSCT00000060599.1 | MAF bZIP transcription factor | -0.695046018 | 0.00792 |
| ENSSSCT00000026262.2 | DEAQ-box RNA dependent ATPase 1 | -0.695006217 | 0.017651 |
| ENSSSCT00000044396.1 | ladinin 1 | -0.694796907 | 0.021195 |
| ENSSSCT00000047703.1 | integrin linked kinase | -0.694534466 | 0.026118 |
| ENSSSCT00000027246.2 | Y-box binding protein 3 | -0.692371121 | 0.022657 |
| ENSSSCT00000005019.3 | SPG21, maspardin | -0.690476657 | 0.028576 |
| ENSSSCT00000000117.3 | BAI1 associated protein 2 like 2 | -0.688530965 | 0.005903 |
| ENSSSCT00000053227.1 | beta-1,3-galactosyltransferase 5 | -0.688160344 | 0.047291 |
| ENSSSCT00000017903.4 | amine oxidase, copper containing 1 | -0.68399093 | 0.004246 |
| ENSSSCT00000048990.1 | myosin ID | -0.683871983 | 0.01484 |
| ENSSSCT00000014043.3 | EPS8 like 2 | -0.683570384 | 0.010912 |
| ENSSSCT00000035232.2 | interferon regulatory factor 7 | -0.679819472 | 0.032831 |
| ENSSSCT00000053989.1 | espin | -0.67927925 | 0.042431 |
| ENSSSCT00000035657.2 | integrin subunit beta 4 | -0.677436388 | 0.030598 |
| ENSSSCT00000017236.3 | zinc finger protein 703 | -0.676849782 | 0.02701 |
| ENSSSCT00000028658.2 | FLYWCH family member 2 | -0.676451042 | 0.04744 |
| ENSSSCT00000053475.1 | potassium voltage-gated channel subfamily Q member 1 | -0.673772371 | 0.028003 |
| ENSSSCT00000035626.2 | alanyl aminopeptidase, membrane | -0.673243288 | 0.008733 |
| ENSSSCT00000054565.1 | family with sequence similarity 3 member B | -0.672962031 | 0.026437 |
| ENSSSCT00000007332.3 | PDZ domain containing 1 | -0.672014199 | 0.021344 |
| ENSSSCT00000033376.2 | dual specificity phosphatase 1 | -0.671340247 | 0.010664 |
| ENSSSCT00000027640.2 | claudin 3 | -0.67121406 | 0.010586 |
| ENSSSCT00000025725.2 | Rho guanine nucleotide exchange factor 5 | -0.671200244 | 0.049418 |
| ENSSSCT00000005707.3 | SH3 domain containing GRB2 like 2, endophilin A1 | -0.670544684 | 0.048892 |
| ENSSSCT00000015609.2 | sosondowah ankyrin repeat domain family member A | -0.66959317 | 0.027293 |
| ENSSSCT00000061821.1 | aldolase, fructose-bisphosphate B | -0.669526791 | 0.046797 |
| ENSSSCT00000043606.1 | RAB5B, member RAS oncogene family | -0.668827429 | 0.007008 |
| ENSSSCT00000008291.2 | MAF bZIP transcription factor K | -0.664858766 | 0.00798 |
| ENSSSCT00000044005.1 |  | -0.663139916 | 0.002195 |
| ENSSSCT00000030569.2 | Rho guanine nucleotide exchange factor 16 | -0.660400763 | 0.019916 |
| ENSSSCT00000055492.1 | filamin binding LIM protein 1 | -0.658606447 | 0.018767 |
| ENSSSCT00000001744.3 | cyclin dependent kinase inhibitor 1A | -0.658296222 | 0.005552 |
| ENSSSCT00000018638.3 | solute carrier family 6 member 19 | -0.657730955 | 0.037803 |
| ENSSSCT00000029188.2 | ubiquitin specific peptidase 16 | -0.654962527 | 0.03199 |
| ENSSSCT00000017637.3 | villin 1 | -0.654308491 | 0.025009 |
| ENSSSCT00000014247.3 | phospholipase C beta 3 | -0.653194747 | 0.009727 |
| ENSSSCT00000016228.3 | calpain 5 | -0.652812461 | 0.010665 |
| ENSSSCT00000054607.1 | mitotic spindle positioning | -0.650333446 | 0.00308 |
| ENSSSCT00000052139.1 | aldolase, fructose-bisphosphate B | -0.648825018 | 0.036348 |
| ENSSSCT00000038646.1 | keratin 8 | -0.646963545 | 0.00176 |
| ENSSSCT00000025711.2 | TNF superfamily member 10 | -0.646527583 | 0.042818 |
| ENSSSCT00000010207.3 | caudal type homeobox 2 | -0.64285543 | 0.005492 |
| ENSSSCT00000033364.2 | solute carrier family 44 member 4 | -0.637054201 | 0.007486 |
| ENSSSCT00000006567.2 | zinc fingers and homeoboxes 1 | -0.637043793 | 0.031789 |
| ENSSSCT00000036224.2 | crystallin beta-gamma domain containing 1 | -0.635257895 | 0.047214 |
| ENSSSCT00000065119.1 | serine protease 8 | -0.634454124 | 0.028594 |
| ENSSSCT00000054391.1 | C-terminal binding protein 2 | -0.633935325 | 0.042997 |
| ENSSSCT00000023162.2 | sushi domain containing 2 | -0.633138398 | 0.031009 |
| ENSSSCT00000027155.2 | G protein subunit alpha 11 | -0.632582749 | 0.041673 |
| ENSSSCT00000042569.1 | NBL1, DAN family BMP antagonist | -0.631011854 | 0.038578 |
| ENSSSCT00000015354.3 | cadherin related family member 2 | -0.630308366 | 0.031566 |
| ENSSSCT00000013962.3 | solute carrier family 6 member 8 | -0.629162086 | 0.006197 |
| ENSSSCT00000012349.3 | vasoactive intestinal peptide receptor 1 | -0.62869576 | 0.023712 |
| ENSSSCT00000007815.3 | mitochondrial antiviral signaling protein | -0.62637997 | 0.015455 |
| ENSSSCT00000004101.3 | GATA binding protein 6 | -0.625762569 | 0.030346 |
| ENSSSCT00000052265.1 | erythrocyte membrane protein band 4.1 like 3 | -0.620475935 | 0.013842 |
| ENSSSCT00000051045.1 | tripartite motif containing 40 | -0.618099062 | 0.045909 |
| ENSSSCT00000022558.2 | mal, T cell differentiation protein like | -0.618007928 | 0.025052 |
| ENSSSCT00000015600.4 | interferon regulatory factor 1 | -0.617698697 | 0.030561 |
| ENSSSCT00000003711.3 | dishevelled segment polarity protein 1 | -0.616578782 | 0.003819 |
| ENSSSCT00000027366.2 | protein tyrosine phosphatase, receptor type H | -0.615305982 | 0.02813 |
| ENSSSCT00000047765.1 | spermatogenesis associated serine rich 2 like | -0.614951571 | 0.035897 |
| ENSSSCT00000041822.1 | spectrin alpha, non-erythrocytic 1 | -0.614343583 | 0.025713 |
| ENSSSCT00000038816.1 | intestine specific homeobox | -0.613787628 | 0.045524 |
| ENSSSCT00000064586.1 | transmembrane channel like 5 | -0.613738761 | 0.045196 |
| ENSSSCT00000064052.1 | taperin | -0.609581253 | 0.027918 |
| ENSSSCT00000062201.1 | CD46 molecule, complement regulatory protein | -0.608590955 | 0.045045 |
| ENSSSCT00000033465.2 | syndecan 1 | -0.608389272 | 0.027361 |
| ENSSSCT00000017714.3 | integral membrane protein 2C | -0.606095905 | 0.01335 |
| ENSSSCT00000016232.3 | p21 (RAC1) activated kinase 1 | -0.605125732 | 0.011691 |
| ENSSSCT00000005915.3 | coronin 2A | -0.604806174 | 0.025556 |
| ENSSSCT00000006919.3 | glycoprotein A33 | -0.603360901 | 0.039977 |
| ENSSSCT00000007931.3 | nucleolar protein 4 like | -0.600786883 | 0.034019 |
| ENSSSCT00000017275.3 | myosin VIIB | -0.599238152 | 0.035193 |
| ENSSSCT00000041873.1 | zinc finger and BTB domain containing 7B | -0.598898198 | 0.015734 |
| ENSSSCT00000003627.3 | transmembrane channel like 4 | -0.596705502 | 0.020453 |
| ENSSSCT00000024524.2 | protein phosphatase 4 regulatory subunit 1 | -0.595407012 | 0.010039 |
| ENSSSCT00000007385.3 | ATPase Na+/K+ transporting subunit alpha 1 | -0.595135302 | 0.031798 |
| ENSSSCT00000018994.3 | keratin 14 | -0.593412008 | 0.000693 |
| ENSSSCT00000015025.3 | nucleus accumbens associated 1 | -0.592243561 | 0.049783 |
| ENSSSCT00000057986.1 | cadherin related family member 5 | -0.591193548 | 0.025112 |
| ENSSSCT00000037119.1 | solute carrier family 25 member 10 | -0.590728664 | 0.00896 |
| ENSSSCT00000005258.3 | serine peptidase inhibitor, Kunitz type 1 | -0.586995632 | 0.024155 |
| ENSSSCT00000060056.1 | GRAM domain containing 2B | -0.58686225 | 0.037627 |
| ENSSSCT00000057200.1 |  | -0.584939776 | 0.043352 |
| ENSSSCT00000022376.2 | ring finger protein 213 | -0.584485377 | 0.010513 |
| ENSSSCT00000039236.1 | claudin 2 | -0.578511259 | 0.048572 |
| ENSSSCT00000030079.2 | solute carrier family 6 member 20 | -0.574336191 | 0.049919 |
| ENSSSCT00000002982.3 | c-Maf inducing protein | -0.573862186 | 0.047907 |
| ENSSSCT00000025910.2 | solute carrier family 23 member 1 | -0.572121546 | 0.019234 |
| ENSSSCT00000038559.1 |  | -0.571515403 | 0.047992 |
| ENSSSCT00000064302.1 | hepatocyte nuclear factor 4 alpha | -0.571280676 | 0.018588 |
| ENSSSCT00000007427.3 | CTTNBP2 N-terminal like | -0.569711448 | 0.036788 |
| ENSSSCT00000006593.3 | exostosin glycosyltransferase 1 | -0.565945851 | 0.029028 |
| ENSSSCT00000041445.1 | leukocyte receptor cluster member 8 | -0.562170856 | 0.031646 |
| ENSSSCT00000026445.2 | proline rich 15 like | -0.552307117 | 0.035052 |
| ENSSSCT00000004328.3 | microtubule associated serine/threonine kinase 2 | -0.551594787 | 0.035999 |
| ENSSSCT00000054808.1 | N-myc downstream regulated 1 | -0.544883887 | 0.015414 |
| ENSSSCT00000003977.3 | sphingomyelin phosphodiesterase acid like 3B | -0.544282992 | 0.046983 |
| ENSSSCT00000042318.1 | RAB11 family interacting protein 1 | -0.543064204 | 0.03124 |
| ENSSSCT00000045040.1 | terminal nucleotidyltransferase 4B | -0.542739857 | 0.034635 |
| ENSSSCT00000026508.2 | carcinoembryonic antigen related cell adhesion molecule 1 | -0.542589948 | 0.030077 |
| ENSSSCT00000050794.1 | eukaryotic translation initiation factor 2 alpha kinase 2 | -0.542361351 | 0.03023 |
| ENSSSCT00000006498.2 | diacylglycerol O-acyltransferase 1 | -0.540683717 | 0.045852 |
| ENSSSCT00000036699.1 | inositol-tetrakisphosphate 1-kinase | -0.538145675 | 0.00792 |
| ENSSSCT00000019254.3 | dehydrogenase/reductase 11 | -0.535704086 | 0.039067 |
| ENSSSCT00000015156.3 | nuclear receptor subfamily 2 group F member 6 | -0.53452429 | 0.005255 |
| ENSSSCT00000026198.2 | RB1 inducible coiled-coil 1 | -0.53067503 | 0.03248 |
| ENSSSCT00000008831.2 | potassium channel tetramerization domain containing 5 | -0.530296045 | 0.027954 |
| ENSSSCT00000058172.1 | HERV-H LTR-associating 2 | -0.52969751 | 0.040793 |
| ENSSSCT00000034737.2 | interferon regulatory factor 6 | -0.529676198 | 0.046294 |
| ENSSSCT00000031059.2 | serine incorporator 2 | -0.529641982 | 0.03187 |
| ENSSSCT00000036524.2 | tripartite motif containing 26 | -0.527442545 | 0.010276 |
| ENSSSCT00000014016.4 | membrane palmitoylated protein 1 | -0.526879002 | 0.006027 |
| ENSSSCT00000053446.1 | mitogen-activated protein kinase 3 | -0.525879283 | 0.00123 |
| ENSSSCT00000003186.3 | lipolysis stimulated lipoprotein receptor | -0.524691101 | 0.01826 |
| ENSSSCT00000065002.1 | tryptophanyl-tRNA synthetase | -0.524377019 | 0.036308 |
| ENSSSCT00000043828.1 | zinc finger protein 358 | -0.523864985 | 0.049478 |
| ENSSSCT00000015792.3 | caudal type homeobox 1 | -0.519602658 | 0.041956 |
| ENSSSCT00000007975.3 | transient receptor potential cation channel subfamily C member 4 associated protein | -0.518383923 | 0.003478 |
| ENSSSCT00000000458.3 | myosin IA | -0.51661297 | 0.022674 |
| ENSSSCT00000016613.3 | amyloid beta precursor like protein 2 | -0.516307554 | 0.017072 |
| ENSSSCT00000027266.2 | galectin 4 | -0.514929953 | 0.026 |
| ENSSSCT00000027444.2 | major vault protein | -0.514176956 | 0.000397 |
| ENSSSCT00000015666.3 | catenin alpha 1 | -0.513739521 | 0.039859 |
| ENSSSCT00000055104.1 | pinin, desmosome associated protein | -0.512821733 | 0.031755 |
| ENSSSCT00000003289.3 | actinin alpha 4 | -0.509032083 | 0.013709 |
| ENSSSCT00000001103.3 | serpin family B member 1 | -0.508951221 | 0.012126 |
| ENSSSCT00000019051.3 | protein phosphatase 1 regulatory inhibitor subunit 1B | -0.50815978 | 0.025968 |
| ENSSSCT00000029636.2 | suppressor of cytokine signaling 1 | -0.505578276 | 0.048899 |
| ENSSSCT00000004219.3 | phosphoglucomutase 1 | -0.504385542 | 0.004587 |
| ENSSSCT00000042151.1 | cortactin | -0.50425985 | 0.031708 |
| ENSSSCT00000018774.3 | CDC42 effector protein 4 | -0.503850325 | 0.010628 |
| ENSSSCT00000016661.3 | cyclin dependent kinase 18 | -0.501229151 | 0.046198 |
| ENSSSCT00000005194.3 | creatine kinase, mitochondrial 1A | -0.500866173 | 0.013388 |
| ENSSSCT00000015619.3 | voltage dependent anion channel 1 | -0.497789702 | 0.010637 |
| ENSSSCT00000007471.3 | EPS8 like 3 | -0.494700705 | 0.033147 |
| ENSSSCT00000060796.1 | mitogen-activated protein kinase 3 | -0.494571469 | 0.03518 |
| ENSSSCT00000053199.1 | JunD proto-oncogene, AP-1 transcription factor subunit | -0.492890842 | 0.00021 |
| ENSSSCT00000001629.3 | transporter 2, ATP binding cassette subfamily B member | -0.491054398 | 0.039721 |
| ENSSSCT00000005901.2 | aldehyde dehydrogenase 1 family member B1 | -0.489756855 | 0.020494 |
| ENSSSCT00000012988.3 | poly(ADP-ribose) polymerase family member 14 | -0.48814864 | 0.017396 |
| ENSSSCT00000000402.3 | sulfite oxidase | -0.487640818 | 0.001344 |
| ENSSSCT00000016615.3 | suppression of tumorigenicity 14 | -0.481854139 | 0.017456 |
| ENSSSCT00000003428.4 | nectin cell adhesion molecule 2 | -0.480367959 | 0.038895 |
| ENSSSCT00000049917.1 | galectin 3 binding protein | -0.478417898 | 0.014028 |
| ENSSSCT00000030279.3 | CD82 molecule | -0.478156943 | 0.042661 |
| ENSSSCT00000018759.3 | SLC9A3 regulator 1 | -0.47168707 | 0.032281 |
| ENSSSCT00000052362.1 | NAD kinase | -0.469015605 | 0.009122 |
| ENSSSCT00000053600.1 | Rho GTPase activating protein 17 | -0.465184186 | 0.041568 |
| ENSSSCT00000003473.3 | KDEL endoplasmic reticulum protein retention receptor 1 | -0.463590382 | 0.031546 |
| ENSSSCT00000059164.1 | catenin delta 1 | -0.463376658 | 0.03847 |
| ENSSSCT00000045268.1 | ETS2 repressor factor | -0.462573822 | 0.043586 |
| ENSSSCT00000019491.3 | misshapen like kinase 1 | -0.462347112 | 0.044849 |
| ENSSSCT00000051241.1 | caspase 7 | -0.459949436 | 0.045651 |
| ENSSSCT00000040939.1 | oxoglutarate dehydrogenase | -0.456336938 | 0.034501 |
| ENSSSCT00000006674.3 | lysosomal protein transmembrane 4 beta | -0.454353076 | 0.046774 |
| ENSSSCT00000035483.2 | interferon regulatory factor 2 | -0.453433024 | 0.014704 |
| ENSSSCT00000015312.3 | alpha-1,3-mannosyl-glycoprotein 4-beta-N-acetylglucosaminyltransferase B | -0.452213418 | 0.033731 |
| ENSSSCT00000026750.2 | ZFP36 ring finger protein | -0.450421013 | 0.001482 |
| ENSSSCT00000012552.3 | abhydrolase domain containing 6 | -0.449088974 | 0.040111 |
| ENSSSCT00000002240.4 | interferon regulatory factor 9 | -0.447813673 | 0.002543 |
| ENSSSCT00000011309.3 | annexin A11 | -0.447380234 | 0.034305 |
| ENSSSCT00000003721.3 | cyclin-dependent kinase 11B | -0.445576728 | 0.025446 |
| ENSSSCT00000035478.2 | tripartite motif containing 25 | -0.445111822 | 0.046911 |
| ENSSSCT00000014123.3 | coronin 1B | -0.441036861 | 0.036195 |
| ENSSSCT00000058692.1 | cadherin 1 | -0.440344737 | 0.047936 |
| ENSSSCT00000011991.3 | golgi membrane protein 1 | -0.440293184 | 0.043922 |
| ENSSSCT00000005858.3 | glucosylceramidase beta 2 | -0.439638497 | 0.008466 |
| ENSSSCT00000043698.1 | catechol-O-methyltransferase | -0.438336236 | 0.048622 |
| ENSSSCT00000034734.2 | CD151 molecule (Raph blood group) | -0.427362466 | 0.040359 |
| ENSSSCT00000045280.1 | optineurin | -0.426736201 | 0.044594 |
| ENSSSCT00000024821.2 | caspase recruitment domain family member 6 | -0.418302372 | 0.029675 |
| ENSSSCT00000017822.3 | arginyl aminopeptidase like 1 | -0.417720596 | 0.017011 |
| ENSSSCT00000038507.1 | protein FAM127 | -0.416613825 | 0.006724 |
| ENSSSCT00000050866.1 | protein FAM127 | -0.416613825 | 0.006724 |
| ENSSSCT00000055164.1 | protein FAM127 | -0.416613825 | 0.006724 |
| ENSSSCT00000065043.1 | protein FAM127 | -0.416613825 | 0.006724 |
| ENSSSCT00000048326.1 | aldolase, fructose-bisphosphate A | -0.414260473 | 0.002424 |
| ENSSSCT00000053991.1 | RAB7A, member RAS oncogene family | -0.411494139 | 0.020778 |
| ENSSSCT00000052506.1 | PTTG1 interacting protein | -0.410987174 | 0.02377 |
| ENSSSCT00000024290.2 | sphingosine kinase 2 | -0.41064451 | 0.039214 |
| ENSSSCT00000005376.2 | carnosine dipeptidase 2 | -0.408183355 | 0.037832 |
| ENSSSCT00000047748.1 | ubiquilin 1 | -0.403959578 | 0.013434 |
| ENSSSCT00000009288.3 | galactose mutarotase | -0.401706867 | 0.027117 |
| ENSSSCT00000057853.1 | high mobility group 20B | -0.399148265 | 0.026205 |
| ENSSSCT00000053609.1 | nicotinate phosphoribosyltransferase | -0.39118245 | 0.038693 |
| ENSSSCT00000000393.3 | diacylglycerol kinase alpha | -0.389261591 | 0.033589 |
| ENSSSCT00000000070.3 | aconitase 2 | -0.385036871 | 0.015037 |
| ENSSSCT00000045529.1 | epsin 1 | -0.384699733 | 0.022193 |
| ENSSSCT00000014210.3 | calpain 1 | -0.383613493 | 0.034234 |
| ENSSSCT00000017250.3 | protein phosphatase 2 catalytic subunit beta | -0.382141435 | 0.01157 |
| ENSSSCT00000044391.1 | ferritin heavy chain 1 | -0.381745842 | 0.02604 |
| ENSSSCT00000036014.2 | B cell CLL/lymphoma 3 | -0.381523765 | 0.029941 |
| ENSSSCT00000039383.1 | LIM and SH3 protein 1 | -0.380693745 | 0.020894 |
| ENSSSCT00000048970.1 | glucose-6-phosphate dehydrogenase | -0.380411269 | 0.045392 |
| ENSSSCT00000030348.2 | WW domain binding protein 2 | -0.379599975 | 0.016096 |
| ENSSSCT00000042486.1 | ZFP36 ring finger protein like 2 | -0.379194388 | 0.031464 |
| ENSSSCT00000027356.2 | leucine aminopeptidase 3 | -0.375485611 | 0.026612 |
| ENSSSCT00000000771.3 | TNF receptor superfamily member 1A | -0.373865349 | 0.049106 |
| ENSSSCT00000052410.1 | ETS proto-oncogene 2, transcription factor | -0.371607181 | 0.046343 |
| ENSSSCT00000011617.3 | adducin 3 | -0.368992286 | 0.0412 |
| ENSSSCT00000001752.3 | Pim-1 proto-oncogene, serine/threonine kinase | -0.368650792 | 0.03851 |
| ENSSSCT00000059458.1 | eukaryotic translation initiation factor 4E binding protein 2 | -0.363168385 | 0.048932 |
| ENSSSCT00000005250.3 | calcineurin like EF-hand protein 1 | -0.360951554 | 0.012187 |
| ENSSSCT00000064289.1 | plasmalemma vesicle associated protein | -0.360784615 | 0.020741 |
| ENSSSCT00000044582.1 | LDL receptor related protein 10 | -0.358116658 | 0.015784 |
| ENSSSCT00000046595.1 | KH and NYN domain containing | -0.357980748 | 0.047455 |
| ENSSSCT00000032084.2 | calmodulin 3 | -0.356696993 | 0.030004 |
| ENSSSCT00000056298.1 | basic helix-loop-helix family member e40 | -0.355201965 | 0.045565 |
| ENSSSCT00000003841.3 | EF-hand domain family member D2 | -0.351429966 | 0.00954 |
| ENSSSCT00000010750.2 | ras homolog family member F, filopodia associated | -0.347752872 | 0.028006 |
| ENSSSCT00000049038.1 | chromosome 6 open reading frame 106 | -0.33850721 | 0.011025 |
| ENSSSCT00000010474.3 | lysosomal associated membrane protein 1 | -0.325757187 | 0.031075 |
| ENSSSCT00000057478.1 | signal transducer and activator of transcription 3 | -0.319921299 | 0.013099 |
| ENSSSCT00000061753.1 | X-prolyl aminopeptidase 1 | -0.316887648 | 0.045313 |
| ENSSSCT00000031590.2 | calpain small subunit 1 | -0.307123885 | 0.004117 |
| ENSSSCT00000055030.1 | G protein subunit beta 1 | -0.306924151 | 0.032796 |
| ENSSSCT00000024166.2 | farnesyl-diphosphate farnesyltransferase 1 | -0.30185388 | 0.026743 |
| ENSSSCT00000003144.3 | lon peptidase 2, peroxisomal | -0.297723601 | 0.024523 |
| ENSSSCT00000027876.2 | dynein light chain LC8-type 2 | -0.294471561 | 0.032887 |
| ENSSSCT00000017482.3 | signal transducer and activator of transcription 1 | -0.291844378 | 0.016575 |
| ENSSSCT00000012434.3 | shisa family member 5 | -0.287418887 | 0.009007 |
| ENSSSCT00000010428.3 | serine/threonine kinase 24 | -0.283783724 | 0.035402 |
| ENSSSCT00000043045.1 | yippee like 3 | -0.280875198 | 0.03005 |
| ENSSSCT00000022886.2 | phosphatidylinositol transfer protein alpha | -0.274779357 | 0.049248 |
| ENSSSCT00000053102.1 | TAP binding protein | -0.239702025 | 0.01809 |
| ENSSSCT00000007085.3 | heparin binding growth factor | -0.235112921 | 0.046459 |
| ENSSSCT00000039540.1 | Rac family small GTPase 1 | -0.21993066 | 0.029369 |
| ENSSSCT00000033211.2 | cell division cycle 42 | 0.166304601 | 0.006792 |
| ENSSSCT00000015371.3 | basic transcription factor 3 | 0.167546649 | 0.027927 |
| ENSSSCT00000008489.3 | coiled-coil-helix-coiled-coil-helix domain containing 2 | 0.189460866 | 0.013513 |
| ENSSSCT00000046697.1 | eukaryotic translation initiation factor 3 subunit H | 0.191197967 | 0.03215 |
| ENSSSCT00000043535.1 | splicing factor 3b subunit 5 | 0.197246081 | 0.049198 |
| ENSSSCT00000006622.3 | eukaryotic translation initiation factor 3 subunit E | 0.197902235 | 0.04416 |
| ENSSSCT00000004960.3 | eukaryotic translation elongation factor 1 alpha 1 | 0.199690398 | 0.02828 |
| ENSSSCT00000028257.2 | selenoprotein F | 0.21564025 | 0.044421 |
| ENSSSCT00000005522.2 | ribosomal protein L36a like | 0.216476683 | 0.049373 |
| ENSSSCT00000004049.3 | NADH:ubiquinone oxidoreductase subunit S5 | 0.216929304 | 0.007614 |
| ENSSSCT00000065836.1 | proteasome subunit alpha 4 | 0.223919876 | 0.038353 |
| ENSSSCT00000035556.2 | ribosomal protein L39 | 0.229188188 | 0.043566 |
| ENSSSCT00000002630.3 | NPC intracellular cholesterol transporter 2 | 0.230293096 | 0.038322 |
| ENSSSCT00000052670.1 | ABRA C-terminal like | 0.232996645 | 0.033949 |
| ENSSSCT00000063468.1 | LSM4 homolog, U6 small nuclear RNA and mRNA degradation associated | 0.233357366 | 0.045766 |
| ENSSSCT00000009320.3 | dpy-30, histone methyltransferase complex regulatory subunit | 0.234455348 | 0.028077 |
| ENSSSCT00000018499.3 | ribosomal protein L26 like 1 | 0.237226582 | 0.045329 |
| ENSSSCT00000018047.3 | ATPase H+ transporting V1 subunit F | 0.23814487 | 0.028266 |
| ENSSSCT00000051843.1 | ribosomal protein L23 | 0.240441452 | 0.047713 |
| ENSSSCT00000058189.1 | proteasome subunit beta 1 | 0.242303189 | 0.04043 |
| ENSSSCT00000012129.3 | COMM domain containing 3 | 0.243006981 | 0.034393 |
| ENSSSCT00000001170.3 | DEK proto-oncogene | 0.247354765 | 0.02895 |
| ENSSSCT00000014995.3 | WD repeat domain 83 opposite strand | 0.248842904 | 0.030607 |
| ENSSSCT00000018768.3 | ribosomal protein L38 | 0.249008259 | 0.036028 |
| ENSSSCT00000003161.3 | programmed cell death 5 | 0.250375779 | 0.039198 |
| ENSSSCT00000014628.3 | proteasome subunit alpha 1 | 0.251812596 | 0.024069 |
| ENSSSCT00000040918.1 | ribosomal protein L5 | 0.254059828 | 0.025099 |
| ENSSSCT00000026504.2 | serine/threonine kinase receptor associated protein | 0.257152905 | 0.014127 |
| ENSSSCT00000011019.3 | small nuclear ribonucleoprotein D3 polypeptide | 0.260817973 | 0.015024 |
| ENSSSCT00000054290.1 | synaptophysin like 1 | 0.261502475 | 0.018371 |
| ENSSSCT00000053253.1 | neural precursor cell expressed, developmentally down-regulated 8 | 0.261889117 | 0.047767 |
| ENSSSCT00000017625.3 | actin related protein 2/3 complex subunit 2 | 0.263342671 | 0.014502 |
| ENSSSCT00000012527.2 | signal peptidase complex subunit 1 | 0.264928162 | 0.020958 |
| ENSSSCT00000001810.3 | prickle planar cell polarity protein 4 | 0.270191419 | 0.041405 |
| ENSSSCT00000010191.3 | ribosomal protein L21 | 0.270409423 | 0.041509 |
| ENSSSCT00000012045.3 | NADH:ubiquinone oxidoreductase subunit B6 | 0.27463943 | 0.037643 |
| ENSSSCT00000011706.3 | NSE4 homolog A, SMC5-SMC6 complex component | 0.276101775 | 0.047573 |
| ENSSSCT00000010797.3 | phosphatidylethanolamine binding protein 1 | 0.27651817 | 0.013922 |
| ENSSSCT00000008725.3 | small nuclear ribonucleoprotein U11/U12 subunit 25 | 0.276686641 | 0.041315 |
| ENSSSCT00000006146.3 | proteasome subunit beta 7 | 0.276745987 | 0.02271 |
| ENSSSCT00000018737.3 | small ubiquitin-like modifier 2 | 0.277130487 | 0.021295 |
| ENSSSCT00000009878.3 | ribosomal protein S3A | 0.277709175 | 0.040772 |
| ENSSSCT00000052913.1 | male-enhanced antigen 1 | 0.277962228 | 0.023532 |
| ENSSSCT00000008352.3 | BUD31 homolog | 0.278848565 | 0.038512 |
| ENSSSCT00000063010.1 | ribosomal protein L3 | 0.281464903 | 0.023978 |
| ENSSSCT00000055147.1 | chloride intracellular channel 1 | 0.283273571 | 0.002476 |
| ENSSSCT00000023057.2 | small nuclear ribonucleoprotein polypeptide E | 0.283973731 | 0.023438 |
| ENSSSCT00000016207.3 | ribosomal protein S3 | 0.285275369 | 0.044034 |
| ENSSSCT00000022688.2 | COP9 signalosome subunit 9 | 0.285386363 | 0.012398 |
| ENSSSCT00000016235.3 | chloride nucleotide-sensitive channel 1A | 0.286222822 | 0.037945 |
| ENSSSCT00000023500.2 | proteasome subunit beta 1 | 0.286591335 | 0.010013 |
| ENSSSCT00000004346.3 | ribosomal protein S8 | 0.287152219 | 0.030131 |
| ENSSSCT00000003287.3 | eukaryotic translation initiation factor 3 subunit K | 0.287398762 | 0.045434 |
| ENSSSCT00000033835.2 | solute carrier family 44 member 1 | 0.288114322 | 0.043788 |
| ENSSSCT00000019062.3 | ribosomal protein L19 | 0.289221416 | 0.044672 |
| ENSSSCT00000035199.2 | VHL binding protein 1 | 0.290053769 | 0.031201 |
| ENSSSCT00000010647.3 | histone PARylation factor 1 | 0.291275151 | 0.034496 |
| ENSSSCT00000029514.2 | superoxide dismutase 1 | 0.291363416 | 0.033655 |
| ENSSSCT00000043325.1 | WT1 associated protein | 0.291794442 | 0.044086 |
| ENSSSCT00000027450.2 | RPL17-C18orf32 readthrough | 0.293898771 | 0.034625 |
| ENSSSCT00000049730.1 | ribosomal protein L22 | 0.294451351 | 0.038158 |
| ENSSSCT00000038492.1 | RPL17-C18orf32 readthrough | 0.296152461 | 0.031766 |
| ENSSSCT00000017130.3 | nucleolar protein interacting with the FHA domain of MKI67 | 0.296946562 | 0.009023 |
| ENSSSCT00000011543.3 | biogenesis of lysosomal organelles complex 1 subunit 2 | 0.298992045 | 0.029525 |
| ENSSSCT00000012340.3 | ribosomal protein L14 | 0.299091684 | 0.017611 |
| ENSSSCT00000019150.3 | NME/NM23 nucleoside diphosphate kinase 2 | 0.29999537 | 0.021876 |
| ENSSSCT00000000170.3 | PWP1 homolog, endonuclein | 0.30005227 | 0.031323 |
| ENSSSCT00000041552.1 | RB binding protein 4, chromatin remodeling factor | 0.300839776 | 0.044789 |
| ENSSSCT00000010015.3 | ribosomal protein L34 | 0.303210744 | 0.038032 |
| ENSSSCT00000001743.3 | serine and arginine rich splicing factor 3 | 0.303242388 | 0.0408 |
| ENSSSCT00000047389.1 | mediator of cell motility 1 | 0.303269161 | 0.034754 |
| ENSSSCT00000018928.3 | cytochrome c oxidase assembly factor 3 | 0.304493183 | 0.00989 |
| ENSSSCT00000000992.3 |  | 0.305451876 | 0.003797 |
| ENSSSCT00000008429.3 | RNA polymerase II subunit J | 0.305598321 | 0.035762 |
| ENSSSCT00000041673.1 | mitochondrial import inner membrane translocase subunit Tim23 | 0.305710719 | 0.037909 |
| ENSSSCT00000065973.1 | RNA guanine-7 methyltransferase activating subunit | 0.306523433 | 0.02052 |
| ENSSSCT00000012969.3 | ribosomal protein L35a | 0.30835837 | 0.046496 |
| ENSSSCT00000045760.1 | ribosomal protein S27a | 0.308882348 | 0.01357 |
| ENSSSCT00000044715.1 | glutaredoxin 3 | 0.309076775 | 0.019705 |
| ENSSSCT00000010014.3 | oligosaccharyltransferase complex non-catalytic subunit | 0.30907809 | 0.040959 |
| ENSSSCT00000011737.3 | BUB3, mitotic checkpoint protein | 0.313840734 | 0.031397 |
| ENSSSCT00000000103.3 | translocase of outer mitochondrial membrane 22 | 0.31384836 | 0.011095 |
| ENSSSCT00000056310.1 | ribosomal protein S6 | 0.313851293 | 0.015319 |
| ENSSSCT00000017585.3 | ribulose-5-phosphate-3-epimerase | 0.316605545 | 0.04062 |
| ENSSSCT00000001849.3 | mitochondrial ribosomal protein L2 | 0.319765417 | 0.046297 |
| ENSSSCT00000059081.1 | mitochondrial ribosomal protein L9 | 0.32032332 | 0.014278 |
| ENSSSCT00000051893.1 | glutathione peroxidase 4 | 0.321186882 | 0.023261 |
| ENSSSCT00000063313.1 | chromosome 11 open reading frame 58 | 0.321783338 | 0.033784 |
| ENSSSCT00000014280.3 | eukaryotic translation elongation factor 1 gamma | 0.323015392 | 0.023289 |
| ENSSSCT00000061185.1 | APAF1 interacting protein | 0.32428293 | 0.005615 |
| ENSSSCT00000014242.3 | tRNA methyltransferase subunit 11-2 | 0.325573036 | 0.03007 |
| ENSSSCT00000046956.1 | peptidylprolyl isomerase B | 0.326998611 | 0.0074 |
| ENSSSCT00000032480.2 | NOP16 nucleolar protein | 0.327677658 | 0.02172 |
| ENSSSCT00000004629.3 | syntaxin 7 | 0.329709801 | 0.023841 |
| ENSSSCT00000015437.3 | ribosomal protein S23 | 0.331351634 | 0.029692 |
| ENSSSCT00000045754.1 | ribosomal protein L38 | 0.331674619 | 0.024434 |
| ENSSSCT00000002569.3 | ERH, mRNA splicing and mitosis factor | 0.332829314 | 0.002992 |
| ENSSSCT00000019495.3 | proteasome subunit beta 6 | 0.332951444 | 0.021332 |
| ENSSSCT00000049763.1 | ribosomal protein lateral stalk subunit P0 | 0.333572908 | 0.014575 |
| ENSSSCT00000057837.1 | DNA polymerase epsilon 4, accessory subunit | 0.335723373 | 0.023623 |
| ENSSSCT00000016800.3 | peptidase, mitochondrial processing beta subunit | 0.336157929 | 0.008018 |
| ENSSSCT00000014167.3 | barrier to autointegration factor 1 | 0.338745092 | 0.047418 |
| ENSSSCT00000017506.3 | heat shock protein family E (Hsp10) member 1 | 0.339807891 | 0.022722 |
| ENSSSCT00000012095.3 | heat shock protein family A (Hsp70) member 14 | 0.340060779 | 0.04862 |
| ENSSSCT00000047169.1 |  | 0.340367263 | 0.031227 |
| ENSSSCT00000013152.2 | mitochondrial ribosomal protein L39 | 0.341362776 | 0.010681 |
| ENSSSCT00000024679.2 | transmembrane protein 70 | 0.341722113 | 0.035221 |
| ENSSSCT00000008962.3 | mitochondrial ribosomal protein L30 | 0.342998863 | 0.018682 |
| ENSSSCT00000064349.1 | RNA polymerase II subunit D | 0.34530543 | 0.035906 |
| ENSSSCT00000032308.2 | general transcription factor IIH subunit 5 | 0.346179897 | 0.012989 |
| ENSSSCT00000012875.3 | NADH:ubiquinone oxidoreductase subunit B5 | 0.346683627 | 0.030347 |
| ENSSSCT00000004261.3 | mago homolog, exon junction complex subunit | 0.348266826 | 0.002341 |
| ENSSSCT00000046282.1 | mitochondrial ribosomal protein L36 | 0.348610955 | 0.031596 |
| ENSSSCT00000016280.3 | cathepsin C | 0.349048071 | 0.005396 |
| ENSSSCT00000064812.1 | actin related protein 2/3 complex subunit 4 | 0.349760087 | 0.013321 |
| ENSSSCT00000055818.1 | heterogeneous nuclear ribonucleoprotein C (C1/C2) | 0.350295587 | 0.022922 |
| ENSSSCT00000010085.3 | PIGY upstream reading frame | 0.350466225 | 0.035271 |
| ENSSSCT00000028450.2 | small integral membrane protein 26 | 0.353849747 | 0.020513 |
| ENSSSCT00000003224.3 | TYRO protein tyrosine kinase binding protein | 0.35396457 | 0.028365 |
| ENSSSCT00000061488.1 | ribosomal protein L22 like 1 | 0.355625028 | 0.003498 |
| ENSSSCT00000047582.1 | translocase of outer mitochondrial membrane 5 | 0.355649731 | 0.021111 |
| ENSSSCT00000063697.1 | ATP synthase peripheral stalk-membrane subunit b | 0.35597208 | 0.002767 |
| ENSSSCT00000044750.1 | fatty acid binding protein 3 | 0.356312037 | 0.027557 |
| ENSSSCT00000004100.3 | small nuclear ribonucleoprotein D1 polypeptide | 0.35658595 | 0.022069 |
| ENSSSCT00000001616.3 | MHC class II histocompatibility antigen SLA-DQA | 0.357104863 | 0.024938 |
| ENSSSCT00000040250.1 | nucleolar and spindle associated protein 1 | 0.357386009 | 0.032108 |
| ENSSSCT00000000089.3 | ribosomal protein S19 binding protein 1 | 0.357544446 | 0.016966 |
| ENSSSCT00000057207.1 | eukaryotic translation initiation factor 1A X-linked | 0.358129792 | 0.031732 |
| ENSSSCT00000044369.1 | ribosomal protein L35a | 0.358960446 | 0.028015 |
| ENSSSCT00000028348.2 | cyclin dependent kinase 4 | 0.359299981 | 0.046688 |
| ENSSSCT00000031620.2 | SRA stem-loop interacting RNA binding protein | 0.361581527 | 0.008973 |
| ENSSSCT00000038200.1 |  | 0.362095416 | 0.012033 |
| ENSSSCT00000036496.2 | signal sequence receptor subunit 4 | 0.363047342 | 0.026161 |
| ENSSSCT00000028286.2 | small integral membrane protein 14 | 0.363124592 | 0.008413 |
| ENSSSCT00000058122.1 | complement C1s | 0.368107632 | 0.006205 |
| ENSSSCT00000043358.1 | ribosomal protein L10 | 0.368816956 | 0.011786 |
| ENSSSCT00000010685.3 | RAN, member RAS oncogene family | 0.369591102 | 0.015385 |
| ENSSSCT00000008539.3 | CD2 cytoplasmic tail binding protein 2 | 0.369750773 | 0.043832 |
| ENSSSCT00000001706.3 | small nuclear ribonucleoprotein polypeptide C | 0.370152075 | 0.016792 |
| ENSSSCT00000065326.1 | sorting nexin 5 | 0.370177385 | 0.034459 |
| ENSSSCT00000017560.3 | eukaryotic translation elongation factor 1 beta 2 | 0.372111207 | 0.007246 |
| ENSSSCT00000017279.3 | ADP ribosylation factor like GTPase 6 interacting protein 6 | 0.372717528 | 0.033524 |
| ENSSSCT00000059836.1 | small nuclear ribonucleoprotein D2 polypeptide | 0.375343229 | 0.036206 |
| ENSSSCT00000063143.1 | annexin A5 | 0.375916113 | 0.021574 |
| ENSSSCT00000036365.2 | ubiquitously expressed prefoldin like chaperone | 0.377750738 | 0.043034 |
| ENSSSCT00000022560.2 | RNA polymerase II subunit G | 0.37825085 | 0.016023 |
| ENSSSCT00000000979.3 | small nuclear ribonucleoprotein polypeptide F | 0.378336869 | 0.019453 |
| ENSSSCT00000032340.2 | transmembrane protein 258 | 0.380851311 | 0.002091 |
| ENSSSCT00000005100.2 | myosin VA | 0.381301056 | 0.031741 |
| ENSSSCT00000032799.2 | hydroxysteroid 17-beta dehydrogenase 10 | 0.383261197 | 0.005485 |
| ENSSSCT00000050385.1 | small nuclear ribonucleoprotein polypeptide B2 | 0.38435151 | 0.000433 |
| ENSSSCT00000012642.3 | actin related protein 2/3 complex subunit 4 | 0.384468121 | 0.015429 |
| ENSSSCT00000045113.1 | selenoprotein W | 0.38463521 | 0.020298 |
| ENSSSCT00000054246.1 |  | 0.390199959 | 0.016286 |
| ENSSSCT00000023105.2 | flavin adenine dinucleotide synthetase 1 | 0.391970345 | 0.016966 |
| ENSSSCT00000046306.1 | glutathione peroxidase 1 | 0.394671224 | 0.028264 |
| ENSSSCT00000045474.1 | ribosomal protein L10a | 0.395569612 | 0.002019 |
| ENSSSCT00000052062.1 | inosine triphosphatase | 0.396708243 | 0.007795 |
| ENSSSCT00000049267.1 | eukaryotic translation initiation factor 3 subunit K | 0.39941477 | 0.019275 |
| ENSSSCT00000045381.1 | lamin B receptor | 0.40114155 | 0.028036 |
| ENSSSCT00000052543.1 | ribosomal protein S7 | 0.402160003 | 0.009961 |
| ENSSSCT00000004747.3 | sorting nexin 14 | 0.402912746 | 0.002375 |
| ENSSSCT00000024602.2 | ribosomal protein S16 | 0.403047291 | 0.01513 |
| ENSSSCT00000019290.3 | chemokine (C-C motif) ligand 2 | 0.404724862 | 0.039797 |
| ENSSSCT00000026628.2 | FK506 binding protein 3 | 0.404974142 | 0.022554 |
| ENSSSCT00000016695.4 | tissue factor pathway inhibitor 2 | 0.405005292 | 0.035964 |
| ENSSSCT00000013661.4 | translocase of inner mitochondrial membrane 8A | 0.406027334 | 0.013433 |
| ENSSSCT00000010941.3 | zinc finger matrin-type 5 | 0.408502035 | 0.026107 |
| ENSSSCT00000013313.4 | peroxiredoxin 4 | 0.412871769 | 0.003618 |
| ENSSSCT00000011320.3 | peroxiredoxin like 2A | 0.412891787 | 0.016601 |
| ENSSSCT00000022802.2 | ribosomal protein L11 | 0.413596521 | 0.008467 |
| ENSSSCT00000054538.1 | ribosomal protein S9 | 0.41482126 | 0.017835 |
| ENSSSCT00000026012.2 | ubiquitin conjugating enzyme E2 E1 | 0.418632325 | 0.006332 |
| ENSSSCT00000039191.1 | high mobility group nucleosome binding domain 1 | 0.419694404 | 0.038504 |
| ENSSSCT00000001633.4 | MHC class II, DM beta | 0.423750794 | 0.007259 |
| ENSSSCT00000024316.2 | mitochondrial ribosomal protein L27 | 0.423898305 | 0.002538 |
| ENSSSCT00000061082.1 | peptidylprolyl isomerase like 1 | 0.424344424 | 0.023398 |
| ENSSSCT00000001963.3 | WD repeat domain 61 | 0.424777495 | 0.010316 |
| ENSSSCT00000055711.1 | deoxyribonuclease 1 like 3 | 0.425066091 | 0.045201 |
| ENSSSCT00000044885.1 | ribosomal protein L7a | 0.425454627 | 0.003845 |
| ENSSSCT00000000286.3 | chromosome 12 open reading frame 10 | 0.425854218 | 0.01546 |
| ENSSSCT00000001191.3 | geminin, DNA replication inhibitor | 0.426480996 | 0.040162 |
| ENSSSCT00000017369.3 | histone acetyltransferase 1 | 0.430713834 | 0.02666 |
| ENSSSCT00000001720.3 | ribosomal protein L10a | 0.432713908 | 0.003648 |
| ENSSSCT00000013257.4 | thymosin beta 4 X-linked | 0.433195625 | 0.000338 |
| ENSSSCT00000043395.1 | ARP3 actin related protein 3 homolog | 0.436241471 | 0.017138 |
| ENSSSCT00000051741.1 | CLN5, intracellular trafficking protein | 0.437127989 | 0.033975 |
| ENSSSCT00000053258.1 | thioredoxin related transmembrane protein 1 | 0.44025593 | 0.005663 |
| ENSSSCT00000044779.1 | cyclin dependent kinase inhibitor 2D | 0.442710047 | 0.036969 |
| ENSSSCT00000060340.1 | diazepam binding inhibitor, acyl-CoA binding protein | 0.446790338 | 0.001739 |
| ENSSSCT00000000203.2 | tubulin alpha 1a | 0.450135255 | 0.019508 |
| ENSSSCT00000040958.1 | thymopoietin | 0.451379559 | 0.028928 |
| ENSSSCT00000066241.1 | DnaJ heat shock protein family (Hsp40) member C10 | 0.45239141 | 0.00519 |
| ENSSSCT00000050367.1 | eukaryotic translation initiation factor 4A1 | 0.457440828 | 0.037324 |
| ENSSSCT00000011132.3 | nucleoside-triphosphatase, cancer-related | 0.45942014 | 0.049732 |
| ENSSSCT00000000963.3 | ARP6 actin related protein 6 homolog | 0.459500592 | 0.015363 |
| ENSSSCT00000042922.1 | transcription elongation factor A1 | 0.461212052 | 0.043352 |
| ENSSSCT00000012334.3 | ribosomal protein SA | 0.462213589 | 0.046739 |
| ENSSSCT00000034598.2 | RNA binding motif protein X-linked | 0.462923211 | 0.041606 |
| ENSSSCT00000018442.3 | ERCC excision repair 8, CSA ubiquitin ligase complex subunit | 0.463137833 | 0.031701 |
| ENSSSCT00000010604.3 | PDZ binding kinase | 0.463322458 | 0.04862 |
| ENSSSCT00000031595.2 | small nuclear ribonucleoprotein polypeptide G | 0.463563293 | 0.0017 |
| ENSSSCT00000010341.3 | regulator of cell cycle | 0.466199885 | 0.035518 |
| ENSSSCT00000040747.1 | G protein subunit gamma 2 | 0.468419919 | 0.038205 |
| ENSSSCT00000035781.2 | thymosin beta 4 X-linked | 0.47302364 | 0.00343 |
| ENSSSCT00000010764.3 | actin related protein 2/3 complex subunit 3 | 0.473190994 | 0.000883 |
| ENSSSCT00000018160.3 | gamma-glutamylcyclotransferase | 0.474019492 | 0.016287 |
| ENSSSCT00000052535.1 |  | 0.47543236 | 0.03524 |
| ENSSSCT00000030366.2 | origin recognition complex subunit 6 | 0.483475757 | 0.048479 |
| ENSSSCT00000006513.3 | chromatin accessibility complex subunit 1 | 0.489230449 | 0.013265 |
| ENSSSCT00000057636.1 | heat shock protein family E (Hsp10) member 1 | 0.491357431 | 0.036949 |
| ENSSSCT00000010051.3 | H2A histone family, member Z | 0.4946488 | 0.038315 |
| ENSSSCT00000056609.1 | acidic nuclear phosphoprotein 32 family member E | 0.499974782 | 0.033853 |
| ENSSSCT00000011398.4 | C-X-C motif chemokine ligand 12 | 0.500563613 | 0.009789 |
| ENSSSCT00000039797.1 | mitochondrial amidoxime reducing component 2 | 0.502726042 | 0.040956 |
| ENSSSCT00000034332.2 | mitochondrial ribosomal protein L13 | 0.506197202 | 0.006598 |
| ENSSSCT00000009286.3 | serine and arginine rich splicing factor 7 | 0.507014808 | 0.035016 |
| ENSSSCT00000006769.3 | lymphocyte antigen 96 | 0.507388669 | 0.019826 |
| ENSSSCT00000031675.2 | SCAN domain-containing 1-like | 0.509005444 | 0.039203 |
| ENSSSCT00000037000.1 | chromosome 12 open reading frame 45 | 0.513833907 | 0.049333 |
| ENSSSCT00000007515.4 | vascular cell adhesion molecule 1 | 0.51510985 | 0.035424 |
| ENSSSCT00000011474.3 | helicase, lymphoid specific | 0.515560647 | 0.041495 |
| ENSSSCT00000027484.2 | anaphase promoting complex subunit 4 | 0.521793213 | 0.038604 |
| ENSSSCT00000028667.2 | coenzyme Q3, methyltransferase | 0.524073905 | 0.049171 |
| ENSSSCT00000052270.1 | mitotic arrest deficient 2 like 1 | 0.524697416 | 0.033095 |
| ENSSSCT00000004969.3 | HAUS augmin like complex subunit 1 | 0.525157644 | 0.018275 |
| ENSSSCT00000030730.2 | acylphosphatase 2 | 0.527585719 | 0.021167 |
| ENSSSCT00000003053.3 | cytochrome b5 type B (outer mitochondrial membrane) | 0.527682569 | 0.007696 |
| ENSSSCT00000006211.3 | ubiquitin related modifier 1 | 0.534134453 | 0.017143 |
| ENSSSCT00000005573.3 | DLG associated protein 5 | 0.53620383 | 0.031985 |
| ENSSSCT00000042482.1 | transient receptor potential cation channel subfamily C member 4 associated protein | 0.536692825 | 0.02034 |
| ENSSSCT00000013703.2 | mortality factor 4 like 2 | 0.540842097 | 0.013435 |
| ENSSSCT00000027813.2 | replication protein A3 | 0.54701787 | 0.001098 |
| ENSSSCT00000060066.1 | proliferating cell nuclear antigen | 0.557635721 | 0.02555 |
| ENSSSCT00000047000.1 | ATP synthase membrane subunit c locus 3 | 0.558239823 | 0.045213 |
| ENSSSCT00000060390.1 | translocase of inner mitochondrial membrane 10B | 0.558772948 | 0.017217 |
| ENSSSCT00000001988.3 | transmembrane p24 trafficking protein 3 | 0.565919773 | 0.007191 |
| ENSSSCT00000003776.3 | apoptosis-inducing, TAF9-like domain 1 | 0.571847639 | 0.024781 |
| ENSSSCT00000047604.1 | protection of telomeres 1 | 0.572333655 | 0.043651 |
| ENSSSCT00000010246.3 | replication factor C subunit 3 | 0.572369906 | 0.046692 |
| ENSSSCT00000059621.1 | apurinic/apyrimidinic endodeoxyribonuclease 1 | 0.573937253 | 0.009221 |
| ENSSSCT00000026025.3 | CD59 molecule (CD59 blood group) | 0.57773033 | 0.0148 |
| ENSSSCT00000042794.1 | matrix Gla protein | 0.582405357 | 0.03019 |
| ENSSSCT00000050685.1 | ubiquitin like modifier activating enzyme 2 | 0.58361321 | 0.006442 |
| ENSSSCT00000050632.1 | cyclin dependent kinase 1 | 0.586916603 | 0.042769 |
| ENSSSCT00000009866.3 | meiotic nuclear divisions 1 | 0.587023261 | 0.024206 |
| ENSSSCT00000005031.3 | PCNA clamp associated factor | 0.59195851 | 0.021588 |
| ENSSSCT00000037544.1 | high mobility group nucleosomal binding domain 2 | 0.59878389 | 0.008453 |
| ENSSSCT00000043865.1 | high mobility group nucleosomal binding domain 2 | 0.600236418 | 0.00454 |
| ENSSSCT00000004990.3 | spindle and kinetochore associated complex subunit 1 | 0.604340723 | 0.029291 |
| ENSSSCT00000006941.3 | NUF2, NDC80 kinetochore complex component | 0.609798075 | 0.020409 |
| ENSSSCT00000043788.1 | baculoviral IAP repeat containing 5 | 0.614032008 | 0.014108 |
| ENSSSCT00000025604.2 | aurora kinase B | 0.624598385 | 0.045966 |
| ENSSSCT00000031972.2 | defensin beta 1 | 0.624929729 | 0.037845 |
| ENSSSCT00000017118.3 | chromosome 2 open reading frame 76 | 0.628226461 | 0.006941 |
| ENSSSCT00000011180.3 | cyclin dependent kinase 1 | 0.637482404 | 0.001453 |
| ENSSSCT00000017188.3 | centromere protein U | 0.638877775 | 0.045603 |
| ENSSSCT00000017172.2 | nei like DNA glycosylase 3 | 0.639388399 | 0.037839 |
| ENSSSCT00000050519.1 | gremlin 1, DAN family BMP antagonist | 0.649166447 | 0.017432 |
| ENSSSCT00000012281.3 | 3-oxoacyl-ACP synthase, mitochondrial | 0.650664018 | 0.043202 |
| ENSSSCT00000023188.3 | eukaryotic translation initiation factor 2 subunit gamma | 0.654125643 | 0.013903 |
| ENSSSCT00000010449.3 | testis expressed 30 | 0.657127062 | 0.007015 |
| ENSSSCT00000006754.3 | stathmin 2 | 0.665359494 | 0.012709 |
| ENSSSCT00000006347.3 | haloacid dehalogenase like hydrolase domain containing 2 | 0.670161395 | 0.00572 |
| ENSSSCT00000064097.1 | transforming growth factor beta receptor 1 | 0.67343119 | 0.020921 |
| ENSSSCT00000011604.3 | SWI5 dependent homologous recombination repair protein 1 | 0.680234644 | 0.029875 |
| ENSSSCT00000005524.3 | DNA polymerase epsilon 2, accessory subunit | 0.6854355 | 0.038333 |
| ENSSSCT00000052266.1 | eukaryotic translation initiation factor 3 subunit J | 0.685624346 | 0.04602 |
| ENSSSCT00000058369.1 | SPC24, NDC80 kinetochore complex component | 0.685643523 | 0.026832 |
| ENSSSCT00000012916.3 | replication factor C subunit 4 | 0.685823694 | 0.00218 |
| ENSSSCT00000063059.1 | derlin 3 | 0.68816312 | 0.040705 |
| ENSSSCT00000043965.1 | defective in cullin neddylation 1 domain containing 5 | 0.717238488 | 0.011704 |
| ENSSSCT00000053668.1 | serine and arginine rich splicing factor 4 | 0.723649936 | 0.033255 |
| ENSSSCT00000012791.3 | procollagen C-endopeptidase enhancer 2 | 0.731602288 | 0.028648 |
| ENSSSCT00000064385.1 | C-X-C motif chemokine receptor 4 | 0.733653518 | 0.02282 |
| ENSSSCT00000047826.1 | WD repeat domain 41 | 0.745785671 | 0.018792 |
| ENSSSCT00000015309.3 | MRN complex interacting protein | 0.766799694 | 0.025063 |
| ENSSSCT00000060063.1 | solute carrier family 5 member 10 | 0.768063884 | 0.041648 |
| ENSSSCT00000047728.1 | replication factor C subunit 2 | 0.769814736 | 0.023488 |
| ENSSSCT00000035788.2 | S100 calcium binding protein A1 | 0.770154106 | 0.014967 |
| ENSSSCT00000012996.3 | ELL associated factor 2 | 0.773903472 | 0.029395 |
| ENSSSCT00000046112.1 | CDC42 small effector 2 | 0.788950942 | 0.021176 |
| ENSSSCT00000009928.3 | mitochondria localized glutamic acid rich protein | 0.790021424 | 0.038566 |
| ENSSSCT00000033982.2 | intercellular adhesion molecule 3 | 0.823172364 | 0.020185 |
| ENSSSCT00000016153.3 | nuclear mitotic apparatus protein 1 | 0.834072444 | 0.047622 |
| ENSSSCT00000014565.3 | gamma-butyrobetaine hydroxylase 1 | 0.872893532 | 0.033622 |
| ENSSSCT00000043338.1 | versican | 0.87513796 | 0.025294 |
| ENSSSCT00000046422.1 | S1 RNA binding domain 1 | 0.890219226 | 0.022413 |
| ENSSSCT00000016226.3 | alkaline ceramidase 3 | 0.90691549 | 0.012298 |
| ENSSSCT00000052551.1 | solute carrier family 39 member 10 | 0.928202895 | 0.012258 |
| ENSSSCT00000025069.2 | limb and CNS expressed 1 | 0.949544016 | 0.01143 |
| ENSSSCT00000048761.1 | chitinase 3 like 1 | 0.960948058 | 0.030693 |
| ENSSSCT00000012844.3 | intraflagellar transport 80 | 0.969833022 | 0.047623 |
| ENSSSCT00000047882.1 | clusterin | 1.005010783 | 0.020831 |
| ENSSSCT00000046103.1 | MPV17, mitochondrial inner membrane protein | 1.013338867 | 0.045797 |
| ENSSSCT00000019289.2 | chemokine (C-C motif) ligand 8 | 1.016534039 | 0.045997 |
| ENSSSCT00000011038.3 | immunoglobulin lambda-like polypeptide 5 | 1.036474556 | 0.026357 |
| ENSSSCT00000019506.4 | arachidonate 15-lipoxygenase | 1.109181991 | 0.033919 |
| ENSSSCT00000059163.1 |  | 1.126081509 | 0.007627 |
| ENSSSCT00000017304.3 | proteasome 26S subunit, non-ATPase 14 | 1.168366775 | 0.02887 |
| ENSSSCT00000004692.3 | family with sequence similarity 184 member A | 1.20408823 | 0.029687 |
| ENSSSCT00000007022.4 | mucosal pentraxin | 1.206167407 | 0.016579 |
| ENSSSCT00000027731.2 | mannose phosphate isomerase | 1.23492679 | 0.043039 |
| ENSSSCT00000062488.1 | PMS1 homolog 1, mismatch repair system component | 1.235166181 | 0.030445 |
| ENSSSCT00000023341.2 | armadillo repeat containing 10 | 1.247727585 | 0.006656 |
| ENSSSCT00000016571.3 | hepatic and glial cell adhesion molecule | 1.267849892 | 0.023699 |
| ENSSSCT00000041577.1 | ubiquitin specific peptidase 16 | 1.294829056 | 0.045878 |
| ENSSSCT00000041167.1 | SP110 nuclear body protein | 1.380646678 | 0.009846 |
| ENSSSCT00000042803.1 | CCR4-NOT transcription complex subunit 7 | 1.419083339 | 0.043427 |
| ENSSSCT00000006793.3 | chromosome 8 open reading frame 34 | 1.426807817 | 0.025024 |
| ENSSSCT00000016304.3 | centrosomal protein 295 | 1.45235635 | 0.041128 |
| ENSSSCT00000040280.1 | chromosome 18 open reading frame 54 | 1.481444432 | 0.036926 |
| ENSSSCT00000048246.1 | FA complementation group D2 | 1.547747781 | 0.033771 |
| ENSSSCT00000039530.1 | brain expressed X-linked 4 | 1.560075754 | 0.033499 |
| ENSSSCT00000057530.1 | TPX2, microtubule nucleation factor | 1.653623704 | 0.037522 |
| ENSSSCT00000065027.1 | VPS33B, late endosome and lysosome associated | 1.765004647 | 0.030336 |
| ENSSSCT00000028858.2 | apoptotic peptidase activating factor 1 | 1.954412716 | 0.031915 |
| ENSSSCT00000033999.2 |  | 1.969757262 | 0.00081 |
| ENSSSCT00000029423.2 |  | 1.991597721 | 0.01367 |
| ENSSSCT00000023585.2 | paired immunoglobulin-like type 2 receptor beta | 2.023816479 | 0.017532 |
| ENSSSCT00000027492.2 | ADP ribosylation factor GTPase activating protein 2 | 2.054317761 | 0.03105 |
| ENSSSCT00000049905.1 | cysteine rich protein 3 | 2.056733742 | 0.043518 |
| ENSSSCT00000016464.4 | C-X-C motif chemokine receptor 5 | 2.362998357 | 0.047759 |
| ENSSSCT00000038494.1 | Ras association domain family member 4 | 2.411875599 | 0.005498 |
| ENSSSCT00000063614.1 | oviductal glycoprotein 1 | 2.42784113 | 0.017643 |
| ENSSSCT00000045699.1 | myocyte enhancer factor 2C | 2.640831008 | 0.016793 |
| ENSSSCT00000039185.1 | kelch like family member 24 | 2.725006501 | 0.043902 |
| ENSSSCT00000010388.3 | SLAIN motif family member 1 | 2.781303458 | 0.028404 |
| ENSSSCT00000057511.1 | ATPase family, AAA domain containing 5 | 2.871883618 | 0.047853 |
| ENSSSCT00000038407.1 | ATPase family, AAA domain containing 5 | 2.882508042 | 0.005643 |
| ENSSSCT00000026455.2 | transmembrane protein 209 | 3.052941215 | 0.031125 |
| ENSSSCT00000023948.2 | katanin catalytic subunit A1 | 3.125810092 | 0.00686 |
| ENSSSCT00000042090.1 | caspase 3 | 3.370298608 | 0.027634 |
| ENSSSCT00000005281.3 | BUB1 mitotic checkpoint serine/threonine kinase B | 3.401425176 | 0.007037 |
| ENSSSCT00000047161.1 | transducin beta like 1 X-linked receptor 1 | 4.017776541 | 0.010091 |
| ENSSSCT00000046735.1 |  | 4.980405365 | 0.02181 |

**Supplementary Table 4. Genes related to transporter activity**

| **ID** | **Log2 fold change** | **p-value** | **Gene Name** | **Panther Family** |
| --- | --- | --- | --- | --- |
| **ZnR vs Zn100** | | | | |
| ENSSSCT00000042792 | -2.3890 | 0.0245 | Phosphatidylinositol transfer protein membrane associated 2;PITPNM2;ortholog | MEMBRANE-ASSOCIATED PHOSPHATIDYLINOSITOL TRANSFER PROTEIN 2 (PTHR10658:SF41) |
| ENSSSCT00000018784 | -1.5407 | 0.0473 | ATP binding cassette subfamily A member 5;ABCA5;ortholog | ATP-BINDING CASSETTE SUB-FAMILY A MEMBER 5 (PTHR19229:SF100) |
| ENSSSCT00000062766 | -1.0985 | 0.0096 | Phospholipid-transporting ATPase;ATP11B;ortholog | PHOSPHOLIPID-TRANSPORTING ATPASE IF-RELATED (PTHR24092:SF57) |
| ENSSSCT00000041445 | -0.6308 | 0.0231 | Leukocyte receptor cluster member 8;LENG8;ortholog | LEUKOCYTE RECEPTOR CLUSTER MEMBER 8 (PTHR12436:SF4) |
| ENSSSCT00000025107 | -0.5828 | 0.0099 | Solute carrier family 2 member 6;SLC2A6;ortholog | SOLUTE CARRIER FAMILY 2, FACILITATED GLUCOSE TRANSPORTER MEMBER 6 (PTHR23500:SF111) |
| ENSSSCT00000056853 | 0.1994 | 0.0112 | ATP synthase subunit alpha, mitochondrial;ATP5F1A;ortholog | ATP SYNTHASE SUBUNIT ALPHA, MITOCHONDRIAL (PTHR43089:SF2) |
| ENSSSCT00000006485 | 1.8504 | 0.0000 | Solute carrier family 39 member 4;SLC39A4;ortholog | ZINC TRANSPORTER ZIP4 (PTHR12191:SF21) |
|  |  |  |  |  |
| **ZnR vs Zn50** | | | | |
| ENSSSCT00000049539 | -2.1120 | 0.0003 | Uncharacterized protein;CLCA4;ortholog | CALCIUM-ACTIVATED CHLORIDE CHANNEL REGULATOR 4 (PTHR10579:SF2) |
| ENSSSCT00000014039 | -1.7266 | 0.0323 | Solute carrier family 25 member 22;SLC25A22;ortholog | MITOCHONDRIAL GLUTAMATE CARRIER 1 (PTHR45678:SF3) |
| ENSSSCT00000050410 | -1.7110 | 0.0390 | Uncharacterized protein;unassigned;ortholog | MULTIDRUG RESISTANCE-ASSOCIATED PROTEIN 4 (PTHR24223:SF357) |
| ENSSSCT00000051531 | -1.4259 | 0.0156 | Uncharacterized protein;LOC100738425;ortholog | SUBFAMILY NOT NAMED (PTHR24223:SF389) |
| ENSSSCT00000022953 | -1.0106 | 0.0166 | Transient receptor potential cation channel subfamily M member 4;TRPM4;ortholog | TRANSIENT RECEPTOR POTENTIAL CATION CHANNEL SUBFAMILY M MEMBER 4 (PTHR13800:SF6) |
| ENSSSCT00000010052 | -0.7526 | 0.0374 | Microsomal triglyceride transfer protein large subunit;MTTP;ortholog | MICROSOMAL TRIGLYCERIDE TRANSFER PROTEIN LARGE SUBUNIT (PTHR13024:SF1) |
| ENSSSCT00000018638 | -0.6577 | 0.0378 | Transporter;SLC6A19;ortholog | SODIUM-DEPENDENT NEUTRAL AMINO ACID TRANSPORTER B(0)AT1 (PTHR11616:SF125) |
| ENSSSCT00000033364 | -0.6371 | 0.0075 | Choline transporter-like protein 4;SLC44A4;ortholog | CHOLINE TRANSPORTER-LIKE PROTEIN 4 (PTHR12385:SF37) |
| ENSSSCT00000003627 | -0.5967 | 0.0205 | Transmembrane channel-like protein;TMC4;ortholog | TRANSMEMBRANE CHANNEL-LIKE PROTEIN 4 (PTHR23302:SF45) |
| ENSSSCT00000007385 | -0.5951 | 0.0318 | Sodium/potassium-transporting ATPase subunit alpha;ATP1A1;ortholog | SODIUM/POTASSIUM-TRANSPORTING ATPASE SUBUNIT ALPHA-1 (PTHR43294:SF9) |
| ENSSSCT00000037119 | -0.5907 | 0.0090 | Uncharacterized protein;SLC25A10;ortholog | MITOCHONDRIAL DICARBOXYLATE CARRIER-RELATED (PTHR45618:SF13) |
| ENSSSCT00000030079 | -0.5743 | 0.0499 | Transporter;SLC6A20;ortholog | SODIUM- AND CHLORIDE-DEPENDENT TRANSPORTER XTRP3 (PTHR11616:SF44) |
| ENSSSCT00000025910 | -0.5721 | 0.0192 | Solute carrier family 23 member 1;SLC23A1;ortholog | SOLUTE CARRIER FAMILY 23 MEMBER 1 (PTHR11119:SF21) |
| ENSSSCT00000041445 | -0.5622 | 0.0316 | Leukocyte receptor cluster member 8;LENG8;ortholog | LEUKOCYTE RECEPTOR CLUSTER MEMBER 8 (PTHR12436:SF4) |
| ENSSSCT00000001629 | -0.4911 | 0.0397 | Antigen peptide transporter 2;TAP2;ortholog | ANTIGEN PEPTIDE TRANSPORTER 2 (PTHR24221:SF237) |
| ENSSSCT00000022886 | -0.2748 | 0.0492 | Phosphatidylinositol transfer protein alpha;PITPNA;ortholog | PHOSPHATIDYLINOSITOL TRANSFER PROTEIN ALPHA ISOFORM (PTHR10658:SF28) |
| ENSSSCT00000018047 | 0.2381 | 0.0283 | V-type proton ATPase subunit F;ATP6V1F;ortholog | V-TYPE PROTON ATPASE SUBUNIT F (PTHR13861:SF2) |
| ENSSSCT00000016235 | 0.2862 | 0.0379 | Chloride nucleotide-sensitive channel 1A;CLNS1A;ortholog | METHYLOSOME SUBUNIT PICLN (PTHR21399:SF0) |
| ENSSSCT00000033835 | 0.2881 | 0.0438 | Solute carrier family 44 member 1;SLC44A1;ortholog | CHOLINE TRANSPORTER-LIKE PROTEIN 1 (PTHR12385:SF56) |
| ENSSSCT00000041673 | 0.3057 | 0.0379 | Mitochondrial import inner membrane translocase subunit TIM23;LOC100157391;ortholog | MITOCHONDRIAL IMPORT INNER MEMBRANE TRANSLOCASE SUBUNIT TIM23-RELATED (PTHR15371:SF0) |
| ENSSSCT00000038200 | 0.3621 | 0.0120 | Uncharacterized protein;unassigned;ortholog | ATP SYNTHASE SUBUNIT G 2, MITOCHONDRIAL-RELATED (PTHR12386:SF29) |
| ENSSSCT00000047000 | 0.5582 | 0.0452 | ATP synthase F(0) complex subunit C3, mitochondrial;ATP5MC3;ortholog | ATP SYNTHASE F(0) COMPLEX SUBUNIT C1, MITOCHONDRIAL (PTHR10031:SF31) |
| ENSSSCT00000052551 | 0.9282 | 0.0123 | Solute carrier family 39 member 10;SLC39A10;ortholog | ZINC TRANSPORTER ZIP10 (PTHR12191:SF14) |
|  |  |  |  |  |
| **Zn100 vs Zn50** | | | | |
| ENSSSCT00000010090 | -3.2205 | 0.0017 | Uncharacterized protein;LOC110255210;ortholog | ATP-BINDING CASSETTE SUB-FAMILY G MEMBER 2 (PTHR19241:SF311) |
| ENSSSCT00000049539 | -2.0952 | 0.0008 | Uncharacterized protein;CLCA4;ortholog | CALCIUM-ACTIVATED CHLORIDE CHANNEL REGULATOR 4 (PTHR10579:SF2) |
| ENSSSCT00000061852 | -1.3456 | 0.0409 | Apolipoprotein A-IV;APOA4;ortholog | APOLIPOPROTEIN A-IV (PTHR18976:SF1) |
| ENSSSCT00000006485 | -1.2544 | 0.0001 | Solute carrier family 39 member 4;SLC39A4;ortholog | ZINC TRANSPORTER ZIP4 (PTHR12191:SF21) |
| ENSSSCT00000017665 | -0.7795 | 0.0282 | Long-chain-fatty-acid--CoA ligase 3;ACSL3;ortholog | LONG-CHAIN-FATTY-ACID--COA LIGASE 3 (PTHR43272:SF13) |
| ENSSSCT00000028073 | -0.6396 | 0.0254 | Oxysterol-binding protein;OSBPL9;ortholog | OXYSTEROL-BINDING PROTEIN-RELATED PROTEIN 9 (PTHR10972:SF102) |
| ENSSSCT00000057665 | -0.6319 | 0.0360 | Uncharacterized protein;unassigned;ortholog | COPPER-TRANSPORTING ATPASE 2 (PTHR43520:SF9) |
| ENSSSCT00000034970 | 0.8372 | 0.0095 | ATP-binding cassette sub-family G member 2;ABCG2;ortholog | ATP-BINDING CASSETTE SUB-FAMILY G MEMBER 2 (PTHR19241:SF311) |
| ENSSSCT00000052352 | 2.1233 | 0.0112 | Solute carrier family 25 member 22;SLC25A22;ortholog | MITOCHONDRIAL GLUTAMATE CARRIER 1 (PTHR45678:SF3) |
| ENSSSCT00000042792 | 2.1415 | 0.0386 | Phosphatidylinositol transfer protein membrane associated 2;PITPNM2;ortholog | MEMBRANE-ASSOCIATED PHOSPHATIDYLINOSITOL TRANSFER PROTEIN 2 (PTHR10658:SF41) |
|  |  |  |  |  |

**Supplementary Table 5. Genes related to binding proteins**

| **ID** | **Log2 Fold Change** | **p-value** | **Gene Name** | **Panther Family** |
| --- | --- | --- | --- | --- |
| **ZnR vs Zn100** | | | | |
| ENSSSCT00000044729 | -2.8201 | 0.0281 | Somatostatin receptor type 5;SSTR5;ortholog | SOMATOSTATIN RECEPTOR TYPE 5 (PTHR24229:SF20) |
| ENSSSCT00000032296 | -2.7795 | 0.0130 | Espin;ESPN;ortholog | ESPIN (PTHR24153:SF14) |
| ENSSSCT00000058397 | -2.7387 | 0.0076 | Metallothionein;LOC100739663;ortholog | METALLOTHIONEIN-1A (PTHR23299:SF22) |
| ENSSSCT00000047481 | -2.4079 | 0.0306 | Uncharacterized protein;EIF5;ortholog | EUKARYOTIC TRANSLATION INITIATION FACTOR 5 (PTHR23001:SF7) |
| ENSSSCT00000027420 | -2.4049 | 0.0135 | Metallothionein-3;MT3;ortholog | METALLOTHIONEIN-3 (PTHR23299:SF18) |
| ENSSSCT00000042792 | -2.3890 | 0.0245 | Phosphatidylinositol transfer protein membrane associated 2;PITPNM2;ortholog | MEMBRANE-ASSOCIATED PHOSPHATIDYLINOSITOL TRANSFER PROTEIN 2 (PTHR10658:SF41) |
| ENSSSCT00000049484 | -1.9020 | 0.0456 | Protein S100-G;S100G;ortholog | PROTEIN S100-G (PTHR11639:SF73) |
| ENSSSCT00000049323 | -1.5859 | 0.0057 | Transgelin;TAGLN2;ortholog | TRANSGELIN-2 (PTHR18959:SF41) |
| ENSSSCT00000060915 | -1.4614 | 0.0002 | Neurogranin;NRGN;ortholog | NEUROGRANIN (PTHR10699:SF18) |
| ENSSSCT00000014394 | -1.2829 | 0.0462 | Catenin delta 1;CTNND1;ortholog | CATENIN DELTA-1 (PTHR10372:SF6) |
| ENSSSCT00000058181 | -1.1787 | 0.0404 | PHD finger protein 6;PHF6;ortholog | PHD FINGER PROTEIN 6 (PTHR12420:SF15) |
| ENSSSCT00000065484 | -1.1044 | 0.0385 | Midkine precursor;MDK;ortholog | MIDKINE (PTHR13850:SF2) |
| ENSSSCT00000035898 | -1.0901 | 0.0439 | Interferon-induced protein with tetratricopeptide repeats 2;IFIT2;ortholog | INTERFERON-INDUCED PROTEIN WITH TETRATRICOPEPTIDE REPEATS 2 (PTHR10271:SF4) |
| ENSSSCT00000059743 | -1.0827 | 0.0191 | Serine and arginine repetitive matrix 1;SRRM1;ortholog | SERINE/ARGININE REPETITIVE MATRIX PROTEIN 1 (PTHR23148:SF0) |
| ENSSSCT00000064542 | -0.9575 | 0.0153 | MAP kinase-activated protein kinase 3;MAPKAPK3;ortholog | MAP KINASE-ACTIVATED PROTEIN KINASE 3 (PTHR24349:SF64) |
| ENSSSCT00000027578 | -0.8764 | 0.0425 | Kinesin-like protein;KIFC2;ortholog | KINESIN-LIKE PROTEIN KIFC2 (PTHR24115:SF479) |
| ENSSSCT00000026311 | -0.8249 | 0.0233 | Uncharacterized protein;unassigned;ortholog | HEAT SHOCK 70 KDA PROTEIN 1A-RELATED (PTHR19375:SF223) |
| ENSSSCT00000009844 | -0.6686 | 0.0143 | Cyclin I;CCNI;ortholog | CYCLIN-I (PTHR10177:SF187) |
| ENSSSCT00000042890 | -0.6305 | 0.0439 | MAF bZIP transcription factor B;MAFB;ortholog | TRANSCRIPTION FACTOR MAFB (PTHR10129:SF10) |
| ENSSSCT00000061170 | -0.4700 | 0.0128 | Serine/arginine-rich splicing factor 2;SRSF2;ortholog | SERINE/ARGININE-RICH SPLICING FACTOR 2 (PTHR23147:SF17) |
| ENSSSCT00000003711 | -0.4623 | 0.0345 | Dishevelled segment polarity protein 1;DVL1;ortholog | SEGMENT POLARITY PROTEIN DISHEVELLED HOMOLOG DVL-1-RELATED (PTHR10878:SF5) |
| ENSSSCT00000045167 | -0.2882 | 0.0415 | Toll-interacting protein;TOLLIP;ortholog | TOLL-INTERACTING PROTEIN (PTHR16461:SF5) |
| ENSSSCT00000017493 | -0.2217 | 0.0270 | Serine/threonine kinase 17b;STK17B;ortholog | SERINE/THREONINE-PROTEIN KINASE 17B (PTHR24342:SF5) |
| ENSSSCT00000018727 | 0.1734 | 0.0309 | Histone H3.3;H3F3A;ortholog | HISTONE H3.3-RELATED (PTHR11426:SF179) |
| ENSSSCT00000045474 | 0.1987 | 0.0366 | Ribosomal protein;RPL10A;ortholog | 60S RIBOSOMAL PROTEIN L10A (PTHR23105:SF107) |
| ENSSSCT00000056853 | 0.1994 | 0.0112 | ATP synthase subunit alpha, mitochondrial;ATP5F1A;ortholog | ATP SYNTHASE SUBUNIT ALPHA, MITOCHONDRIAL (PTHR43089:SF2) |
| ENSSSCT00000002569 | 0.2227 | 0.0429 | Enhancer of rudimentary homolog;ERH;ortholog | ENHANCER OF RUDIMENTARY HOMOLOG (PTHR12373:SF0) |
| ENSSSCT00000011019 | 0.2289 | 0.0285 | Small nuclear ribonucleoprotein Sm D3;SNRPD3;ortholog | SMALL NUCLEAR RIBONUCLEOPROTEIN SM D3 (PTHR23338:SF17) |
| ENSSSCT00000053256 | 0.2441 | 0.0465 | 60S ribosomal protein L6;RPL6;ortholog | 60S RIBOSOMAL PROTEIN L6 (PTHR10715:SF0) |
| ENSSSCT00000029514 | 0.2492 | 0.0421 | Superoxide dismutase [Cu-Zn];SOD1;ortholog | SUPEROXIDE DISMUTASE [CU-ZN] (PTHR10003:SF58) |
| ENSSSCT00000057837 | 0.2711 | 0.0367 | DNA polymerase epsilon 4, accessory subunit;POLE4;ortholog | DNA POLYMERASE EPSILON SUBUNIT 4 (PTHR10252:SF79) |
| ENSSSCT00000044665 | 0.2862 | 0.0271 | Mitochondrial complement component 1 Q subcomponent-binding protein;C1QBP;ortholog | COMPLEMENT COMPONENT 1 Q SUBCOMPONENT-BINDING PROTEIN, MITOCHONDRIAL (PTHR10826:SF1) |
| ENSSSCT00000022560 | 0.2906 | 0.0333 | RNA polymerase II subunit G;POLR2G;ortholog | DNA-DIRECTED RNA POLYMERASE II SUBUNIT RPB7 (PTHR12709:SF4) |
| ENSSSCT00000017506 | 0.2973 | 0.0234 | 10 kDa heat shock protein, mitochondrial;HSPE1;ortholog | 10 KDA HEAT SHOCK PROTEIN, MITOCHONDRIAL (PTHR10772:SF0) |
| ENSSSCT00000060340 | 0.3039 | 0.0324 | Acyl-CoA-binding protein;DBI;ortholog | ACYL-COA-BINDING PROTEIN (PTHR23310:SF54) |
| ENSSSCT00000005151 | 0.3193 | 0.0314 | Sulfide:quinone oxidoreductase, mitochondrial;SQOR;ortholog | SULFIDE:QUINONE OXIDOREDUCTASE, MITOCHONDRIAL (PTHR10632:SF2) |
| ENSSSCT00000013014 | 0.3204 | 0.0237 | Cytochrome c oxidase copper chaperone;COX17;ortholog | CYTOCHROME C OXIDASE COPPER CHAPERONE (PTHR16719:SF0) |
| ENSSSCT00000046956 | 0.3280 | 0.0106 | Peptidyl-prolyl cis-trans isomerase;PPIB;ortholog | PEPTIDYL-PROLYL CIS-TRANS ISOMERASE B (PTHR11071:SF387) |
| ENSSSCT00000004100 | 0.3294 | 0.0273 | Small nuclear ribonucleoprotein D1 polypeptide;SNRPD1;ortholog | SMALL NUCLEAR RIBONUCLEOPROTEIN SM D1 (PTHR23338:SF18) |
| ENSSSCT00000010255 | 0.3350 | 0.0201 | Exosome component 8;EXOSC8;ortholog | EXOSOME COMPLEX COMPONENT RRP43 (PTHR11097:SF9) |
| ENSSSCT00000010764 | 0.3395 | 0.0103 | Actin-related protein 2/3 complex subunit 3;ARPC3;ortholog | ACTIN-RELATED PROTEIN 2/3 COMPLEX SUBUNIT 3 (PTHR12391:SF1) |
| ENSSSCT00000000089 | 0.3463 | 0.0049 | Ribosomal protein S19 binding protein 1;RPS19BP1;ortholog | ACTIVE REGULATOR OF SIRT1 (PTHR31454:SF2) |
| ENSSSCT00000053892 | 0.3595 | 0.0415 | ADP-ribosylation factor 1;ARF1;ortholog | ADP-RIBOSYLATION FACTOR 1 (PTHR11711:SF350) |
| ENSSSCT00000031595 | 0.3732 | 0.0270 | Uncharacterized protein;SNRPG;ortholog | SMALL NUCLEAR RIBONUCLEOPROTEIN G (PTHR10553:SF2) |
| ENSSSCT00000034332 | 0.3873 | 0.0366 | 39S ribosomal protein L13, mitochondrial;MRPL13;ortholog | 39S RIBOSOMAL PROTEIN L13, MITOCHONDRIAL (PTHR11545:SF17) |
| ENSSSCT00000000979 | 0.4076 | 0.0287 | Small nuclear ribonucleoprotein F;SNRPF;ortholog | SMALL NUCLEAR RIBONUCLEOPROTEIN F (PTHR11021:SF5) |
| ENSSSCT00000031620 | 0.4100 | 0.0009 | SRA stem-loop-interacting RNA-binding protein, mitochondrial;SLIRP;ortholog | SRA STEM-LOOP-INTERACTING RNA-BINDING PROTEIN, MITOCHONDRIAL (PTHR15241:SF131) |
| ENSSSCT00000025596 | 0.4149 | 0.0316 | Cyclin-dependent kinases regulatory subunit;CKS2;ortholog | CYCLIN-DEPENDENT KINASES REGULATORY SUBUNIT 2 (PTHR23415:SF35) |
| ENSSSCT00000055436 | 0.4451 | 0.0352 | Interferon regulatory factor 1;IRF1;ortholog | INTERFERON REGULATORY FACTOR 1 (PTHR11949:SF3) |
| ENSSSCT00000003776 | 0.4880 | 0.0413 | Centromere protein S;CENPS;ortholog | CENTROMERE PROTEIN S (PTHR22980:SF4) |
| ENSSSCT00000012916 | 0.4909 | 0.0403 | Replication factor C subunit 4;RFC4;ortholog | REPLICATION FACTOR C SUBUNIT 4 (PTHR11669:SF20) |
| ENSSSCT00000014614 | 0.5262 | 0.0314 | Uncharacterized protein;NUCB2;ortholog | NUCLEOBINDIN-2 (PTHR19237:SF22) |
| ENSSSCT00000023171 | 0.6438 | 0.0461 | NHP2-like protein 1;SNU13;ortholog | NHP2-LIKE PROTEIN 1 (PTHR23105:SF38) |
| ENSSSCT00000027887 | 0.7207 | 0.0371 | Ficolin-2;FCN2;ortholog | FICOLIN-1-RELATED (PTHR19143:SF346) |
| ENSSSCT00000038540 | 0.7231 | 0.0348 | Uncharacterized protein;unassigned;ortholog | IMMUNOGLOBULIN HEAVY VARIABLE 3-11-RELATED (PTHR23266:SF204) |
| ENSSSCT00000010221 | 0.9548 | 0.0191 | High mobility group protein B1;HMGB1;ortholog | SUBFAMILY NOT NAMED (PTHR13711:SF328) |
| ENSSSCT00000000461 | 1.0827 | 0.0202 | Signal transducer and activator of transcription;STAT6;ortholog | SIGNAL TRANSDUCER AND ACTIVATOR OF TRANSCRIPTION 6 (PTHR11801:SF48) |
| ENSSSCT00000007022 | 1.1391 | 0.0399 | Pentaxin;LOC100517891;ortholog | MUCOSAL PENTRAXIN-RELATED (PTHR45869:SF6) |
| ENSSSCT00000053543 | 1.3745 | 0.0077 | Chemokine C-C motif receptor-like 2;CCRL2;ortholog | C-C CHEMOKINE RECEPTOR-LIKE 2 (PTHR10489:SF655) |
| ENSSSCT00000043331 | 1.4202 | 0.0017 | TATA-box binding protein associated factor 9;TAF9;ortholog | TRANSCRIPTION INITIATION FACTOR TFIID SUBUNIT 9 (PTHR12595:SF8) |
| ENSSSCT00000016571 | 1.4630 | 0.0130 | Hepatic and glial cell adhesion molecule;HEPACAM;ortholog | HEPATOCYTE CELL ADHESION MOLECULE (PTHR12080:SF59) |
| ENSSSCT00000029033 | 1.6091 | 0.0202 | Synaptotagmin 12;SYT12;ortholog | SYNAPTOTAGMIN-12 (PTHR10024:SF252) |
| ENSSSCT00000017304 | 1.8392 | 0.0065 | Proteasome 26S subunit, non-ATPase 14;PSMD14;ortholog | 26S PROTEASOME NON-ATPASE REGULATORY SUBUNIT 14 (PTHR10410:SF5) |
| ENSSSCT00000045699 | 1.9738 | 0.0499 | Myocyte-specific enhancer factor 2C;MEF2C;ortholog | MYOCYTE-SPECIFIC ENHANCER FACTOR 2C (PTHR11945:SF25) |
| ENSSSCT00000022990 | 2.0309 | 0.0194 | Histone H2A;LOC110261483;ortholog | HISTONE H2A TYPE 1-D (PTHR23430:SF196) |
| ENSSSCT00000052291 | 2.3122 | 0.0093 | Centromere protein O;CENPO;ortholog | CENTROMERE PROTEIN O (PTHR14582:SF1) |
| ENSSSCT00000010533 | 2.3770 | 0.0462 | Nucleoplasmin-2;NPM2;ortholog | NUCLEOPLASMIN-2 (PTHR22747:SF14) |
|  |  |  |  |  |
| **ZnR vs Zn50** | | | | |
| ENSSSCT00000046592 | -3.6290 | 0.0481 | RBR-type E3 ubiquitin transferase;RNF144A;ortholog | E3 UBIQUITIN-PROTEIN LIGASE RNF144A (PTHR11685:SF99) |
| ENSSSCT00000014068 | -3.3340 | 0.0458 | Fibroblast growth factor;FGF19;ortholog | FIBROBLAST GROWTH FACTOR 19 (PTHR11486:SF74) |
| ENSSSCT00000062411 | -2.4678 | 0.0260 | Transposase;unassigned;ortholog | LINE-1 RETROTRANSPOSABLE ELEMENT ORF1 PROTEIN (PTHR11505:SF195) |
| ENSSSCT00000035898 | -1.7327 | 0.0124 | Interferon-induced protein with tetratricopeptide repeats 2;IFIT2;ortholog | INTERFERON-INDUCED PROTEIN WITH TETRATRICOPEPTIDE REPEATS 2 (PTHR10271:SF4) |
| ENSSSCT00000005664 | -1.6456 | 0.0229 | Interferon epsilon;IFNE;ortholog | INTERFERON EPSILON (PTHR11691:SF8) |
| ENSSSCT00000060915 | -1.4797 | 0.0260 | Neurogranin;NRGN;ortholog | NEUROGRANIN (PTHR10699:SF18) |
| ENSSSCT00000026311 | -1.4535 | 0.0001 | Uncharacterized protein;unassigned;ortholog | HEAT SHOCK 70 KDA PROTEIN 1A-RELATED (PTHR19375:SF223) |
| ENSSSCT00000022686 | -1.4339 | 0.0119 | Cytochrome P450 2C42;CYP2C42;ortholog | CYTOCHROME P450 2C18 (PTHR24300:SF292) |
| ENSSSCT00000049323 | -1.3872 | 0.0395 | Transgelin;TAGLN2;ortholog | TRANSGELIN-2 (PTHR18959:SF41) |
| ENSSSCT00000053274 | -1.0283 | 0.0209 | Tropomyosin beta chain;TPM2;ortholog | TROPOMYOSIN BETA CHAIN (PTHR19269:SF46) |
| ENSSSCT00000056748 | -0.9711 | 0.0058 | Galectin;LGALS9;ortholog | GALECTIN-9 (PTHR11346:SF80) |
| ENSSSCT00000055212 | -0.9512 | 0.0290 | Interferon-induced GTP-binding protein Mx2;MX2;ortholog | INTERFERON-INDUCED GTP-BINDING PROTEIN MX2 (PTHR11566:SF46) |
| ENSSSCT00000040690 | -0.9355 | 0.0331 | Anti-apoptotic regulator Bcl-xL;bcl-xL;ortholog | BCL-2-LIKE PROTEIN 1 (PTHR11256:SF12) |
| ENSSSCT00000031440 | -0.9122 | 0.0475 | RAS like proto-oncogene B;RALB;ortholog | RAS-RELATED PROTEIN RAL-B (PTHR24070:SF199) |
| ENSSSCT00000042890 | -0.8785 | 0.0022 | MAF bZIP transcription factor B;MAFB;ortholog | TRANSCRIPTION FACTOR MAFB (PTHR10129:SF10) |
| ENSSSCT00000002141 | -0.8766 | 0.0225 | Promyelocytic leukemia;PML;ortholog | PROTEIN PML (PTHR25462:SF241) |
| ENSSSCT00000052285 | -0.8527 | 0.0190 | Retinoic acid receptor RXR-alpha;RXRA;ortholog | RETINOIC ACID RECEPTOR RXR-ALPHA (PTHR24083:SF39) |
| ENSSSCT00000050048 | -0.8508 | 0.0349 | Uncharacterized protein;MOB3B;ortholog | MOB KINASE ACTIVATOR 3B (PTHR22599:SF22) |
| ENSSSCT00000019093 | -0.8246 | 0.0257 | Homeobox B6;HOXB6;ortholog | HOMEOBOX PROTEIN HOX-B6 (PTHR45659:SF9) |
| ENSSSCT00000006664 | -0.8200 | 0.0108 | Odd-skipped related transciption factor 2;OSR2;ortholog | PROTEIN ODD-SKIPPED-RELATED 2 (PTHR14196:SF4) |
| ENSSSCT00000045379 | -0.8086 | 0.0252 | Eukaryotic translation initiation factor 4 gamma 3;EIF4G3;ortholog | EUKARYOTIC TRANSLATION INITIATION FACTOR 4 GAMMA 3 (PTHR23253:SF23) |
| ENSSSCT00000066126 | -0.7982 | 0.0128 | Adseverin;SCIN;ortholog | ADSEVERIN (PTHR11977:SF78) |
| ENSSSCT00000002650 | -0.7931 | 0.0259 | Proto-oncogene c-Fos;FOS;ortholog | PROTO-ONCOGENE C-FOS (PTHR23351:SF4) |
| ENSSSCT00000014648 | -0.7884 | 0.0450 | ADM;ADM;ortholog | ADM (PTHR23414:SF3) |
| ENSSSCT00000001722 | -0.7747 | 0.0405 | Transcriptional enhancer factor TEF-5;TEAD3;ortholog | TRANSCRIPTIONAL ENHANCER FACTOR TEF-5 (PTHR11834:SF0) |
| ENSSSCT00000060403 | -0.7709 | 0.0169 | Replication initiator 1;REPIN1;ortholog | ZINC FINGER PROTEIN GLI4 (PTHR24388:SF74) |
| ENSSSCT00000062592 | -0.7382 | 0.0481 | Signal transducer and activator of transcription;stat5B;ortholog | SIGNAL TRANSDUCER AND ACTIVATOR OF TRANSCRIPTION 5B (PTHR11801:SF39) |
| ENSSSCT00000054120 | -0.7267 | 0.0045 | LLGL2, scribble cell polarity complex component;LLGL2;ortholog | LETHAL(2) GIANT LARVAE PROTEIN HOMOLOG 2 (PTHR10241:SF20) |
| ENSSSCT00000008270 | -0.7138 | 0.0054 | GATA binding protein 5;GATA5;ortholog | TRANSCRIPTION FACTOR GATA-5 (PTHR10071:SF289) |
| ENSSSCT00000003272 | -0.7059 | 0.0279 | Signal induced proliferation associated 1 like 3;SIPA1L3;ortholog | SIGNAL-INDUCED PROLIFERATION-ASSOCIATED 1-LIKE PROTEIN 3 (PTHR15711:SF15) |
| ENSSSCT00000012494 | -0.7022 | 0.0024 | CISH;CISH_tv2;ortholog | CYTOKINE-INDUCIBLE SH2-CONTAINING PROTEIN (PTHR10155:SF9) |
| ENSSSCT00000018974 | -0.6978 | 0.0030 | Junction plakoglobin;JUP;ortholog | JUNCTION PLAKOGLOBIN (PTHR45976:SF3) |
| ENSSSCT00000026262 | -0.6950 | 0.0177 | DEAQ-box RNA dependent ATPase 1;DQX1;ortholog | ATP-DEPENDENT RNA HELICASE DQX1 (PTHR18934:SF108) |
| ENSSSCT00000017903 | -0.6840 | 0.0042 | Amiloride-sensitive amine oxidase [copper-containing];AOC1;ortholog | AMILORIDE-SENSITIVE AMINE OXIDASE [COPPER-CONTAINING] (PTHR10638:SF3) |
| ENSSSCT00000048990 | -0.6839 | 0.0148 | Myosin ID;MYO1D;ortholog | UNCONVENTIONAL MYOSIN-ID (PTHR13140:SF417) |
| ENSSSCT00000014043 | -0.6836 | 0.0109 | EPS8 like 2;EPS8L2;ortholog | EPIDERMAL GROWTH FACTOR RECEPTOR KINASE SUBSTRATE 8-LIKE PROTEIN 2 (PTHR12287:SF20) |
| ENSSSCT00000035232 | -0.6798 | 0.0328 | Interferon regulatory factor 7;Irf7;ortholog | INTERFERON REGULATORY FACTOR 7 (PTHR11949:SF2) |
| ENSSSCT00000035626 | -0.6732 | 0.0087 | Aminopeptidase;ANPEP;ortholog | AMINOPEPTIDASE N (PTHR11533:SF172) |
| ENSSSCT00000008291 | -0.6649 | 0.0080 | MAF bZIP transcription factor K;MAFK;ortholog | TRANSCRIPTION FACTOR MAFK (PTHR10129:SF26) |
| ENSSSCT00000017637 | -0.6543 | 0.0250 | Villin-1;VIL1;ortholog | VILLIN-1 (PTHR11977:SF35) |
| ENSSSCT00000014247 | -0.6532 | 0.0097 | Phosphoinositide phospholipase C;PLCB3;ortholog | 1-PHOSPHATIDYLINOSITOL 4,5-BISPHOSPHATE PHOSPHODIESTERASE BETA-3 (PTHR10336:SF11) |
| ENSSSCT00000010207 | -0.6429 | 0.0055 | Homeobox protein CDX-2;CDX2;ortholog | HOMEOBOX PROTEIN CDX-2 (PTHR24332:SF27) |
| ENSSSCT00000027155 | -0.6326 | 0.0417 | Guanine nucleotide-binding protein subunit alpha-11;GNA11;ortholog | GUANINE NUCLEOTIDE-BINDING PROTEIN SUBUNIT ALPHA-11 (PTHR10218:SF57) |
| ENSSSCT00000004101 | -0.6258 | 0.0303 | Transcription factor GATA-6;GATA6;ortholog | TRANSCRIPTION FACTOR GATA-6 (PTHR10071:SF23) |
| ENSSSCT00000015600 | -0.6177 | 0.0306 | Interferon regulatory factor 1;IRF1;ortholog | INTERFERON REGULATORY FACTOR 1 (PTHR11949:SF3) |
| ENSSSCT00000003711 | -0.6166 | 0.0038 | Dishevelled segment polarity protein 1;DVL1;ortholog | SEGMENT POLARITY PROTEIN DISHEVELLED HOMOLOG DVL-1-RELATED (PTHR10878:SF5) |
| ENSSSCT00000017714 | -0.6061 | 0.0134 | Integral membrane protein 2C;ITM2C;ortholog | INTEGRAL MEMBRANE PROTEIN 2C (PTHR10962:SF5) |
| ENSSSCT00000016232 | -0.6051 | 0.0117 | P21 (RAC1) activated kinase 1;PAK1;ortholog | SERINE/THREONINE-PROTEIN KINASE PAK 1 (PTHR24361:SF232) |
| ENSSSCT00000005915 | -0.6048 | 0.0256 | Coronin;CORO2A;ortholog | CORONIN-2A (PTHR10856:SF2) |
| ENSSSCT00000024524 | -0.5954 | 0.0100 | Uncharacterized protein;PPP4R1;ortholog | SERINE/THREONINE-PROTEIN PHOSPHATASE 4 REGULATORY SUBUNIT 1 (PTHR10648:SF8) |
| ENSSSCT00000042318 | -0.5431 | 0.0312 | Uncharacterized protein;RAB11FIP1;ortholog | RAB11 FAMILY-INTERACTING PROTEIN 1 (PTHR15746:SF22) |
| ENSSSCT00000045040 | -0.5427 | 0.0346 | Poly(A) RNA polymerase D5, non-canonical;PAPD5;ortholog | NON-CANONICAL POLY(A) RNA POLYMERASE PAPD5 (PTHR23092:SF49) |
| ENSSSCT00000015156 | -0.5345 | 0.0053 | Nuclear receptor subfamily 2 group F member 6;NR2F6;ortholog | NUCLEAR RECEPTOR SUBFAMILY 2 GROUP F MEMBER 6 (PTHR24083:SF44) |
| ENSSSCT00000026198 | -0.5307 | 0.0325 | RB1 inducible coiled-coil 1;RB1CC1;ortholog | RB1-INDUCIBLE COILED-COIL PROTEIN 1 (PTHR13222:SF1) |
| ENSSSCT00000008831 | -0.5303 | 0.0280 | Potassium channel tetramerization domain containing 5;KCTD5;ortholog | BTB/POZ DOMAIN-CONTAINING PROTEIN KCTD5 (PTHR14958:SF12) |
| ENSSSCT00000034737 | -0.5297 | 0.0463 | Interferon regulatory factor 6;IRF6;ortholog | INTERFERON REGULATORY FACTOR 6 (PTHR11949:SF9) |
| ENSSSCT00000015792 | -0.5196 | 0.0420 | Caudal type homeobox 1;CDX1;ortholog | HOMEOBOX PROTEIN CDX-1 (PTHR24332:SF16) |
| ENSSSCT00000027266 | -0.5149 | 0.0260 | Galectin;LGALS4;ortholog | GALECTIN-4 (PTHR11346:SF32) |
| ENSSSCT00000015666 | -0.5137 | 0.0399 | Catenin alpha 1;CTNNA1;ortholog | CATENIN ALPHA-1 (PTHR18914:SF24) |
| ENSSSCT00000029636 | -0.5056 | 0.0489 | Suppressor of cytokine signaling 1;socs1;ortholog | SUPPRESSOR OF CYTOKINE SIGNALING 1 (PTHR10155:SF4) |
| ENSSSCT00000018774 | -0.5039 | 0.0106 | CDC42 effector protein 4;CDC42EP4;ortholog | CDC42 EFFECTOR PROTEIN 4 (PTHR15344:SF14) |
| ENSSSCT00000053600 | -0.4652 | 0.0416 | Rho GTPase activating protein 17;ARHGAP17;ortholog | RHO GTPASE-ACTIVATING PROTEIN 17 (PTHR14130:SF3) |
| ENSSSCT00000026750 | -0.4504 | 0.0015 | Tristetraprolin;ZFP36;ortholog | MRNA DECAY ACTIVATOR PROTEIN ZFP36 (PTHR12547:SF122) |
| ENSSSCT00000002240 | -0.4478 | 0.0025 | Interferon regulatory factor 9;IRF9;ortholog | INTERFERON REGULATORY FACTOR 9 (PTHR11949:SF26) |
| ENSSSCT00000011309 | -0.4474 | 0.0343 | Annexin;ANXA11;ortholog | ANNEXIN A11 (PTHR10502:SF29) |
| ENSSSCT00000045280 | -0.4267 | 0.0446 | Optineurin;OPTN;ortholog | OPTINEURIN (PTHR31553:SF2) |
| ENSSSCT00000000070 | -0.3850 | 0.0150 | Aconitate hydratase, mitochondrial;ACO2;ortholog | ACONITATE HYDRATASE, MITOCHONDRIAL (PTHR43160:SF3) |
| ENSSSCT00000044391 | -0.3817 | 0.0260 | Ferritin;FTH1;ortholog | FERRITIN HEAVY CHAIN (PTHR11431:SF37) |
| ENSSSCT00000030348 | -0.3796 | 0.0161 | WW domain binding protein 2;WBP2;ortholog | WW DOMAIN-BINDING PROTEIN 2 (PTHR31606:SF4) |
| ENSSSCT00000042486 | -0.3792 | 0.0315 | ZFP36 ring finger protein like 2;ZFP36L2;ortholog | MRNA DECAY ACTIVATOR PROTEIN ZFP36L2 (PTHR12547:SF100) |
| ENSSSCT00000011617 | -0.3690 | 0.0412 | Adducin 3;ADD3;ortholog | GAMMA-ADDUCIN (PTHR10672:SF5) |
| ENSSSCT00000059458 | -0.3632 | 0.0489 | Eukaryotic translation initiation factor 4E binding protein 2;EIF4EBP2;ortholog | EUKARYOTIC TRANSLATION INITIATION FACTOR 4E-BINDING PROTEIN 2 (PTHR12669:SF4) |
| ENSSSCT00000046595 | -0.3580 | 0.0475 | KH and NYN domain containing;KHNYN;ortholog | PROTEIN KHNYN (PTHR12876:SF28) |
| ENSSSCT00000056298 | -0.3552 | 0.0456 | Class E basic helix-loop-helix protein 40;BHLHE40;ortholog | CLASS E BASIC HELIX-LOOP-HELIX PROTEIN 40 (PTHR10985:SF3) |
| ENSSSCT00000003841 | -0.3514 | 0.0095 | EF-hand domain family member D2;EFHD2;ortholog | EF-HAND DOMAIN-CONTAINING PROTEIN D2 (PTHR13025:SF2) |
| ENSSSCT00000010750 | -0.3478 | 0.0280 | RHOF;RHOF;ortholog | RHO-RELATED GTP-BINDING PROTEIN RHOF (PTHR24072:SF99) |
| ENSSSCT00000055030 | -0.3069 | 0.0328 | G protein subunit beta 1;GNB1;ortholog | GUANINE NUCLEOTIDE-BINDING PROTEIN G(I)/G(S)/G(T) SUBUNIT BETA-1 (PTHR19850:SF29) |
| ENSSSCT00000027876 | -0.2945 | 0.0329 | Dynein light chain;DYNLL2;ortholog | DYNEIN LIGHT CHAIN 2, CYTOPLASMIC (PTHR11886:SF35) |
| ENSSSCT00000022886 | -0.2748 | 0.0492 | Phosphatidylinositol transfer protein alpha;PITPNA;ortholog | PHOSPHATIDYLINOSITOL TRANSFER PROTEIN ALPHA ISOFORM (PTHR10658:SF28) |
| ENSSSCT00000007085 | -0.2351 | 0.0465 | Heparin binding growth factor;HDGF;ortholog | HEPATOMA-DERIVED GROWTH FACTOR-RELATED (PTHR12550:SF41) |
| ENSSSCT00000039540 | -0.2199 | 0.0294 | Ras-related C3 botulinum toxin substrate 1;RAC1;ortholog | RAS-RELATED C3 BOTULINUM TOXIN SUBSTRATE 1 (PTHR24072:SF105) |
| ENSSSCT00000046697 | 0.1912 | 0.0322 | Eukaryotic translation initiation factor 3 subunit H;EIF3H;ortholog | EUKARYOTIC TRANSLATION INITIATION FACTOR 3 SUBUNIT H (PTHR10410:SF3) |
| ENSSSCT00000006622 | 0.1979 | 0.0442 | Eukaryotic translation initiation factor 3 subunit E;EIF3E;ortholog | EUKARYOTIC TRANSLATION INITIATION FACTOR 3 SUBUNIT E (PTHR10317:SF0) |
| ENSSSCT00000063468 | 0.2334 | 0.0458 | LSM4 homolog, U6 small nuclear RNA and mRNA degradation associated;LSM4;ortholog | U6 SNRNA-ASSOCIATED SM-LIKE PROTEIN LSM4 (PTHR23338:SF16) |
| ENSSSCT00000018499 | 0.2372 | 0.0453 | 60S ribosomal protein L26-like 1;RPL26L1;ortholog | 60S RIBOSOMAL PROTEIN L26-LIKE 1 (PTHR11143:SF10) |
| ENSSSCT00000051843 | 0.2404 | 0.0477 | 60S ribosomal protein L23;RPL23;ortholog | 60S RIBOSOMAL PROTEIN L23 (PTHR11761:SF8) |
| ENSSSCT00000001170 | 0.2474 | 0.0289 | Uncharacterized protein;DEK;ortholog | PROTEIN DEK (PTHR13468:SF1) |
| ENSSSCT00000040918 | 0.2541 | 0.0251 | 60S ribosomal protein L5;RPL5;ortholog | 60S RIBOSOMAL PROTEIN L5 (PTHR23410:SF12) |
| ENSSSCT00000011019 | 0.2608 | 0.0150 | Small nuclear ribonucleoprotein Sm D3;SNRPD3;ortholog | SMALL NUCLEAR RIBONUCLEOPROTEIN SM D3 (PTHR23338:SF17) |
| ENSSSCT00000053253 | 0.2619 | 0.0478 | Neural precursor cell expressed, developmentally down-regulated 8;NEDD8;ortholog | NEDD8 (PTHR10666:SF173) |
| ENSSSCT00000017625 | 0.2633 | 0.0145 | Arp2/3 complex 34 kDa subunit;ARPC2;ortholog | ACTIN-RELATED PROTEIN 2/3 COMPLEX SUBUNIT 2 (PTHR12058:SF0) |
| ENSSSCT00000016207 | 0.2853 | 0.0440 | 40S ribosomal protein S3;RPS3;ortholog | 40S RIBOSOMAL PROTEIN S3 (PTHR11760:SF32) |
| ENSSSCT00000003287 | 0.2874 | 0.0454 | Eukaryotic translation initiation factor 3 subunit K;EIF3K;ortholog | EUKARYOTIC TRANSLATION INITIATION FACTOR 3 SUBUNIT K (PTHR13022:SF0) |
| ENSSSCT00000035199 | 0.2901 | 0.0312 | Prefoldin subunit 3;VBP1;ortholog | PREFOLDIN SUBUNIT 3 (PTHR12409:SF0) |
| ENSSSCT00000029514 | 0.2914 | 0.0337 | Superoxide dismutase [Cu-Zn];SOD1;ortholog | SUPEROXIDE DISMUTASE [CU-ZN] (PTHR10003:SF58) |
| ENSSSCT00000001743 | 0.3032 | 0.0408 | Serine and arginine rich splicing factor 3;SRSF3;ortholog | SERINE/ARGININE-RICH SPLICING FACTOR 3 (PTHR23147:SF103) |
| ENSSSCT00000045760 | 0.3089 | 0.0136 | Ribosomal protein S27a;RPS27A;ortholog | UBIQUITIN-40S RIBOSOMAL PROTEIN S27A (PTHR10666:SF258) |
| ENSSSCT00000011737 | 0.3138 | 0.0314 | BUB3, mitotic checkpoint protein;BUB3;ortholog | MITOTIC CHECKPOINT PROTEIN BUB3 (PTHR10971:SF5) |
| ENSSSCT00000046956 | 0.3270 | 0.0074 | Peptidyl-prolyl cis-trans isomerase;PPIB;ortholog | PEPTIDYL-PROLYL CIS-TRANS ISOMERASE B (PTHR11071:SF387) |
| ENSSSCT00000004629 | 0.3297 | 0.0238 | Syntaxin 7;STX7;ortholog | SYNTAXIN-7 (PTHR19957:SF90) |
| ENSSSCT00000002569 | 0.3328 | 0.0030 | Enhancer of rudimentary homolog;ERH;ortholog | ENHANCER OF RUDIMENTARY HOMOLOG (PTHR12373:SF0) |
| ENSSSCT00000049763 | 0.3336 | 0.0146 | 60S acidic ribosomal protein P0;RPLP0;ortholog | 60S ACIDIC RIBOSOMAL PROTEIN P0-RELATED (PTHR45699:SF1) |
| ENSSSCT00000057837 | 0.3357 | 0.0236 | DNA polymerase epsilon 4, accessory subunit;POLE4;ortholog | DNA POLYMERASE EPSILON SUBUNIT 4 (PTHR10252:SF79) |
| ENSSSCT00000012095 | 0.3401 | 0.0486 | Heat shock 70 kDa protein 14;HSPA14;ortholog | HEAT SHOCK 70 KDA PROTEIN 14 (PTHR19375:SF331) |
| ENSSSCT00000047169 | 0.3404 | 0.0312 | Uncharacterized protein;unassigned;ortholog | PROTEIN FAM133B (PTHR31911:SF3) |
| ENSSSCT00000064349 | 0.3453 | 0.0359 | RNA polymerase II subunit D;POLR2D;ortholog | DNA-DIRECTED RNA POLYMERASE II SUBUNIT RPB4 (PTHR21297:SF0) |
| ENSSSCT00000004100 | 0.3566 | 0.0221 | Small nuclear ribonucleoprotein D1 polypeptide;SNRPD1;ortholog | SMALL NUCLEAR RIBONUCLEOPROTEIN SM D1 (PTHR23338:SF18) |
| ENSSSCT00000040250 | 0.3574 | 0.0321 | Nucleolar and spindle associated protein 1;NUSAP1;ortholog | NUCLEOLAR AND SPINDLE-ASSOCIATED PROTEIN 1 (PTHR15874:SF1) |
| ENSSSCT00000000089 | 0.3575 | 0.0170 | Ribosomal protein S19 binding protein 1;RPS19BP1;ortholog | ACTIVE REGULATOR OF SIRT1 (PTHR31454:SF2) |
| ENSSSCT00000057207 | 0.3581 | 0.0317 | Eukaryotic translation initiation factor 1A, X-chromosomal;EIF1AX;ortholog | EUKARYOTIC TRANSLATION INITIATION FACTOR 1A, X-CHROMOSOMAL (PTHR21668:SF4) |
| ENSSSCT00000028348 | 0.3593 | 0.0467 | Cyclin-dependent kinase 4;CDK4;ortholog | CYCLIN-DEPENDENT KINASE 4 (PTHR24056:SF129) |
| ENSSSCT00000031620 | 0.3616 | 0.0090 | SRA stem-loop-interacting RNA-binding protein, mitochondrial;SLIRP;ortholog | SRA STEM-LOOP-INTERACTING RNA-BINDING PROTEIN, MITOCHONDRIAL (PTHR15241:SF131) |
| ENSSSCT00000017560 | 0.3721 | 0.0072 | Elongation factor 1-beta;EEF1B2;ortholog | ELONGATION FACTOR 1-BETA (PTHR11595:SF21) |
| ENSSSCT00000036365 | 0.3778 | 0.0430 | Ubiquitously expressed prefoldin like chaperone;UXT;ortholog | PROTEIN UXT (PTHR13345:SF4) |
| ENSSSCT00000022560 | 0.3783 | 0.0160 | RNA polymerase II subunit G;POLR2G;ortholog | DNA-DIRECTED RNA POLYMERASE II SUBUNIT RPB7 (PTHR12709:SF4) |
| ENSSSCT00000000979 | 0.3783 | 0.0195 | Small nuclear ribonucleoprotein F;SNRPF;ortholog | SMALL NUCLEAR RIBONUCLEOPROTEIN F (PTHR11021:SF5) |
| ENSSSCT00000050385 | 0.3844 | 0.0004 | RU2B;SNRPB2;ortholog | U2 SMALL NUCLEAR RIBONUCLEOPROTEIN B'' (PTHR10501:SF21) |
| ENSSSCT00000045474 | 0.3956 | 0.0020 | Ribosomal protein;RPL10A;ortholog | 60S RIBOSOMAL PROTEIN L10A (PTHR23105:SF107) |
| ENSSSCT00000004747 | 0.4029 | 0.0024 | Sorting nexin 14;SNX14;ortholog | SORTING NEXIN-14 (PTHR22775:SF3) |
| ENSSSCT00000024602 | 0.4030 | 0.0151 | 40S ribosomal protein S16;RPS16;ortholog | 40S RIBOSOMAL PROTEIN S16 (PTHR21569:SF4) |
| ENSSSCT00000019290 | 0.4047 | 0.0398 | C-C motif chemokine 2;CCL2;ortholog | C-C MOTIF CHEMOKINE 2 (PTHR12015:SF98) |
| ENSSSCT00000016695 | 0.4050 | 0.0360 | Tissue factor pathway inhibitor;TFPI2;ortholog | TISSUE FACTOR PATHWAY INHIBITOR 2 (PTHR10083:SF336) |
| ENSSSCT00000054538 | 0.4148 | 0.0178 | 40S ribosomal protein S9;RPS9;ortholog | 40S RIBOSOMAL PROTEIN S9 (PTHR11831:SF5) |
| ENSSSCT00000039191 | 0.4197 | 0.0385 | Uncharacterized protein;HMGN1;ortholog | NON-HISTONE CHROMOSOMAL PROTEIN HMG-14 (PTHR23087:SF12) |
| ENSSSCT00000055711 | 0.4251 | 0.0452 | Deoxyribonuclease;DNASE1L3;ortholog | DEOXYRIBONUCLEASE GAMMA (PTHR11371:SF32) |
| ENSSSCT00000017369 | 0.4307 | 0.0267 | Histone acetyltransferase type B catalytic subunit;HAT1;ortholog | HISTONE ACETYLTRANSFERASE TYPE B CATALYTIC SUBUNIT (PTHR12046:SF0) |
| ENSSSCT00000060340 | 0.4468 | 0.0017 | Acyl-CoA-binding protein;DBI;ortholog | ACYL-COA-BINDING PROTEIN (PTHR23310:SF54) |
| ENSSSCT00000000203 | 0.4501 | 0.0195 | Tubulin alpha-1B chain;TUBA1B;ortholog | TUBULIN ALPHA-1B CHAIN (PTHR11588:SF352) |
| ENSSSCT00000042922 | 0.4612 | 0.0434 | Transcription elongation factor A protein 1;TCEA1;ortholog | TRANSCRIPTION ELONGATION FACTOR A PROTEIN 1 (PTHR11477:SF1) |
| ENSSSCT00000031595 | 0.4636 | 0.0017 | Uncharacterized protein;SNRPG;ortholog | SMALL NUCLEAR RIBONUCLEOPROTEIN G (PTHR10553:SF2) |
| ENSSSCT00000040747 | 0.4684 | 0.0382 | Guanine nucleotide-binding protein subunit gamma;GNG2;ortholog | GUANINE NUCLEOTIDE-BINDING PROTEIN G(I)/G(S)/G(O) SUBUNIT GAMMA-2 (PTHR13809:SF25) |
| ENSSSCT00000010764 | 0.4732 | 0.0009 | Actin-related protein 2/3 complex subunit 3;ARPC3;ortholog | ACTIN-RELATED PROTEIN 2/3 COMPLEX SUBUNIT 3 (PTHR12391:SF1) |
| ENSSSCT00000030366 | 0.4835 | 0.0485 | Origin recognition complex subunit 6;ORC6;ortholog | ORIGIN RECOGNITION COMPLEX SUBUNIT 6 (PTHR13394:SF0) |
| ENSSSCT00000006513 | 0.4892 | 0.0133 | Chromatin accessibility complex 1;CHRAC1;ortholog | CHROMATIN ACCESSIBILITY COMPLEX PROTEIN 1 (PTHR10252:SF54) |
| ENSSSCT00000010051 | 0.4946 | 0.0383 | Histone H2A;H2A.Z;ortholog | HISTONE H2A.Z (PTHR23430:SF47) |
| ENSSSCT00000034332 | 0.5062 | 0.0066 | 39S ribosomal protein L13, mitochondrial;MRPL13;ortholog | 39S RIBOSOMAL PROTEIN L13, MITOCHONDRIAL (PTHR11545:SF17) |
| ENSSSCT00000007515 | 0.5151 | 0.0354 | Vascular cell adhesion protein 1 precursor;VCAM1;ortholog | VASCULAR CELL ADHESION PROTEIN 1 (PTHR13771:SF14) |
| ENSSSCT00000027484 | 0.5218 | 0.0386 | Anaphase promoting complex subunit 4;ANAPC4;ortholog | ANAPHASE-PROMOTING COMPLEX SUBUNIT 4 (PTHR13260:SF0) |
| ENSSSCT00000027813 | 0.5470 | 0.0011 | Uncharacterized protein;RPA3;ortholog | REPLICATION PROTEIN A 14 KDA SUBUNIT (PTHR15114:SF1) |
| ENSSSCT00000003776 | 0.5718 | 0.0248 | Centromere protein S;CENPS;ortholog | CENTROMERE PROTEIN S (PTHR22980:SF4) |
| ENSSSCT00000047604 | 0.5723 | 0.0437 | Protection of telomeres protein 1;POT1;ortholog | PROTECTION OF TELOMERES PROTEIN 1 (PTHR14513:SF0) |
| ENSSSCT00000010246 | 0.5724 | 0.0467 | Replication factor C subunit 3;RFC3;ortholog | REPLICATION FACTOR C SUBUNIT 3 (PTHR11669:SF1) |
| ENSSSCT00000009866 | 0.5870 | 0.0242 | Meiotic nuclear division protein 1 homolog;MND1;ortholog | MEIOTIC NUCLEAR DIVISION PROTEIN 1 HOMOLOG (PTHR31398:SF0) |
| ENSSSCT00000005031 | 0.5920 | 0.0216 | PCNA-associated factor;PCLAF;ortholog | PCNA-ASSOCIATED FACTOR (PTHR15679:SF8) |
| ENSSSCT00000043865 | 0.6002 | 0.0045 | Non-histone chromosomal protein HMG-17;HMGN2;ortholog | NON-HISTONE CHROMOSOMAL PROTEIN HMG-17 (PTHR23087:SF13) |
| ENSSSCT00000006941 | 0.6098 | 0.0204 | NUF2, NDC80 kinetochore complex component;NUF2;ortholog | KINETOCHORE PROTEIN NUF2 (PTHR21650:SF2) |
| ENSSSCT00000006754 | 0.6654 | 0.0127 | Stathmin;STMN2;ortholog | STATHMIN-2 (PTHR10104:SF18) |
| ENSSSCT00000012916 | 0.6858 | 0.0022 | Replication factor C subunit 4;RFC4;ortholog | REPLICATION FACTOR C SUBUNIT 4 (PTHR11669:SF20) |
| ENSSSCT00000064385 | 0.7337 | 0.0228 | C-X-C chemokine receptor type 4;CXCR4;ortholog | C-X-C CHEMOKINE RECEPTOR TYPE 4 (PTHR10489:SF594) |
| ENSSSCT00000046422 | 0.8902 | 0.0224 | S1 RNA binding domain 1;SRBD1;ortholog | S1 RNA-BINDING DOMAIN-CONTAINING PROTEIN 1 (PTHR10724:SF1) |
| ENSSSCT00000048761 | 0.9609 | 0.0307 | Chitinase-3-like protein 1;CHI3L1;ortholog | CHITINASE-3-LIKE PROTEIN 1 (PTHR11177:SF202) |
| ENSSSCT00000047882 | 1.0050 | 0.0208 | Clusterin;CLU;ortholog | CLUSTERIN (PTHR10970:SF1) |
| ENSSSCT00000011038 | 1.0365 | 0.0264 | Uncharacterized protein;LOC100523213;ortholog | IG LAMBDA-2 CHAIN C REGION-RELATED (PTHR23266:SF237) |
| ENSSSCT00000017304 | 1.1684 | 0.0289 | Proteasome 26S subunit, non-ATPase 14;PSMD14;ortholog | 26S PROTEASOME NON-ATPASE REGULATORY SUBUNIT 14 (PTHR10410:SF5) |
| ENSSSCT00000007022 | 1.2062 | 0.0166 | Pentaxin;LOC100517891;ortholog | MUCOSAL PENTRAXIN-RELATED (PTHR45869:SF6) |
| ENSSSCT00000016571 | 1.2678 | 0.0237 | Hepatic and glial cell adhesion molecule;HEPACAM;ortholog | HEPATOCYTE CELL ADHESION MOLECULE (PTHR12080:SF59) |
| ENSSSCT00000016304 | 1.4524 | 0.0411 | Centrosomal protein 295;CEP295;ortholog | CENTROSOMAL PROTEIN OF 295 KDA (PTHR21553:SF25) |
| ENSSSCT00000016464 | 2.3630 | 0.0478 | C-X-C motif chemokine receptor 5;CXCR5;ortholog | C-X-C CHEMOKINE RECEPTOR TYPE 5 (PTHR10489:SF618) |
| ENSSSCT00000045699 | 2.6408 | 0.0168 | Myocyte-specific enhancer factor 2C;MEF2C;ortholog | MYOCYTE-SPECIFIC ENHANCER FACTOR 2C (PTHR11945:SF25) |
|  |  |  |  |  |
| **Zn100 vs Zn50** | | | | |
| ENSSSCT00000030537 | -5.0088 | 0.0024 | Histone H4;unassigned;ortholog | HISTONE H4 (PTHR10484:SF163) |
| ENSSSCT00000015136 | -2.1139 | 0.0248 | Epidermal growth factor receptor pathway substrate 15 like 1;EPS15L1;ortholog | EPIDERMAL GROWTH FACTOR RECEPTOR SUBSTRATE 15-LIKE 1 (PTHR11216:SF69) |
| ENSSSCT00000057614 | -2.0293 | 0.0269 | SURP and G-patch domain containing 2;SUGP2;ortholog | SURP AND G-PATCH DOMAIN-CONTAINING PROTEIN 2 (PTHR23340:SF2) |
| ENSSSCT00000062411 | -2.0249 | 0.0211 | Transposase;unassigned;ortholog | LINE-1 RETROTRANSPOSABLE ELEMENT ORF1 PROTEIN (PTHR11505:SF195) |
| ENSSSCT00000029033 | -1.5774 | 0.0216 | Synaptotagmin 12;SYT12;ortholog | SYNAPTOTAGMIN-12 (PTHR10024:SF252) |
| ENSSSCT00000061852 | -1.3456 | 0.0409 | Apolipoprotein A-IV;APOA4;ortholog | APOLIPOPROTEIN A-IV (PTHR18976:SF1) |
| ENSSSCT00000002555 | -0.9370 | 0.0176 | Arginase;ARG2;ortholog | ARGINASE-2, MITOCHONDRIAL (PTHR43782:SF4) |
| ENSSSCT00000002650 | -0.8170 | 0.0107 | Proto-oncogene c-Fos;FOS;ortholog | PROTO-ONCOGENE C-FOS (PTHR23351:SF4) |
| ENSSSCT00000031638 | -0.7751 | 0.0229 | Uncharacterized protein;KDM4A;ortholog | LYSINE-SPECIFIC DEMETHYLASE 4A (PTHR10694:SF32) |
| ENSSSCT00000045848 | -0.7564 | 0.0408 | Krueppel-like factor 3;KLF3;ortholog | KRUEPPEL-LIKE FACTOR 3 (PTHR23235:SF48) |
| ENSSSCT00000028073 | -0.6396 | 0.0254 | Oxysterol-binding protein;OSBPL9;ortholog | OXYSTEROL-BINDING PROTEIN-RELATED PROTEIN 9 (PTHR10972:SF102) |
| ENSSSCT00000053892 | -0.5714 | 0.0000 | ADP-ribosylation factor 1;ARF1;ortholog | ADP-RIBOSYLATION FACTOR 1 (PTHR11711:SF350) |
| ENSSSCT00000018974 | -0.4587 | 0.0218 | Junction plakoglobin;JUP;ortholog | JUNCTION PLAKOGLOBIN (PTHR45976:SF3) |
| ENSSSCT00000015156 | -0.4521 | 0.0186 | Nuclear receptor subfamily 2 group F member 6;NR2F6;ortholog | NUCLEAR RECEPTOR SUBFAMILY 2 GROUP F MEMBER 6 (PTHR24083:SF44) |
| ENSSSCT00000026750 | -0.3119 | 0.0082 | Tristetraprolin;ZFP36;ortholog | MRNA DECAY ACTIVATOR PROTEIN ZFP36 (PTHR12547:SF122) |
| ENSSSCT00000005151 | -0.2946 | 0.0183 | Sulfide:quinone oxidoreductase, mitochondrial;SQOR;ortholog | SULFIDE:QUINONE OXIDOREDUCTASE, MITOCHONDRIAL (PTHR10632:SF2) |
| ENSSSCT00000027876 | -0.2396 | 0.0490 | Dynein light chain;DYNLL2;ortholog | DYNEIN LIGHT CHAIN 2, CYTOPLASMIC (PTHR11886:SF35) |
| ENSSSCT00000004747 | 0.3084 | 0.0336 | Sorting nexin 14;SNX14;ortholog | SORTING NEXIN-14 (PTHR22775:SF3) |
| ENSSSCT00000016593 | 0.3085 | 0.0228 | SRP receptor alpha subunit;SRPRA;ortholog | SIGNAL RECOGNITION PARTICLE RECEPTOR SUBUNIT ALPHA (PTHR43134:SF1) |
| ENSSSCT00000017503 | 0.6669 | 0.0154 | Splicing factor 3b subunit 1;SF3B1;ortholog | SPLICING FACTOR 3B SUBUNIT 1 (PTHR12097:SF0) |
| ENSSSCT00000048062 | 0.6858 | 0.0457 | Heterogeneous nuclear ribonucleoprotein F;HNRNPF;ortholog | HETEROGENEOUS NUCLEAR RIBONUCLEOPROTEIN F (PTHR13976:SF32) |
| ENSSSCT00000045355 | 0.7388 | 0.0369 | Nuclear respiratory factor 1;NRF1;ortholog | NUCLEAR RESPIRATORY FACTOR 1 (PTHR20338:SF8) |
| ENSSSCT00000009388 | 0.7453 | 0.0251 | Centromere protein O;CENPO;ortholog | CENTROMERE PROTEIN O (PTHR14582:SF1) |
| ENSSSCT00000030552 | 0.8145 | 0.0480 | Chemokine C-C motif receptor-like 2;CCRL2;ortholog | C-C CHEMOKINE RECEPTOR-LIKE 2 (PTHR10489:SF655) |
| ENSSSCT00000056515 | 0.8152 | 0.0489 | Uncharacterized protein;LOC100622780;ortholog | SUBFAMILY NOT NAMED (PTHR12019:SF23) |
| ENSSSCT00000057503 | 1.0832 | 0.0103 | Cold-inducible RNA-binding protein;CIRBP;ortholog | COLD-INDUCIBLE RNA-BINDING PROTEIN (PTHR15241:SF45) |
| ENSSSCT00000012375 | 1.1578 | 0.0386 | Zinc finger protein 197;ZNF197;ortholog | ZINC FINGER PROTEIN 197 (PTHR24376:SF139) |
| ENSSSCT00000017155 | 1.2192 | 0.0219 | Microcephalin 1;MCPH1;ortholog | MICROCEPHALIN (PTHR14625:SF3) |
| ENSSSCT00000051344 | 1.3610 | 0.0026 | Tetratricopeptide repeat domain 9C;TTC9C;ortholog | TETRATRICOPEPTIDE REPEAT PROTEIN 9C (PTHR43811:SF1) |
| ENSSSCT00000056812 | 1.5088 | 0.0403 | Regulation of nuclear pre-mRNA domain containing 1A;RPRD1A;ortholog | REGULATION OF NUCLEAR PRE-MRNA DOMAIN-CONTAINING PROTEIN 1A (PTHR12460:SF2) |
| ENSSSCT00000016872 | 1.6435 | 0.0317 | Tumor necrosis factor ligand superfamily member 4;TNFRSF4;ortholog | TUMOR NECROSIS FACTOR LIGAND SUPERFAMILY MEMBER 4 (PTHR17534:SF4) |
| ENSSSCT00000049484 | 1.9607 | 0.0150 | Protein S100-G;S100G;ortholog | PROTEIN S100-G (PTHR11639:SF73) |
| ENSSSCT00000042792 | 2.1415 | 0.0386 | Phosphatidylinositol transfer protein membrane associated 2;PITPNM2;ortholog | MEMBRANE-ASSOCIATED PHOSPHATIDYLINOSITOL TRANSFER PROTEIN 2 (PTHR10658:SF41) |
| ENSSSCT00000032296 | 2.1700 | 0.0384 | Espin;ESPN;ortholog | ESPIN (PTHR24153:SF14) |
| ENSSSCT00000016464 | 2.2650 | 0.0278 | C-X-C motif chemokine receptor 5;CXCR5;ortholog | C-X-C CHEMOKINE RECEPTOR TYPE 5 (PTHR10489:SF618) |
| ENSSSCT00000064122 | 2.4362 | 0.0395 | Microtubule-associated protein RP/EB family member 1;MAPRE1;ortholog | MICROTUBULE-ASSOCIATED PROTEIN RP/EB FAMILY MEMBER 1 (PTHR10623:SF20) |
| ENSSSCT00000058397 | 2.6129 | 0.0008 | Metallothionein;LOC100739663;ortholog | METALLOTHIONEIN-1A (PTHR23299:SF22) |
| ENSSSCT00000040161 | 2.8333 | 0.0088 | Regulatory factor X7;RFX7;ortholog | DNA-BINDING PROTEIN RFX7 (PTHR12619:SF2) |
| ENSSSCT00000029094 | 3.6111 | 0.0003 | Metallothionein-1A;MT1A;ortholog | METALLOTHIONEIN-1A (PTHR23299:SF22) |
|  |  |  |  |  |

**Supplementary Table 6. Genes related to catalytic activity**

| **ID** | **Log2 Fold Change** | **p-value** | **Gene Name** | **Panther Family** |
| --- | --- | --- | --- | --- |
| **ZnR vs Zn100** | | | | |
| ENSSSCT00000045796 | -3.11070 | 0.02575 | Methylsterol monooxygenase 1;SC4MOL;ortholog | METHYLSTEROL MONOOXYGENASE 1 (PTHR11863:SF22) |
| ENSSSCT00000065632 | -2.69089 | 0.04104 | Pellino E3 ubiquitin protein ligase 1;PELI1;ortholog | E3 UBIQUITIN-PROTEIN LIGASE PELLINO HOMOLOG 1 (PTHR12098:SF4) |
| ENSSSCT00000027940 | -2.18338 | 0.03148 | Phosphodiesterase;PDE3A;ortholog | PHOSPHODIESTERASE (PTHR11347:SF104) |
| ENSSSCT00000018038 | -1.98570 | 0.02290 | Adenosylhomocysteinase;AHCYL2;ortholog | ADENOSYLHOMOCYSTEINASE 3 (PTHR23420:SF2) |
| ENSSSCT00000018711 | -1.71865 | 0.00856 | Ring finger protein 157;RNF157;ortholog | E3 UBIQUITIN LIGASE RNF157 (PTHR22996:SF1) |
| ENSSSCT00000000055 | -1.57093 | 0.00229 | Alpha-N-acetylgalactosaminidase precursor;NAGA;ortholog | ALPHA-N-ACETYLGALACTOSAMINIDASE (PTHR11452:SF25) |
| ENSSSCT00000018904 | -1.50794 | 0.02344 | Glucose-6-phosphatase;G6PC3;ortholog | GLUCOSE-6-PHOSPHATASE 3 (PTHR12591:SF2) |
| ENSSSCT00000050670 | -1.34421 | 0.01063 | Coenzyme Q8A;COQ8A;ortholog | ATYPICAL KINASE COQ8A, MITOCHONDRIAL (PTHR43851:SF1) |
| ENSSSCT00000062766 | -1.09852 | 0.00962 | Phospholipid-transporting ATPase;ATP11B;ortholog | PHOSPHOLIPID-TRANSPORTING ATPASE IF-RELATED (PTHR24092:SF57) |
| ENSSSCT00000062485 | -1.07307 | 0.01701 | ADP-ribosylation factor GTPase-activating protein 2;ARFGAP2;ortholog | ADP-RIBOSYLATION FACTOR GTPASE-ACTIVATING PROTEIN 2 (PTHR45686:SF8) |
| ENSSSCT00000045618 | -0.99941 | 0.02843 | Deltex E3 ubiquitin ligase 1;DTX1;ortholog | E3 UBIQUITIN-PROTEIN LIGASE DTX1 (PTHR12622:SF7) |
| ENSSSCT00000008841 | -0.93274 | 0.02381 | Isoleucyl-tRNA synthetase;IARS;ortholog | ISOLEUCINE--TRNA LIGASE, CYTOPLASMIC (PTHR42780:SF1) |
| ENSSSCT00000027578 | -0.87641 | 0.04252 | Kinesin-like protein;KIFC2;ortholog | KINESIN-LIKE PROTEIN KIFC2 (PTHR24115:SF479) |
| ENSSSCT00000024419 | -0.85391 | 0.04148 | Dual specificity tyrosine phosphorylation regulated kinase 1B;DYRK1B;ortholog | DUAL SPECIFICITY TYROSINE-PHOSPHORYLATION-REGULATED KINASE 1B (PTHR24058:SF111) |
| ENSSSCT00000026311 | -0.82486 | 0.02325 | Uncharacterized protein;unassigned;ortholog | HEAT SHOCK 70 KDA PROTEIN 1A-RELATED (PTHR19375:SF223) |
| ENSSSCT00000044362 | -0.79819 | 0.03492 | Ring finger protein 145;RNF145;ortholog | RING FINGER PROTEIN 145 (PTHR22763:SF167) |
| ENSSSCT00000018503 | -0.79057 | 0.02895 | Neuralized E3 ubiquitin protein ligase 1B;NEURL1B;ortholog | E3 UBIQUITIN-PROTEIN LIGASE NEURL1B (PTHR12429:SF10) |
| ENSSSCT00000016654 | -0.72425 | 0.01203 | Phosphatidylinositol-4-phosphate 3-kinase catalytic subunit type 2 beta;PIK3C2B;ortholog | PHOSPHATIDYLINOSITOL 4-PHOSPHATE 3-KINASE C2 DOMAIN-CONTAINING SUBUNIT BETA (PTHR10048:SF30) |
| ENSSSCT00000013417 | -0.71292 | 0.02073 | Ubiquitin like modifier activating enzyme 1;UBA1;ortholog | SUMO-ACTIVATING ENZYME SUBUNIT 1-RELATED (PTHR10953:SF211) |
| ENSSSCT00000009844 | -0.66859 | 0.01427 | Cyclin I;CCNI;ortholog | CYCLIN-I (PTHR10177:SF187) |
| ENSSSCT00000028916 | -0.65371 | 0.04346 | Aldehyde dehydrogenase 1 family member A3;ALDH1A3;ortholog | ALDEHYDE DEHYDROGENASE FAMILY 1 MEMBER A3 (PTHR11699:SF209) |
| ENSSSCT00000041445 | -0.63078 | 0.02306 | Leukocyte receptor cluster member 8;LENG8;ortholog | LEUKOCYTE RECEPTOR CLUSTER MEMBER 8 (PTHR12436:SF4) |
| ENSSSCT00000027501 | -0.60987 | 0.02594 | Glucose-6-phosphate 1-dehydrogenase;G6PD;ortholog | GLUCOSE-6-PHOSPHATE 1-DEHYDROGENASE (PTHR23429:SF0) |
| ENSSSCT00000052686 | -0.50658 | 0.04580 | RAB8B, member RAS oncogene family;RAB8B;ortholog | RAS-RELATED PROTEIN RAB-8B (PTHR24073:SF22) |
| ENSSSCT00000014210 | -0.38160 | 0.03814 | Calpain-1 catalytic subunit;CAPN1;ortholog | CALPAIN-1 CATALYTIC SUBUNIT (PTHR10183:SF284) |
| ENSSSCT00000017493 | -0.22174 | 0.02701 | Serine/threonine kinase 17b;STK17B;ortholog | SERINE/THREONINE-PROTEIN KINASE 17B (PTHR24342:SF5) |
| ENSSSCT00000000992 | 0.23785 | 0.02196 | Uncharacterized protein;unassigned;ortholog | UBIQUITIN-CONJUGATING ENZYME E2 N (PTHR24068:SF152) |
| ENSSSCT00000002293 | 0.24159 | 0.03820 | Dolichyl-diphosphooligosaccharide--protein glycosyltransferase subunit DAD1;DAD1;ortholog | DOLICHYL-DIPHOSPHOOLIGOSACCHARIDE--PROTEIN GLYCOSYLTRANSFERASE SUBUNIT DAD1 (PTHR10705:SF0) |
| ENSSSCT00000029514 | 0.24920 | 0.04211 | Superoxide dismutase [Cu-Zn];SOD1;ortholog | SUPEROXIDE DISMUTASE [CU-ZN] (PTHR10003:SF58) |
| ENSSSCT00000028465 | 0.25588 | 0.00692 | Cytochrome c oxidase subunit NDUFA4;NDUFA4;ortholog | CYTOCHROME C OXIDASE SUBUNIT NDUFA4 (PTHR14256:SF4) |
| ENSSSCT00000006972 | 0.27550 | 0.01382 | Ubiquitin-fold modifier-conjugating enzyme 1;UFC1;ortholog | UBIQUITIN-FOLD MODIFIER-CONJUGATING ENZYME 1 (PTHR12921:SF0) |
| ENSSSCT00000006146 | 0.28310 | 0.00651 | Proteasome subunit beta type-7;PSMB7;ortholog | PROTEASOME SUBUNIT BETA TYPE-7 (PTHR11599:SF42) |
| ENSSSCT00000022560 | 0.29063 | 0.03328 | RNA polymerase II subunit G;POLR2G;ortholog | DNA-DIRECTED RNA POLYMERASE II SUBUNIT RPB7 (PTHR12709:SF4) |
| ENSSSCT00000019150 | 0.29584 | 0.02431 | Nucleoside diphosphate kinase B;NME2;ortholog | NUCLEOSIDE DIPHOSPHATE KINASE B (PTHR11349:SF57) |
| ENSSSCT00000010685 | 0.30008 | 0.04126 | GTP-binding nuclear protein Ran;RAN;ortholog | GTP-BINDING NUCLEAR PROTEIN RAN (PTHR24071:SF0) |
| ENSSSCT00000016800 | 0.30544 | 0.02103 | Peptidase, mitochondrial processing beta subunit;PMPCB;ortholog | MITOCHONDRIAL-PROCESSING PEPTIDASE SUBUNIT BETA (PTHR11851:SF103) |
| ENSSSCT00000005151 | 0.31932 | 0.03139 | Sulfide:quinone oxidoreductase, mitochondrial;SQOR;ortholog | SULFIDE:QUINONE OXIDOREDUCTASE, MITOCHONDRIAL (PTHR10632:SF2) |
| ENSSSCT00000052062 | 0.32701 | 0.01928 | Inosine triphosphate pyrophosphatase;ITPA;ortholog | INOSINE TRIPHOSPHATE PYROPHOSPHATASE (PTHR11067:SF9) |
| ENSSSCT00000057144 | 0.33497 | 0.02850 | Carboxypeptidase;SCPEP1;ortholog | RETINOID-INDUCIBLE SERINE CARBOXYPEPTIDASE (PTHR11802:SF3) |
| ENSSSCT00000053097 | 0.34117 | 0.00534 | Uncharacterized protein;UBE2E3;ortholog | UBIQUITIN-CONJUGATING ENZYME E2 E3 (PTHR24068:SF63) |
| ENSSSCT00000025596 | 0.41489 | 0.03163 | Cyclin-dependent kinases regulatory subunit;CKS2;ortholog | CYCLIN-DEPENDENT KINASES REGULATORY SUBUNIT 2 (PTHR23415:SF35) |
| ENSSSCT00000039736 | 0.42584 | 0.02480 | Uncharacterized protein;NUDT15;ortholog | NUCLEOTIDE TRIPHOSPHATE DIPHOSPHATASE NUDT15 (PTHR16099:SF5) |
| ENSSSCT00000013313 | 0.44192 | 0.00221 | Peroxiredoxin 4;PRDX4;ortholog | PEROXIREDOXIN-4 (PTHR10681:SF128) |
| ENSSSCT00000061063 | 0.44607 | 0.00950 | Platelet-activating factor acetylhydrolase;PAF-AH;ortholog | PLATELET-ACTIVATING FACTOR ACETYLHYDROLASE (PTHR10272:SF12) |
| ENSSSCT00000012916 | 0.49086 | 0.04027 | Replication factor C subunit 4;RFC4;ortholog | REPLICATION FACTOR C SUBUNIT 4 (PTHR11669:SF20) |
| ENSSSCT00000045632 | 0.51282 | 0.00522 | Citrate synthase;CS;ortholog | CITRATE SYNTHASE, MITOCHONDRIAL (PTHR11739:SF8) |
| ENSSSCT00000006347 | 0.73527 | 0.01028 | Haloacid dehalogenase like hydrolase domain containing 2;HDHD2;ortholog | HALOACID DEHALOGENASE-LIKE HYDROLASE DOMAIN-CONTAINING PROTEIN 2 (PTHR19288:SF43) |
| ENSSSCT00000050456 | 1.14316 | 0.03970 | Peptidylprolyl isomerase;FKBP5;ortholog | PEPTIDYL-PROLYL CIS-TRANS ISOMERASE FKBP5 (PTHR10516:SF26) |
| ENSSSCT00000043331 | 1.42015 | 0.00175 | TATA-box binding protein associated factor 9;TAF9;ortholog | TRANSCRIPTION INITIATION FACTOR TFIID SUBUNIT 9 (PTHR12595:SF8) |
| ENSSSCT00000016571 | 1.46304 | 0.01304 | Hepatic and glial cell adhesion molecule;HEPACAM;ortholog | HEPATOCYTE CELL ADHESION MOLECULE (PTHR12080:SF59) |
| ENSSSCT00000017304 | 1.83922 | 0.00651 | Proteasome 26S subunit, non-ATPase 14;PSMD14;ortholog | 26S PROTEASOME NON-ATPASE REGULATORY SUBUNIT 14 (PTHR10410:SF5) |
| ENSSSCT00000044466 | 2.02843 | 0.04482 | Sulfurtransferase;TST;ortholog | THIOSULFATE SULFURTRANSFERASE (PTHR11364:SF6) |
| ENSSSCT00000008482 | 2.12321 | 0.00028 | Protein-tyrosine sulfotransferase;TPST1;ortholog | PROTEIN-TYROSINE SULFOTRANSFERASE 1 (PTHR12788:SF4) |
| ENSSSCT00000042090 | 3.42977 | 0.02305 | Caspase-3;CASP3;ortholog | CASPASE-3 (PTHR10454:SF198) |
|  |  |  |  |  |
| **ZnR vs Zn50** | | | | |
| ENSSSCT00000047688 | -4.27104 | 0.00053 | Uncharacterized protein;RFFL;ortholog | E3 UBIQUITIN-PROTEIN LIGASE RIFIFYLIN-RELATED (PTHR14879:SF2) |
| ENSSSCT00000046592 | -3.62902 | 0.04807 | RBR-type E3 ubiquitin transferase;RNF144A;ortholog | E3 UBIQUITIN-PROTEIN LIGASE RNF144A (PTHR11685:SF99) |
| ENSSSCT00000023398 | -2.37646 | 0.02181 | 5'-nucleotidase domain containing 3;NT5DC3;ortholog | 5'-NUCLEOTIDASE DOMAIN-CONTAINING PROTEIN 3 (PTHR12103:SF11) |
| ENSSSCT00000050440 | -2.36737 | 0.04103 | Microsomal glutathione S-transferase 3;MGST3;ortholog | MICROSOMAL GLUTATHIONE S-TRANSFERASE 3 (PTHR10250:SF17) |
| ENSSSCT00000000278 | -2.06873 | 0.04444 | Cysteine sulfinic acid decarboxylase;CSAD;ortholog | CYSTEINE SULFINIC ACID DECARBOXYLASE (PTHR45677:SF7) |
| ENSSSCT00000018711 | -1.56030 | 0.02090 | Ring finger protein 157;RNF157;ortholog | E3 UBIQUITIN LIGASE RNF157 (PTHR22996:SF1) |
| ENSSSCT00000036127 | -1.47074 | 0.02155 | Palmitoyltransferase;ZDHHC9;ortholog | PALMITOYLTRANSFERASE ZDHHC9 (PTHR22883:SF71) |
| ENSSSCT00000026311 | -1.45346 | 0.00009 | Uncharacterized protein;unassigned;ortholog | HEAT SHOCK 70 KDA PROTEIN 1A-RELATED (PTHR19375:SF223) |
| ENSSSCT00000022686 | -1.43395 | 0.01188 | Cytochrome P450 2C42;CYP2C42;ortholog | CYTOCHROME P450 2C18 (PTHR24300:SF292) |
| ENSSSCT00000045468 | -1.39350 | 0.00454 | Histo-blood group ABO system transferase;GBGT1;ortholog | GLOBOSIDE ALPHA-1,3-N-ACETYLGALACTOSAMINYLTRANSFERASE 1 (PTHR10462:SF29) |
| ENSSSCT00000049672 | -1.12231 | 0.03347 | Fructose-1,6-bisphosphatase isozyme 2;FBP2;ortholog | FRUCTOSE-1,6-BISPHOSPHATASE ISOZYME 2 (PTHR11556:SF13) |
| ENSSSCT00000016214 | -1.10373 | 0.01754 | 2-acylglycerol O-acyltransferase 2;MOGAT2;ortholog | 2-ACYLGLYCEROL O-ACYLTRANSFERASE 2 (PTHR12317:SF5) |
| ENSSSCT00000065647 | -0.98228 | 0.00634 | Plexin B2;PLXNB2;ortholog | PLEXIN-B2 (PTHR22625:SF9) |
| ENSSSCT00000024050 | -0.98094 | 0.01922 | Creatine kinase B-type;CKB;ortholog | CREATINE KINASE B-TYPE (PTHR11547:SF23) |
| ENSSSCT00000018503 | -0.97844 | 0.02281 | Neuralized E3 ubiquitin protein ligase 1B;NEURL1B;ortholog | E3 UBIQUITIN-PROTEIN LIGASE NEURL1B (PTHR12429:SF10) |
| ENSSSCT00000055212 | -0.95119 | 0.02901 | Interferon-induced GTP-binding protein Mx2;MX2;ortholog | INTERFERON-INDUCED GTP-BINDING PROTEIN MX2 (PTHR11566:SF46) |
| ENSSSCT00000031440 | -0.91215 | 0.04748 | RAS like proto-oncogene B;RALB;ortholog | RAS-RELATED PROTEIN RAL-B (PTHR24070:SF199) |
| ENSSSCT00000014281 | -0.89677 | 0.00158 | Asparaginase like 1;ASRGL1;ortholog | ISOASPARTYL PEPTIDASE/L-ASPARAGINASE (PTHR10188:SF30) |
| ENSSSCT00000013832 | -0.89581 | 0.00043 | Xaa-Pro aminopeptidase 2;XPNPEP2;ortholog | XAA-PRO AMINOPEPTIDASE 2 (PTHR43763:SF4) |
| ENSSSCT00000033088 | -0.84311 | 0.01358 | Membrane palmitoylated protein 1;MPP1;ortholog | 55 KDA ERYTHROCYTE MEMBRANE PROTEIN (PTHR23122:SF37) |
| ENSSSCT00000058447 | -0.82135 | 0.03842 | Uncharacterized protein;CYP4F2;ortholog | PHYLLOQUINONE OMEGA-HYDROXYLASE CYP4F11 (PTHR24291:SF112) |
| ENSSSCT00000055405 | -0.78589 | 0.02908 | Estradiol 17-beta-dehydrogenase 2;HSD17B2;ortholog | ESTRADIOL 17-BETA-DEHYDROGENASE 2 (PTHR43313:SF3) |
| ENSSSCT00000064614 | -0.77515 | 0.03298 | SplA/ryanodine receptor domain and SOCS box containing 4;SPSB4;ortholog | SPRY DOMAIN-CONTAINING SOCS BOX PROTEIN 4 (PTHR12245:SF3) |
| ENSSSCT00000061681 | -0.76314 | 0.00656 | Argininosuccinate synthase 1;ASS1;ortholog | ARGININOSUCCINATE SYNTHASE (PTHR11587:SF2) |
| ENSSSCT00000063535 | -0.74830 | 0.00762 | Citrate synthase;CS;ortholog | CITRATE SYNTHASE, MITOCHONDRIAL (PTHR11739:SF8) |
| ENSSSCT00000054120 | -0.72672 | 0.00449 | LLGL2, scribble cell polarity complex component;LLGL2;ortholog | LETHAL(2) GIANT LARVAE PROTEIN HOMOLOG 2 (PTHR10241:SF20) |
| ENSSSCT00000012494 | -0.70221 | 0.00237 | CISH;CISH_tv2;ortholog | CYTOKINE-INDUCIBLE SH2-CONTAINING PROTEIN (PTHR10155:SF9) |
| ENSSSCT00000009460 | -0.69785 | 0.02987 | Cytidine/uridine monophosphate kinase 2;CMPK2;ortholog | UMP-CMP KINASE 2, MITOCHONDRIAL (PTHR10344:SF3) |
| ENSSSCT00000043113 | -0.69514 | 0.04863 | Protein kinase C;PRKCZ;ortholog | PROTEIN KINASE C ZETA TYPE (PTHR24356:SF170) |
| ENSSSCT00000026262 | -0.69501 | 0.01765 | DEAQ-box RNA dependent ATPase 1;DQX1;ortholog | ATP-DEPENDENT RNA HELICASE DQX1 (PTHR18934:SF108) |
| ENSSSCT00000017903 | -0.68399 | 0.00425 | Amiloride-sensitive amine oxidase [copper-containing];AOC1;ortholog | AMILORIDE-SENSITIVE AMINE OXIDASE [COPPER-CONTAINING] (PTHR10638:SF3) |
| ENSSSCT00000048990 | -0.68387 | 0.01484 | Myosin ID;MYO1D;ortholog | UNCONVENTIONAL MYOSIN-ID (PTHR13140:SF417) |
| ENSSSCT00000035626 | -0.67324 | 0.00873 | Aminopeptidase;ANPEP;ortholog | AMINOPEPTIDASE N (PTHR11533:SF172) |
| ENSSSCT00000033376 | -0.67134 | 0.01066 | Dual specificity protein phosphatase;DUSP1;ortholog | DUAL SPECIFICITY PROTEIN PHOSPHATASE 1 (PTHR10159:SF309) |
| ENSSSCT00000043606 | -0.66883 | 0.00701 | RAB5B, member RAS oncogene family;RAB5B;ortholog | RAS-RELATED PROTEIN RAB-5B (PTHR24073:SF555) |
| ENSSSCT00000014247 | -0.65319 | 0.00973 | Phosphoinositide phospholipase C;PLCB3;ortholog | 1-PHOSPHATIDYLINOSITOL 4,5-BISPHOSPHATE PHOSPHODIESTERASE BETA-3 (PTHR10336:SF11) |
| ENSSSCT00000016228 | -0.65281 | 0.01066 | Calpain 5;CAPN5;ortholog | CALPAIN-5 (PTHR10183:SF400) |
| ENSSSCT00000027155 | -0.63258 | 0.04167 | Guanine nucleotide-binding protein subunit alpha-11;GNA11;ortholog | GUANINE NUCLEOTIDE-BINDING PROTEIN SUBUNIT ALPHA-11 (PTHR10218:SF57) |
| ENSSSCT00000016232 | -0.60513 | 0.01169 | P21 (RAC1) activated kinase 1;PAK1;ortholog | SERINE/THREONINE-PROTEIN KINASE PAK 1 (PTHR24361:SF232) |
| ENSSSCT00000024524 | -0.59541 | 0.01004 | Uncharacterized protein;PPP4R1;ortholog | SERINE/THREONINE-PROTEIN PHOSPHATASE 4 REGULATORY SUBUNIT 1 (PTHR10648:SF8) |
| ENSSSCT00000041445 | -0.56217 | 0.03165 | Leukocyte receptor cluster member 8;LENG8;ortholog | LEUKOCYTE RECEPTOR CLUSTER MEMBER 8 (PTHR12436:SF4) |
| ENSSSCT00000004328 | -0.55159 | 0.03600 | Microtubule associated serine/threonine kinase 2;MAST2;ortholog | MICROTUBULE-ASSOCIATED SERINE/THREONINE-PROTEIN KINASE 2 (PTHR24356:SF136) |
| ENSSSCT00000003977 | -0.54428 | 0.04698 | Sphingomyelin phosphodiesterase acid like 3B;SMPDL3B;ortholog | ACID SPHINGOMYELINASE-LIKE PHOSPHODIESTERASE 3B (PTHR10340:SF25) |
| ENSSSCT00000045040 | -0.54274 | 0.03463 | Poly(A) RNA polymerase D5, non-canonical;PAPD5;ortholog | NON-CANONICAL POLY(A) RNA POLYMERASE PAPD5 (PTHR23092:SF49) |
| ENSSSCT00000050794 | -0.54236 | 0.03023 | Interferon-induced, double-stranded RNA-activated protein kinase;EIF2AK2;ortholog | INTERFERON-INDUCED, DOUBLE-STRANDED RNA-ACTIVATED PROTEIN KINASE (PTHR11042:SF91) |
| ENSSSCT00000019254 | -0.53570 | 0.03907 | Dehydrogenase/reductase SDR family member 11;DHRS11;ortholog | DEHYDROGENASE/REDUCTASE SDR FAMILY MEMBER 11 (PTHR43115:SF4) |
| ENSSSCT00000026198 | -0.53068 | 0.03248 | RB1 inducible coiled-coil 1;RB1CC1;ortholog | RB1-INDUCIBLE COILED-COIL PROTEIN 1 (PTHR13222:SF1) |
| ENSSSCT00000029636 | -0.50558 | 0.04890 | Suppressor of cytokine signaling 1;socs1;ortholog | SUPPRESSOR OF CYTOKINE SIGNALING 1 (PTHR10155:SF4) |
| ENSSSCT00000004219 | -0.50439 | 0.00459 | Phosphoglucomutase-1;PGM1;ortholog | PHOSPHOGLUCOMUTASE-1 (PTHR22573:SF37) |
| ENSSSCT00000016661 | -0.50123 | 0.04620 | Cyclin dependent kinase 18;CDK18;ortholog | CYCLIN-DEPENDENT KINASE 18 (PTHR24056:SF52) |
| ENSSSCT00000005194 | -0.50087 | 0.01339 | Creatine kinase U-type, mitochondrial;CKMT1A;ortholog | CREATINE KINASE U-TYPE, MITOCHONDRIAL (PTHR11547:SF24) |
| ENSSSCT00000060796 | -0.49457 | 0.03518 | Mitogen-activated protein kinase;MAPK3;ortholog | MITOGEN-ACTIVATED PROTEIN KINASE 3 (PTHR24055:SF374) |
| ENSSSCT00000005901 | -0.48976 | 0.02049 | Aldehyde dehydrogenase 1 family member B1;ALDH1B1;ortholog | ALDEHYDE DEHYDROGENASE X, MITOCHONDRIAL (PTHR11699:SF207) |
| ENSSSCT00000053600 | -0.46518 | 0.04157 | Rho GTPase activating protein 17;ARHGAP17;ortholog | RHO GTPASE-ACTIVATING PROTEIN 17 (PTHR14130:SF3) |
| ENSSSCT00000019491 | -0.46235 | 0.04485 | Misshapen like kinase 1;MINK1;ortholog | MISSHAPEN-LIKE KINASE 1 (PTHR24361:SF196) |
| ENSSSCT00000051241 | -0.45995 | 0.04565 | Caspase 7;CASP7;ortholog | CASPASE-7 (PTHR10454:SF31) |
| ENSSSCT00000003721 | -0.44558 | 0.02545 | Uncharacterized protein;CDK11B;ortholog | CYCLIN-DEPENDENT KINASE 11A-RELATED (PTHR24056:SF107) |
| ENSSSCT00000005858 | -0.43964 | 0.00847 | Non-lysosomal glucosylceramidase;GBA2;ortholog | NON-LYSOSOMAL GLUCOSYLCERAMIDASE (PTHR12654:SF0) |
| ENSSSCT00000053991 | -0.41149 | 0.02078 | RAB7A, member RAS oncogene family;RAB7A;ortholog | RAS-RELATED PROTEIN RAB-7A (PTHR24073:SF556) |
| ENSSSCT00000009288 | -0.40171 | 0.02712 | Aldose 1-epimerase;GALM;ortholog | ALDOSE 1-EPIMERASE (PTHR10091:SF0) |
| ENSSSCT00000000070 | -0.38504 | 0.01504 | Aconitate hydratase, mitochondrial;ACO2;ortholog | ACONITATE HYDRATASE, MITOCHONDRIAL (PTHR43160:SF3) |
| ENSSSCT00000014210 | -0.38361 | 0.03423 | Calpain-1 catalytic subunit;CAPN1;ortholog | CALPAIN-1 CATALYTIC SUBUNIT (PTHR10183:SF284) |
| ENSSSCT00000044391 | -0.38175 | 0.02604 | Ferritin;FTH1;ortholog | FERRITIN HEAVY CHAIN (PTHR11431:SF37) |
| ENSSSCT00000001752 | -0.36865 | 0.03851 | Pim-1 proto-oncogene, serine/threonine kinase;PIM1;ortholog | SERINE/THREONINE-PROTEIN KINASE PIM-1 (PTHR22984:SF25) |
| ENSSSCT00000010750 | -0.34775 | 0.02801 | RHOF;RHOF;ortholog | RHO-RELATED GTP-BINDING PROTEIN RHOF (PTHR24072:SF99) |
| ENSSSCT00000055030 | -0.30692 | 0.03280 | G protein subunit beta 1;GNB1;ortholog | GUANINE NUCLEOTIDE-BINDING PROTEIN G(I)/G(S)/G(T) SUBUNIT BETA-1 (PTHR19850:SF29) |
| ENSSSCT00000027876 | -0.29447 | 0.03289 | Dynein light chain;DYNLL2;ortholog | DYNEIN LIGHT CHAIN 2, CYTOPLASMIC (PTHR11886:SF35) |
| ENSSSCT00000039540 | -0.21993 | 0.02937 | Ras-related C3 botulinum toxin substrate 1;RAC1;ortholog | RAS-RELATED C3 BOTULINUM TOXIN SUBSTRATE 1 (PTHR24072:SF105) |
| ENSSSCT00000046697 | 0.19120 | 0.03215 | Eukaryotic translation initiation factor 3 subunit H;EIF3H;ortholog | EUKARYOTIC TRANSLATION INITIATION FACTOR 3 SUBUNIT H (PTHR10410:SF3) |
| ENSSSCT00000065836 | 0.22392 | 0.03835 | Proteasome subunit alpha type;PSMA4;ortholog | PROTEASOME SUBUNIT ALPHA TYPE-4 (PTHR11599:SF13) |
| ENSSSCT00000058189 | 0.24230 | 0.04043 | Proteasome subunit beta type;PSMB1;ortholog | PROTEASOME SUBUNIT BETA TYPE-1 (PTHR11599:SF59) |
| ENSSSCT00000014628 | 0.25181 | 0.02407 | Proteasome subunit alpha type;PSMA1;ortholog | PROTEASOME SUBUNIT ALPHA TYPE-RELATED (PTHR11599:SF12) |
| ENSSSCT00000006146 | 0.27675 | 0.02271 | Proteasome subunit beta type-7;PSMB7;ortholog | PROTEASOME SUBUNIT BETA TYPE-7 (PTHR11599:SF42) |
| ENSSSCT00000016207 | 0.28528 | 0.04403 | 40S ribosomal protein S3;RPS3;ortholog | 40S RIBOSOMAL PROTEIN S3 (PTHR11760:SF32) |
| ENSSSCT00000029514 | 0.29136 | 0.03366 | Superoxide dismutase [Cu-Zn];SOD1;ortholog | SUPEROXIDE DISMUTASE [CU-ZN] (PTHR10003:SF58) |
| ENSSSCT00000019150 | 0.30000 | 0.02188 | Nucleoside diphosphate kinase B;NME2;ortholog | NUCLEOSIDE DIPHOSPHATE KINASE B (PTHR11349:SF57) |
| ENSSSCT00000000992 | 0.30545 | 0.00380 | Uncharacterized protein;unassigned;ortholog | UBIQUITIN-CONJUGATING ENZYME E2 N (PTHR24068:SF152) |
| ENSSSCT00000008429 | 0.30560 | 0.03576 | RNA polymerase II subunit J;POLR2J;ortholog | DNA-DIRECTED RNA POLYMERASE II SUBUNIT RPB11-A-RELATED (PTHR13946:SF32) |
| ENSSSCT00000019495 | 0.33295 | 0.02133 | Proteasome subunit beta type;PSMB6;ortholog | PROTEASOME SUBUNIT BETA TYPE-6 (PTHR11599:SF46) |
| ENSSSCT00000016800 | 0.33616 | 0.00802 | Peptidase, mitochondrial processing beta subunit;PMPCB;ortholog | MITOCHONDRIAL-PROCESSING PEPTIDASE SUBUNIT BETA (PTHR11851:SF103) |
| ENSSSCT00000012095 | 0.34006 | 0.04862 | Heat shock 70 kDa protein 14;HSPA14;ortholog | HEAT SHOCK 70 KDA PROTEIN 14 (PTHR19375:SF331) |
| ENSSSCT00000047169 | 0.34037 | 0.03123 | Uncharacterized protein;unassigned;ortholog | PROTEIN FAM133B (PTHR31911:SF3) |
| ENSSSCT00000013152 | 0.34136 | 0.01068 | Mitochondrial ribosomal protein L39;MRPL39;ortholog | 39S RIBOSOMAL PROTEIN L39, MITOCHONDRIAL (PTHR42753:SF9) |
| ENSSSCT00000064349 | 0.34531 | 0.03591 | RNA polymerase II subunit D;POLR2D;ortholog | DNA-DIRECTED RNA POLYMERASE II SUBUNIT RPB4 (PTHR21297:SF0) |
| ENSSSCT00000012875 | 0.34668 | 0.03035 | Uncharacterized protein;NDUFB5;ortholog | NADH DEHYDROGENASE [UBIQUINONE] 1 BETA SUBCOMPLEX SUBUNIT 5, MITOCHONDRIAL (PTHR13178:SF0) |
| ENSSSCT00000028348 | 0.35930 | 0.04669 | Cyclin-dependent kinase 4;CDK4;ortholog | CYCLIN-DEPENDENT KINASE 4 (PTHR24056:SF129) |
| ENSSSCT00000058122 | 0.36811 | 0.00621 | Complement C1s subcomponent precursor;C1S;ortholog | COMPLEMENT C1S SUBCOMPONENT (PTHR24255:SF18) |
| ENSSSCT00000010685 | 0.36959 | 0.01538 | GTP-binding nuclear protein Ran;RAN;ortholog | GTP-BINDING NUCLEAR PROTEIN RAN (PTHR24071:SF0) |
| ENSSSCT00000022560 | 0.37825 | 0.01602 | RNA polymerase II subunit G;POLR2G;ortholog | DNA-DIRECTED RNA POLYMERASE II SUBUNIT RPB7 (PTHR12709:SF4) |
| ENSSSCT00000052062 | 0.39671 | 0.00779 | Inosine triphosphate pyrophosphatase;ITPA;ortholog | INOSINE TRIPHOSPHATE PYROPHOSPHATASE (PTHR11067:SF9) |
| ENSSSCT00000019290 | 0.40472 | 0.03980 | C-C motif chemokine 2;CCL2;ortholog | C-C MOTIF CHEMOKINE 2 (PTHR12015:SF98) |
| ENSSSCT00000016695 | 0.40501 | 0.03596 | Tissue factor pathway inhibitor;TFPI2;ortholog | TISSUE FACTOR PATHWAY INHIBITOR 2 (PTHR10083:SF336) |
| ENSSSCT00000013313 | 0.41287 | 0.00362 | Peroxiredoxin 4;PRDX4;ortholog | PEROXIREDOXIN-4 (PTHR10681:SF128) |
| ENSSSCT00000026012 | 0.41863 | 0.00633 | Uncharacterized protein;LOC102160336;ortholog | UBIQUITIN-CONJUGATING ENZYME E2 E1 (PTHR24068:SF70) |
| ENSSSCT00000061082 | 0.42434 | 0.02340 | Peptidyl-prolyl cis-trans isomerase;PPIL1;ortholog | PEPTIDYL-PROLYL CIS-TRANS ISOMERASE-LIKE 1 (PTHR45625:SF4) |
| ENSSSCT00000055711 | 0.42507 | 0.04520 | Deoxyribonuclease;DNASE1L3;ortholog | DEOXYRIBONUCLEASE GAMMA (PTHR11371:SF32) |
| ENSSSCT00000017369 | 0.43071 | 0.02666 | Histone acetyltransferase type B catalytic subunit;HAT1;ortholog | HISTONE ACETYLTRANSFERASE TYPE B CATALYTIC SUBUNIT (PTHR12046:SF0) |
| ENSSSCT00000011132 | 0.45942 | 0.04973 | Nucleoside-triphosphatase, cancer-related;NTPCR;ortholog | CANCER-RELATED NUCLEOSIDE-TRIPHOSPHATASE (PTHR43146:SF1) |
| ENSSSCT00000018442 | 0.46314 | 0.03170 | ERCC excision repair 8, CSA ubiquitin ligase complex subunit;ERCC8;ortholog | DNA EXCISION REPAIR PROTEIN ERCC-8 (PTHR46202:SF1) |
| ENSSSCT00000010604 | 0.46332 | 0.04862 | Lymphokine-activated killer T-cell-originated protein kinase;PBK;ortholog | LYMPHOKINE-ACTIVATED KILLER T-CELL-ORIGINATED PROTEIN KINASE-RELATED (PTHR43289:SF14) |
| ENSSSCT00000030730 | 0.52759 | 0.02117 | Acylphosphatase;ACYP2;ortholog | ACYLPHOSPHATASE-2 (PTHR10029:SF3) |
| ENSSSCT00000047604 | 0.57233 | 0.04365 | Protection of telomeres protein 1;POT1;ortholog | PROTECTION OF TELOMERES PROTEIN 1 (PTHR14513:SF0) |
| ENSSSCT00000010246 | 0.57237 | 0.04669 | Replication factor C subunit 3;RFC3;ortholog | REPLICATION FACTOR C SUBUNIT 3 (PTHR11669:SF1) |
| ENSSSCT00000059621 | 0.57394 | 0.00922 | DNA-(apurinic or apyrimidinic site) lyase;APEX1;ortholog | DNA-(APURINIC OR APYRIMIDINIC SITE) LYASE (PTHR22748:SF6) |
| ENSSSCT00000050632 | 0.58692 | 0.04277 | Cell division cycle 2 variant 1;CDC2;ortholog | CYCLIN-DEPENDENT KINASE 1 (PTHR24056:SF334) |
| ENSSSCT00000025604 | 0.62460 | 0.04597 | Aurora kinase B;AURKB;ortholog | AURORA KINASE B (PTHR24350:SF4) |
| ENSSSCT00000017172 | 0.63939 | 0.03784 | Nei like DNA glycosylase 3;NEIL3;ortholog | ENDONUCLEASE 8-LIKE 3 (PTHR22993:SF10) |
| ENSSSCT00000006347 | 0.67016 | 0.00572 | Haloacid dehalogenase like hydrolase domain containing 2;HDHD2;ortholog | HALOACID DEHALOGENASE-LIKE HYDROLASE DOMAIN-CONTAINING PROTEIN 2 (PTHR19288:SF43) |
| ENSSSCT00000005524 | 0.68544 | 0.03833 | DNA polymerase epsilon subunit;POLE2;ortholog | DNA POLYMERASE EPSILON SUBUNIT 2 (PTHR12708:SF0) |
| ENSSSCT00000012916 | 0.68582 | 0.00218 | Replication factor C subunit 4;RFC4;ortholog | REPLICATION FACTOR C SUBUNIT 4 (PTHR11669:SF20) |
| ENSSSCT00000014565 | 0.87289 | 0.03362 | Gamma-butyrobetaine hydroxylase 1;BBOX1;ortholog | GAMMA-BUTYROBETAINE DIOXYGENASE (PTHR10696:SF33) |
| ENSSSCT00000016226 | 0.90692 | 0.01230 | Uncharacterized protein;ACER3;ortholog | ALKALINE CERAMIDASE 3 (PTHR46187:SF3) |
| ENSSSCT00000025069 | 0.94954 | 0.01143 | Limb and CNS expressed 1;LIX1;ortholog | PROTEIN LIMB EXPRESSION 1 HOMOLOG (PTHR31139:SF5) |
| ENSSSCT00000048761 | 0.96095 | 0.03069 | Chitinase-3-like protein 1;CHI3L1;ortholog | CHITINASE-3-LIKE PROTEIN 1 (PTHR11177:SF202) |
| ENSSSCT00000017304 | 1.16837 | 0.02887 | Proteasome 26S subunit, non-ATPase 14;PSMD14;ortholog | 26S PROTEASOME NON-ATPASE REGULATORY SUBUNIT 14 (PTHR10410:SF5) |
| ENSSSCT00000016571 | 1.26785 | 0.02370 | Hepatic and glial cell adhesion molecule;HEPACAM;ortholog | HEPATOCYTE CELL ADHESION MOLECULE (PTHR12080:SF59) |
| ENSSSCT00000042090 | 3.37030 | 0.02763 | Caspase-3;CASP3;ortholog | CASPASE-3 (PTHR10454:SF198) |
|  |  |  |  |  |
| **Zn100 vs Zn50** | | | | |
| ENSSSCT00000044992 | -3.02319 | 0.03912 | Chemokine-like receptor 1;CMLKR1;ortholog | CHEMOKINE-LIKE RECEPTOR 1 (PTHR24225:SF49) |
| ENSSSCT00000057614 | -2.02929 | 0.02688 | SURP and G-patch domain containing 2;SUGP2;ortholog | SURP AND G-PATCH DOMAIN-CONTAINING PROTEIN 2 (PTHR23340:SF2) |
| ENSSSCT00000057045 | -2.01643 | 0.03363 | Elongation of very long chain fatty acids protein 6;ELOVL6;ortholog | ELONGATION OF VERY LONG CHAIN FATTY ACIDS PROTEIN 6 (PTHR11157:SF122) |
| ENSSSCT00000037435 | -1.37823 | 0.03206 | Kynurenine aminotransferase 3;KYAT3;ortholog | KYNURENINE--OXOGLUTARATE TRANSAMINASE 3 (PTHR43807:SF6) |
| ENSSSCT00000061852 | -1.34564 | 0.04091 | Apolipoprotein A-IV;APOA4;ortholog | APOLIPOPROTEIN A-IV (PTHR18976:SF1) |
| ENSSSCT00000036127 | -1.06317 | 0.03309 | Palmitoyltransferase;ZDHHC9;ortholog | PALMITOYLTRANSFERASE ZDHHC9 (PTHR22883:SF71) |
| ENSSSCT00000002555 | -0.93702 | 0.01764 | Arginase;ARG2;ortholog | ARGINASE-2, MITOCHONDRIAL (PTHR43782:SF4) |
| ENSSSCT00000017665 | -0.77952 | 0.02824 | Long-chain-fatty-acid--CoA ligase 3;ACSL3;ortholog | LONG-CHAIN-FATTY-ACID--COA LIGASE 3 (PTHR43272:SF13) |
| ENSSSCT00000031638 | -0.77513 | 0.02287 | Uncharacterized protein;KDM4A;ortholog | LYSINE-SPECIFIC DEMETHYLASE 4A (PTHR10694:SF32) |
| ENSSSCT00000034762 | -0.66305 | 0.03695 | Bone marrow stromal cell antigen 1;BST1;ortholog | ADP-RIBOSYL CYCLASE/CYCLIC ADP-RIBOSE HYDROLASE 2 (PTHR10912:SF4) |
| ENSSSCT00000037199 | -0.62073 | 0.01569 | Dual specificity protein phosphatase;DUSP6;ortholog | DUAL SPECIFICITY PROTEIN PHOSPHATASE 6 (PTHR10159:SF45) |
| ENSSSCT00000064876 | -0.56596 | 0.04492 | Caspase-3;CASP3;ortholog | CASPASE-3 (PTHR10454:SF198) |
| ENSSSCT00000005151 | -0.29461 | 0.01830 | Sulfide:quinone oxidoreductase, mitochondrial;SQOR;ortholog | SULFIDE:QUINONE OXIDOREDUCTASE, MITOCHONDRIAL (PTHR10632:SF2) |
| ENSSSCT00000027876 | -0.23963 | 0.04898 | Dynein light chain;DYNLL2;ortholog | DYNEIN LIGHT CHAIN 2, CYTOPLASMIC (PTHR11886:SF35) |
| ENSSSCT00000016593 | 0.30848 | 0.02281 | SRP receptor alpha subunit;SRPRA;ortholog | SIGNAL RECOGNITION PARTICLE RECEPTOR SUBUNIT ALPHA (PTHR43134:SF1) |
| ENSSSCT00000037748 | 0.56547 | 0.04722 | Hexosyltransferase;CHSY1;ortholog | CHONDROITIN SULFATE SYNTHASE 1 (PTHR12369:SF42) |
| ENSSSCT00000035892 | 0.64936 | 0.03269 | Putative deoxyribonuclease TATDN1;TATDN1;ortholog | DEOXYRIBONUCLEASE TATDN1-RELATED (PTHR10060:SF15) |
| ENSSSCT00000023362 | 0.80443 | 0.03611 | G protein-coupled receptor 174;GPR174;ortholog | G-PROTEIN COUPLED RECEPTOR 174-RELATED (PTHR24232:SF86) |
| ENSSSCT00000046880 | 0.84049 | 0.02973 | Putative P2Y purinoceptor 10;P2RY10;ortholog | P2Y PURINOCEPTOR 10-RELATED (PTHR24232:SF47) |
| ENSSSCT00000062440 | 1.00587 | 0.02237 | Diacylglycerol kinase alpha;DGKA;ortholog | DIACYLGLYCEROL KINASE ALPHA (PTHR11255:SF38) |
| ENSSSCT00000051344 | 1.36096 | 0.00264 | Tetratricopeptide repeat domain 9C;TTC9C;ortholog | TETRATRICOPEPTIDE REPEAT PROTEIN 9C (PTHR43811:SF1) |
| ENSSSCT00000037555 | 1.65413 | 0.03261 | Carbohydrate sulfotransferase;CHST10;ortholog | CARBOHYDRATE SULFOTRANSFERASE 10 (PTHR12137:SF2) |
| ENSSSCT00000000055 | 1.76981 | 0.00911 | Alpha-N-acetylgalactosaminidase precursor;NAGA;ortholog | ALPHA-N-ACETYLGALACTOSAMINIDASE (PTHR11452:SF25) |
| ENSSSCT00000050638 | 1.86635 | 0.04735 | HtrA serine peptidase 4;HTRA4;ortholog | SERINE PROTEASE HTRA4 (PTHR22939:SF105) |
| ENSSSCT00000037095 | 2.37587 | 0.03628 | Hexokinase 3;HK3;ortholog | HEXOKINASE-3 (PTHR19443:SF1) |
|  |  |  |  |  |

**Supplementary Table 7. Genes related to molecular function regulators**

| **ID** | **Log2 Fold Change** | **p-value** | **Gene Name** | **Panther Family** |
| --- | --- | --- | --- | --- |
| **ZnR vs Zn100** | | | | |
| ENSSSCT00000047481 | -2.40795 | 0.03062 | Uncharacterized protein;EIF5;ortholog | EUKARYOTIC TRANSLATION INITIATION FACTOR 5 (PTHR23001:SF7) |
| ENSSSCT00000060340 | -2.54961 | 0.04529 | Acyl-CoA-binding protein;DBI;ortholog | ACYL-COA-BINDING PROTEIN (PTHR23310:SF54) |
| ENSSSCT00000062485 | -1.07307 | 0.01701 | ADP-ribosylation factor GTPase-activating protein 2;ARFGAP2;ortholog | ADP-RIBOSYLATION FACTOR GTPASE-ACTIVATING PROTEIN 2 (PTHR45686:SF8) |
| ENSSSCT00000009844 | -0.66859 | 0.01427 | Cyclin I;CCNI;ortholog | CYCLIN-I (PTHR10177:SF187) |
| ENSSSCT00000025596 | -0.70848 | 0.01267 | Cyclin-dependent kinases regulatory subunit;CKS2;ortholog | CYCLIN-DEPENDENT KINASES REGULATORY SUBUNIT 2 (PTHR23415:SF35) |
|  |  |  |  |  |
| **ZnR vs Zn50** | | | | |
| ENSSSCT00000050048 | -0.85076 | 0.03486 | Uncharacterized protein;MOB3B;ortholog | MOB KINASE ACTIVATOR 3B (PTHR22599:SF22) |
| ENSSSCT00000019029 | -0.76280 | 0.03490 | Rap guanine nucleotide exchange factor like 1;RAPGEFL1;ortholog | RAP GUANINE NUCLEOTIDE EXCHANGE FACTOR-LIKE 1 (PTHR23113:SF230) |
| ENSSSCT00000054120 | -0.72672 | 0.00449 | LLGL2, scribble cell polarity complex component;LLGL2;ortholog | LETHAL(2) GIANT LARVAE PROTEIN HOMOLOG 2 (PTHR10241:SF20) |
| ENSSSCT00000003272 | -0.70587 | 0.02785 | Signal induced proliferation associated 1 like 3;SIPA1L3;ortholog | SIGNAL-INDUCED PROLIFERATION-ASSOCIATED 1-LIKE PROTEIN 3 (PTHR15711:SF15) |
| ENSSSCT00000012494 | -0.70221 | 0.00237 | CISH;CISH_tv2;ortholog | CYTOKINE-INDUCIBLE SH2-CONTAINING PROTEIN (PTHR10155:SF9) |
| ENSSSCT00000048990 | -0.68387 | 0.01484 | Myosin ID;MYO1D;ortholog | UNCONVENTIONAL MYOSIN-ID (PTHR13140:SF417) |
| ENSSSCT00000014043 | -0.68357 | 0.01091 | EPS8 like 2;EPS8L2;ortholog | EPIDERMAL GROWTH FACTOR RECEPTOR KINASE SUBSTRATE 8-LIKE PROTEIN 2 (PTHR12287:SF20) |
| ENSSSCT00000024524 | -0.59541 | 0.01004 | Uncharacterized protein;PPP4R1;ortholog | SERINE/THREONINE-PROTEIN PHOSPHATASE 4 REGULATORY SUBUNIT 1 (PTHR10648:SF8) |
| ENSSSCT00000029636 | -0.50558 | 0.04890 | Suppressor of cytokine signaling 1;socs1;ortholog | SUPPRESSOR OF CYTOKINE SIGNALING 1 (PTHR10155:SF4) |
| ENSSSCT00000053600 | -0.46518 | 0.04157 | Rho GTPase activating protein 17;ARHGAP17;ortholog | RHO GTPASE-ACTIVATING PROTEIN 17 (PTHR14130:SF3) |
| ENSSSCT00000004747 | 0.40291 | 0.00237 | Sorting nexin 14;SNX14;ortholog | SORTING NEXIN-14 (PTHR22775:SF3) |
| ENSSSCT00000016695 | 0.40501 | 0.03596 | Tissue factor pathway inhibitor;TFPI2;ortholog | TISSUE FACTOR PATHWAY INHIBITOR 2 (PTHR10083:SF336) |
| ENSSSCT00000060340 | 0.44679 | 0.00174 | Acyl-CoA-binding protein;DBI;ortholog | ACYL-COA-BINDING PROTEIN (PTHR23310:SF54) |
| ENSSSCT00000047604 | 0.57233 | 0.04365 | Protection of telomeres protein 1;POT1;ortholog | PROTECTION OF TELOMERES PROTEIN 1 (PTHR14513:SF0) |
|  |  |  |  |  |
| **Zn100 vs Zn50** | | | | |
| ENSSSCT00000015136 | -2.11392 | 0.02481 | Epidermal growth factor receptor pathway substrate 15 like 1;EPS15L1;ortholog | EPIDERMAL GROWTH FACTOR RECEPTOR SUBSTRATE 15-LIKE 1 (PTHR11216:SF69) |
| ENSSSCT00000061852 | -1.34564 | 0.04091 | Apolipoprotein A-IV;APOA4;ortholog | APOLIPOPROTEIN A-IV (PTHR18976:SF1) |
| ENSSSCT00000004747 | 0.30838 | 0.03363 | Sorting nexin 14;SNX14;ortholog | SORTING NEXIN-14 (PTHR22775:SF3) |
| ENSSSCT00000056812 | 1.50879 | 0.04026 | Regulation of nuclear pre-mRNA domain containing 1A;RPRD1A;ortholog | REGULATION OF NUCLEAR PRE-MRNA DOMAIN-CONTAINING PROTEIN 1A (PTHR12460:SF2) |
|  |  |  |  |  |

**Supplementary Table 8. Genes related to molecular transducer activity**

| **ID** | **Log2 Fold Change** | **pvalue** | **Gene Name** | **Panther Family** |
| --- | --- | --- | --- | --- |
| **ZnR vs Zn100** | | | | |
| ENSSSCT00000044729 | -2.8201 | 0.0281 | Somatostatin receptor type 5;SSTR5;ortholog | SOMATOSTATIN RECEPTOR TYPE 5 (PTHR24229:SF20) |
| ENSSSCT00000065484 | -1.1044 | 0.0385 | Midkine precursor;MDK;ortholog | MIDKINE (PTHR13850:SF2) |
| ENSSSCT00000009844 | -0.6686 | 0.0143 | Cyclin I;CCNI;ortholog | CYCLIN-I (PTHR10177:SF187) |
| ENSSSCT00000025596 | 0.4149 | 0.0316 | Cyclin-dependent kinases regulatory subunit;CKS2;ortholog | CYCLIN-DEPENDENT KINASES REGULATORY SUBUNIT 2 (PTHR23415:SF35) |
| ENSSSCT00000060061 | 0.8021 | 0.0453 | Regenerating islet derived family member 4 variant 2;REG4;ortholog | REGENERATING ISLET-DERIVED PROTEIN 4 (PTHR22803:SF116) |
| ENSSSCT00000010221 | 0.9548 | 0.0191 | High mobility group protein B1;HMGB1;ortholog | SUBFAMILY NOT NAMED (PTHR13711:SF328) |
| ENSSSCT00000053543 | 1.3745 | 0.0077 | Chemokine C-C motif receptor-like 2;CCRL2;ortholog | C-C CHEMOKINE RECEPTOR-LIKE 2 (PTHR10489:SF655) |
|  |  |  |  |  |
| **ZnR vs Zn50** | | | | |
| ENSSSCT00000005664 | -1.6456 | 0.0229 | Interferon epsilon;IFNE;ortholog | INTERFERON EPSILON (PTHR11691:SF8) |
| ENSSSCT00000065647 | -0.9823 | 0.0063 | Plexin B2;PLXNB2;ortholog | PLEXIN-B2 (PTHR22625:SF9) |
| ENSSSCT00000027155 | -0.6326 | 0.0417 | Guanine nucleotide-binding protein subunit alpha-11;GNA11;ortholog | GUANINE NUCLEOTIDE-BINDING PROTEIN SUBUNIT ALPHA-11 (PTHR10218:SF57) |
| ENSSSCT00000016661 | -0.5012 | 0.0462 | Cyclin dependent kinase 18;CDK18;ortholog | CYCLIN-DEPENDENT KINASE 18 (PTHR24056:SF52) |
| ENSSSCT00000003721 | -0.4456 | 0.0254 | Uncharacterized protein;CDK11B;ortholog | CYCLIN-DEPENDENT KINASE 11A-RELATED (PTHR24056:SF107) |
| ENSSSCT00000028348 | 0.3593 | 0.0467 | Cyclin-dependent kinase 4;CDK4;ortholog | CYCLIN-DEPENDENT KINASE 4 (PTHR24056:SF129) |
| ENSSSCT00000019290 | 0.4047 | 0.0398 | C-C motif chemokine 2;CCL2;ortholog | C-C MOTIF CHEMOKINE 2 (PTHR12015:SF98) |
| ENSSSCT00000050632 | 0.5869 | 0.0428 | Cell division cycle 2 variant 1;CDC2;ortholog | CYCLIN-DEPENDENT KINASE 1 (PTHR24056:SF334) |
| ENSSSCT00000064385 | 0.7337 | 0.0228 | C-X-C chemokine receptor type 4;CXCR4;ortholog | C-X-C CHEMOKINE RECEPTOR TYPE 4 (PTHR10489:SF594) |
| ENSSSCT00000016464 | 2.3630 | 0.0478 | C-X-C motif chemokine receptor 5;CXCR5;ortholog | C-X-C CHEMOKINE RECEPTOR TYPE 5 (PTHR10489:SF618) |
|  |  |  |  |  |
| **Zn100 vs Zn50** | | | | |
| ENSSSCT00000044992 | -3.0232 | 0.0391 | Chemokine-like receptor 1;CMLKR1;ortholog | CHEMOKINE-LIKE RECEPTOR 1 (PTHR24225:SF49) |
| ENSSSCT00000023362 | 0.8044 | 0.0361 | G protein-coupled receptor 174;GPR174;ortholog | G-PROTEIN COUPLED RECEPTOR 174-RELATED (PTHR24232:SF86) |
| ENSSSCT00000030552 | 0.8145 | 0.0480 | Chemokine C-C motif receptor-like 2;CCRL2;ortholog | C-C CHEMOKINE RECEPTOR-LIKE 2 (PTHR10489:SF655) |
| ENSSSCT00000046880 | 0.8405 | 0.0297 | Putative P2Y purinoceptor 10;P2RY10;ortholog | P2Y PURINOCEPTOR 10-RELATED (PTHR24232:SF47) |
| ENSSSCT00000016464 | 2.2650 | 0.0278 | C-X-C motif chemokine receptor 5;CXCR5;ortholog | C-X-C CHEMOKINE RECEPTOR TYPE 5 (PTHR10489:SF618) |
|  |  |  |  |  |

**Supplementary Table 9. Genes related to structural molecular activity**

| **ID** | **Log2 Fold Change** | **p-value** | **Gene Name** | **Panther Family** |
| --- | --- | --- | --- | --- |
| **ZnR vs Zn100** | | | | |
| ENSSSCT00000049323 | -1.5859 | 0.0057 | Transgelin;TAGLN2;ortholog | TRANSGELIN-2 (PTHR18959:SF41) |
| ENSSSCT00000063010 | 0.2053 | 0.0402 | 60S ribosomal protein L3;RPL3;ortholog | 60S RIBOSOMAL PROTEIN L3 (PTHR11363:SF4) |
| ENSSSCT00000053256 | 0.2441 | 0.0465 | 60S ribosomal protein L6;RPL6;ortholog | 60S RIBOSOMAL PROTEIN L6 (PTHR10715:SF0) |
| ENSSSCT00000005457 | 0.2467 | 0.0258 | 60S ribosomal protein L4;RPL4;ortholog | 60S RIBOSOMAL PROTEIN L4 (PTHR19431:SF0) |
| ENSSSCT00000010191 | 0.2670 | 0.0079 | 60S ribosomal protein L21;RPL21;ortholog | 60S RIBOSOMAL PROTEIN L21 (PTHR20981:SF8) |
| ENSSSCT00000024316 | 0.3078 | 0.0332 | Mitochondrial ribosomal protein L27;MRPL27;ortholog | 39S RIBOSOMAL PROTEIN L27, MITOCHONDRIAL (PTHR15893:SF0) |
| ENSSSCT00000022802 | 0.3094 | 0.0322 | 60S ribosomal protein L11;RPL11;ortholog | SUBFAMILY NOT NAMED (PTHR11994:SF27) |
| ENSSSCT00000065434 | 0.3116 | 0.0195 | Mitochondrial ribosomal protein L51;MRPL51;ortholog | 39S RIBOSOMAL PROTEIN L51, MITOCHONDRIAL (PTHR13409:SF0) |
| ENSSSCT00000034332 | 0.3873 | 0.0366 | 39S ribosomal protein L13, mitochondrial;MRPL13;ortholog | 39S RIBOSOMAL PROTEIN L13, MITOCHONDRIAL (PTHR11545:SF17) |
| ENSSSCT00000043395 | 0.3874 | 0.0075 | Actin-related protein 3;ACTR3;ortholog | ACTIN-RELATED PROTEIN 3 (PTHR11937:SF175) |
| ENSSSCT00000001703 | 0.3959 | 0.0244 | Ribosomal protein S10;RPS10;ortholog | 40S RIBOSOMAL PROTEIN S10 (PTHR12146:SF0) |
|  |  |  |  |  |
| **ZnR vs Zn50** | | | | |
| ENSSSCT00000049323 | -1.3872 | 0.0395 | Transgelin;TAGLN2;ortholog | TRANSGELIN-2 (PTHR18959:SF41) |
| ENSSSCT00000066126 | -0.7982 | 0.0128 | Adseverin;SCIN;ortholog | ADSEVERIN (PTHR11977:SF78) |
| ENSSSCT00000048990 | -0.6839 | 0.0148 | Myosin ID;MYO1D;ortholog | UNCONVENTIONAL MYOSIN-ID (PTHR13140:SF417) |
| ENSSSCT00000017637 | -0.6543 | 0.0250 | Villin-1;VIL1;ortholog | VILLIN-1 (PTHR11977:SF35) |
| ENSSSCT00000022558 | -0.6180 | 0.0251 | MAL-like protein;MALL;ortholog | MAL-LIKE PROTEIN (PTHR22776:SF24) |
| ENSSSCT00000015666 | -0.5137 | 0.0399 | Catenin alpha 1;CTNNA1;ortholog | CATENIN ALPHA-1 (PTHR18914:SF24) |
| ENSSSCT00000018499 | 0.2372 | 0.0453 | 60S ribosomal protein L26-like 1;RPL26L1;ortholog | 60S RIBOSOMAL PROTEIN L26-LIKE 1 (PTHR11143:SF10) |
| ENSSSCT00000051843 | 0.2404 | 0.0477 | 60S ribosomal protein L23;RPL23;ortholog | 60S RIBOSOMAL PROTEIN L23 (PTHR11761:SF8) |
| ENSSSCT00000017625 | 0.2633 | 0.0145 | Arp2/3 complex 34 kDa subunit;ARPC2;ortholog | ACTIN-RELATED PROTEIN 2/3 COMPLEX SUBUNIT 2 (PTHR12058:SF0) |
| ENSSSCT00000010191 | 0.2704 | 0.0415 | 60S ribosomal protein L21;RPL21;ortholog | 60S RIBOSOMAL PROTEIN L21 (PTHR20981:SF8) |
| ENSSSCT00000063010 | 0.2815 | 0.0240 | 60S ribosomal protein L3;RPL3;ortholog | 60S RIBOSOMAL PROTEIN L3 (PTHR11363:SF4) |
| ENSSSCT00000016207 | 0.2853 | 0.0440 | 40S ribosomal protein S3;RPS3;ortholog | 40S RIBOSOMAL PROTEIN S3 (PTHR11760:SF32) |
| ENSSSCT00000004346 | 0.2872 | 0.0301 | 40S ribosomal protein S8;RPS8;ortholog | 40S RIBOSOMAL PROTEIN S8 (PTHR10394:SF3) |
| ENSSSCT00000001849 | 0.3198 | 0.0463 | 39S ribosomal protein L2, mitochondrial;MRPL2;ortholog | 39S RIBOSOMAL PROTEIN L2, MITOCHONDRIAL (PTHR13691:SF5) |
| ENSSSCT00000015437 | 0.3314 | 0.0297 | 40S ribosomal protein S23;RPS23;ortholog | 40S RIBOSOMAL PROTEIN S23 (PTHR11652:SF45) |
| ENSSSCT00000049763 | 0.3336 | 0.0146 | 60S acidic ribosomal protein P0;RPLP0;ortholog | 60S ACIDIC RIBOSOMAL PROTEIN P0-RELATED (PTHR45699:SF1) |
| ENSSSCT00000024602 | 0.4030 | 0.0151 | 40S ribosomal protein S16;RPS16;ortholog | 40S RIBOSOMAL PROTEIN S16 (PTHR21569:SF4) |
| ENSSSCT00000022802 | 0.4136 | 0.0085 | 60S ribosomal protein L11;RPL11;ortholog | SUBFAMILY NOT NAMED (PTHR11994:SF27) |
| ENSSSCT00000054538 | 0.4148 | 0.0178 | 40S ribosomal protein S9;RPS9;ortholog | 40S RIBOSOMAL PROTEIN S9 (PTHR11831:SF5) |
| ENSSSCT00000024316 | 0.4239 | 0.0025 | Mitochondrial ribosomal protein L27;MRPL27;ortholog | 39S RIBOSOMAL PROTEIN L27, MITOCHONDRIAL (PTHR15893:SF0) |
| ENSSSCT00000043395 | 0.4362 | 0.0171 | Actin-related protein 3;ACTR3;ortholog | ACTIN-RELATED PROTEIN 3 (PTHR11937:SF175) |
| ENSSSCT00000000203 | 0.4501 | 0.0195 | Tubulin alpha-1B chain;TUBA1B;ortholog | TUBULIN ALPHA-1B CHAIN (PTHR11588:SF352) |
| ENSSSCT00000000963 | 0.4595 | 0.0154 | ARP6 actin related protein 6 homolog;ACTR6;ortholog | ACTIN-RELATED PROTEIN 6 (PTHR11937:SF47) |
| ENSSSCT00000012334 | 0.4622 | 0.0467 | 40S ribosomal protein SA;RPSA;ortholog | 40S RIBOSOMAL PROTEIN SA (PTHR11489:SF9) |
| ENSSSCT00000034332 | 0.5062 | 0.0066 | 39S ribosomal protein L13, mitochondrial;MRPL13;ortholog | 39S RIBOSOMAL PROTEIN L13, MITOCHONDRIAL (PTHR11545:SF17) |
| ENSSSCT00000046422 | 0.8902 | 0.0224 | S1 RNA binding domain 1;SRBD1;ortholog | S1 RNA-BINDING DOMAIN-CONTAINING PROTEIN 1 (PTHR10724:SF1) |
|  |  |  |  |  |
| **Zn100 vs Zn50** | | | | |
| ENSSSCT00000048062 | 0.6858 | 0.04567 | Heterogeneous nuclear ribonucleoprotein F;HNRNPF;ortholog | HETEROGENEOUS NUCLEAR RIBONUCLEOPROTEIN F (PTHR13976:SF32) |
|  |  |  |  |  |

**Supplementary Table 10. Genes related to transcription regulator activity**

| **ID** | **Log2 Fold Change** | **p-value** | **Gene Name** | **Panther Family** |
| --- | --- | --- | --- | --- |
| **ZnR vs Zn100** | | | | |
| ENSSSCT00000038564 | -1.0564 | 0.0183 | Transducin beta like 1 X-linked receptor 1;TBL1XR1;ortholog | F-BOX-LIKE/WD REPEAT-CONTAINING PROTEIN TBL1XR1 (PTHR22846:SF40) |
| ENSSSCT00000042890 | -0.6305 | 0.0439 | MAF bZIP transcription factor B;MAFB;ortholog | TRANSCRIPTION FACTOR MAFB (PTHR10129:SF10) |
| ENSSSCT00000006644 | -0.4141 | 0.0239 | Krueppel-like factor 10;KLF10;ortholog | KRUEPPEL-LIKE FACTOR 10 (PTHR23235:SF64) |
| ENSSSCT00000002569 | 0.2227 | 0.0429 | Enhancer of rudimentary homolog;ERH;ortholog | ENHANCER OF RUDIMENTARY HOMOLOG (PTHR12373:SF0) |
| ENSSSCT00000055436 | 0.4451 | 0.0352 | Interferon regulatory factor 1;IRF1;ortholog | INTERFERON REGULATORY FACTOR 1 (PTHR11949:SF3) |
| ENSSSCT00000010221 | 0.9548 | 0.0191 | High mobility group protein B1;HMGB1;ortholog | SUBFAMILY NOT NAMED (PTHR13711:SF328) |
| ENSSSCT00000000461 | 1.0827 | 0.0202 | Signal transducer and activator of transcription;STAT6;ortholog | SIGNAL TRANSDUCER AND ACTIVATOR OF TRANSCRIPTION 6 (PTHR11801:SF48) |
| ENSSSCT00000043331 | 1.4202 | 0.0017 | TATA-box binding protein associated factor 9;TAF9;ortholog | TRANSCRIPTION INITIATION FACTOR TFIID SUBUNIT 9 (PTHR12595:SF8) |
| ENSSSCT00000045699 | 1.9738 | 0.0499 | Myocyte-specific enhancer factor 2C;MEF2C;ortholog | MYOCYTE-SPECIFIC ENHANCER FACTOR 2C (PTHR11945:SF25) |
|  |  |  |  |  |
| **ZnR vs Zn50** | | | | |
| ENSSSCT00000011953 | -0.8812 | 0.0004 | E74 like ETS transcription factor 3;ELF3;ortholog | ETS-RELATED TRANSCRIPTION FACTOR ELF-3 (PTHR11849:SF13) |
| ENSSSCT00000042890 | -0.8785 | 0.0022 | MAF bZIP transcription factor B;MAFB;ortholog | TRANSCRIPTION FACTOR MAFB (PTHR10129:SF10) |
| ENSSSCT00000002141 | -0.8766 | 0.0225 | Promyelocytic leukemia;PML;ortholog | PROTEIN PML (PTHR25462:SF241) |
| ENSSSCT00000052285 | -0.8527 | 0.0190 | Retinoic acid receptor RXR-alpha;RXRA;ortholog | RETINOIC ACID RECEPTOR RXR-ALPHA (PTHR24083:SF39) |
| ENSSSCT00000019093 | -0.8246 | 0.0257 | Homeobox B6;HOXB6;ortholog | HOMEOBOX PROTEIN HOX-B6 (PTHR45659:SF9) |
| ENSSSCT00000001722 | -0.7747 | 0.0405 | Transcriptional enhancer factor TEF-5;TEAD3;ortholog | TRANSCRIPTIONAL ENHANCER FACTOR TEF-5 (PTHR11834:SF0) |
| ENSSSCT00000060403 | -0.7709 | 0.0169 | Replication initiator 1;REPIN1;ortholog | ZINC FINGER PROTEIN GLI4 (PTHR24388:SF74) |
| ENSSSCT00000062592 | -0.7382 | 0.0481 | Signal transducer and activator of transcription;stat5B;ortholog | SIGNAL TRANSDUCER AND ACTIVATOR OF TRANSCRIPTION 5B (PTHR11801:SF39) |
| ENSSSCT00000008270 | -0.7138 | 0.0054 | GATA binding protein 5;GATA5;ortholog | TRANSCRIPTION FACTOR GATA-5 (PTHR10071:SF289) |
| ENSSSCT00000018974 | -0.6978 | 0.0030 | Junction plakoglobin;JUP;ortholog | JUNCTION PLAKOGLOBIN (PTHR45976:SF3) |
| ENSSSCT00000035232 | -0.6798 | 0.0328 | Interferon regulatory factor 7;Irf7;ortholog | INTERFERON REGULATORY FACTOR 7 (PTHR11949:SF2) |
| ENSSSCT00000008291 | -0.6649 | 0.0080 | MAF bZIP transcription factor K;MAFK;ortholog | TRANSCRIPTION FACTOR MAFK (PTHR10129:SF26) |
| ENSSSCT00000010207 | -0.6429 | 0.0055 | Homeobox protein CDX-2;CDX2;ortholog | HOMEOBOX PROTEIN CDX-2 (PTHR24332:SF27) |
| ENSSSCT00000006567 | -0.6370 | 0.0318 | Zinc fingers and homeoboxes 1;ZHX1;ortholog | ZINC FINGERS AND HOMEOBOXES PROTEIN 1 (PTHR15467:SF4) |
| ENSSSCT00000004101 | -0.6258 | 0.0303 | Transcription factor GATA-6;GATA6;ortholog | TRANSCRIPTION FACTOR GATA-6 (PTHR10071:SF23) |
| ENSSSCT00000015600 | -0.6177 | 0.0306 | Interferon regulatory factor 1;IRF1;ortholog | INTERFERON REGULATORY FACTOR 1 (PTHR11949:SF3) |
| ENSSSCT00000015156 | -0.5345 | 0.0053 | Nuclear receptor subfamily 2 group F member 6;NR2F6;ortholog | NUCLEAR RECEPTOR SUBFAMILY 2 GROUP F MEMBER 6 (PTHR24083:SF44) |
| ENSSSCT00000034737 | -0.5297 | 0.0463 | Interferon regulatory factor 6;IRF6;ortholog | INTERFERON REGULATORY FACTOR 6 (PTHR11949:SF9) |
| ENSSSCT00000015792 | -0.5196 | 0.0420 | Caudal type homeobox 1;CDX1;ortholog | HOMEOBOX PROTEIN CDX-1 (PTHR24332:SF16) |
| ENSSSCT00000045268 | -0.4626 | 0.0436 | ETS2 repressor factor;ERF;ortholog | ETS DOMAIN-CONTAINING TRANSCRIPTION FACTOR ERF (PTHR11849:SF31) |
| ENSSSCT00000002240 | -0.4478 | 0.0025 | Interferon regulatory factor 9;IRF9;ortholog | INTERFERON REGULATORY FACTOR 9 (PTHR11949:SF26) |
| ENSSSCT00000030348 | -0.3796 | 0.0161 | WW domain binding protein 2;WBP2;ortholog | WW DOMAIN-BINDING PROTEIN 2 (PTHR31606:SF4) |
| ENSSSCT00000052410 | -0.3716 | 0.0463 | ETS proto-oncogene 2, transcription factor;ETS2;ortholog | PROTEIN C-ETS-2 (PTHR11849:SF188) |
| ENSSSCT00000056298 | -0.3552 | 0.0456 | Class E basic helix-loop-helix protein 40;BHLHE40;ortholog | CLASS E BASIC HELIX-LOOP-HELIX PROTEIN 40 (PTHR10985:SF3) |
| ENSSSCT00000007085 | -0.2351 | 0.0465 | Heparin binding growth factor;HDGF;ortholog | HEPATOMA-DERIVED GROWTH FACTOR-RELATED (PTHR12550:SF41) |
| ENSSSCT00000002569 | 0.3328 | 0.0030 | Enhancer of rudimentary homolog;ERH;ortholog | ENHANCER OF RUDIMENTARY HOMOLOG (PTHR12373:SF0) |
| ENSSSCT00000042922 | 0.4612 | 0.0434 | Transcription elongation factor A protein 1;TCEA1;ortholog | TRANSCRIPTION ELONGATION FACTOR A PROTEIN 1 (PTHR11477:SF1) |
| ENSSSCT00000011604 | 0.6802 | 0.0299 | SWI5 dependent homologous recombination repair protein 1;SFR1;ortholog | SWI5-DEPENDENT RECOMBINATION DNA REPAIR PROTEIN 1 HOMOLOG (PTHR28643:SF1) |
| ENSSSCT00000045699 | 2.6408 | 0.0168 | Myocyte-specific enhancer factor 2C;MEF2C;ortholog | MYOCYTE-SPECIFIC ENHANCER FACTOR 2C (PTHR11945:SF25) |
|  |  |  |  |  |
| **Zn100 vs Zn50** | | | | |
| ENSSSCT00000031638 | -0.7751 | 0.0229 | Uncharacterized protein;KDM4A;ortholog | LYSINE-SPECIFIC DEMETHYLASE 4A (PTHR10694:SF32) |
| ENSSSCT00000045848 | -0.7564 | 0.0408 | Krueppel-like factor 3;KLF3;ortholog | KRUEPPEL-LIKE FACTOR 3 (PTHR23235:SF48) |
| ENSSSCT00000048182 | -0.7478 | 0.0440 | SAM pointed domain-containing Ets transcription factor;SPDEF;ortholog | SAM POINTED DOMAIN-CONTAINING ETS TRANSCRIPTION FACTOR (PTHR11849:SF182) |
| ENSSSCT00000018974 | -0.4587 | 0.0218 | Junction plakoglobin;JUP;ortholog | JUNCTION PLAKOGLOBIN (PTHR45976:SF3) |
| ENSSSCT00000015156 | -0.4521 | 0.0186 | Nuclear receptor subfamily 2 group F member 6;NR2F6;ortholog | NUCLEAR RECEPTOR SUBFAMILY 2 GROUP F MEMBER 6 (PTHR24083:SF44) |
| ENSSSCT00000040161 | 2.8333 | 0.0088 | Regulatory factor X7;RFX7;ortholog | DNA-BINDING PROTEIN RFX7 (PTHR12619:SF2) |
|  |  |  |  |  |

**Supplementary Table 11. Genes related to translation regulator activity**

| **ID** | **Log2 Fold Change** | **p-value** | **Gene Name** | **Panther Family** |
| --- | --- | --- | --- | --- |
| **ZnR vs Zn100** | | | | |
| ENSSSCT00000047481 | -2.4079 | 0.0306 | Uncharacterized protein;EIF5;ortholog | EUKARYOTIC TRANSLATION INITIATION FACTOR 5 (PTHR23001:SF7) |
| [ENSSSCT00000044665](http://useast.ensembl.org/sus_scrofa/Transcript/Summary?db=core;t=ENSSSCT00000044665) | 0.2862 | 0.0271 | Mitochondrial complement component 1 Q subcomponent-binding protein;C1QBP;ortholog | COMPLEMENT COMPONENT 1 Q SUBCOMPONENT-BINDING PROTEIN, MITOCHONDRIAL (PTHR10826:SF1) |
|  |  |  |  |  |
| **ZnR vs Zn50** | | | | |
| ENSSSCT00000057207 | 0.3581 | 0.0317 | Eukaryotic translation initiation factor 1A, X-chromosomal;EIF1AX;ortholog | EUKARYOTIC TRANSLATION INITIATION FACTOR 1A, X-CHROMOSOMAL (PTHR21668:SF4) |
| ENSSSCT00000017560 | 0.3721 | 0.0072 | Elongation factor 1-beta;EEF1B2;ortholog | ELONGATION FACTOR 1-BETA (PTHR11595:SF21) |
|  |  |  |  |  |
| **Zn100 vs Zn50** | | | | |
| ENSSSCT00000057503 | 1.0832 | 0.01031 | Cold-inducible RNA-binding protein;CIRBP;ortholog | COLD-INDUCIBLE RNA-BINDING PROTEIN (PTHR15241:SF45) |
|  |  |  |  |  |

**
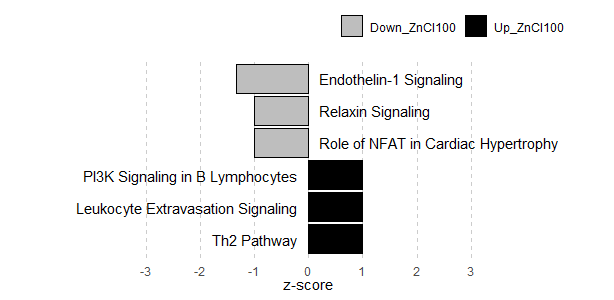
**

**Supplementary figure 1**. Top three upregulated and downregulated canonical pathways through IPA analysis of differentially expressed genes in Zn100 compared with Zn50. Z-score >2 or <-2 were considered significantly upregulated (black columns) or downregulated (grey columns) respectively. n=8 for Zn100 and Zn50.
